# The membrane domain of respiratory complex I accumulates during muscle aging in *Drosophila melanogaster*

**DOI:** 10.1038/s41598-022-26414-5

**Published:** 2022-12-27

**Authors:** Kaniz Fatima Binte Hossain, Anjaneyulu Murari, Bibhuti Mishra, Edward Owusu-Ansah

**Affiliations:** Department of Physiology and Cellular Biophysics, Columbia University Irving Medical Center, New York, NY 10032 USA

**Keywords:** Organelles, Ageing

## Abstract

The boot-shaped respiratory complex I (CI) consists of a mitochondrial matrix and membrane domain organized into N-, Q- and P-modules. The N-module is the most distal part of the matrix domain, whereas the Q-module is situated between the N-module and the membrane domain. The proton-pumping P-module is situated in the membrane domain. We explored the effect of aging on the disintegration of CI and its constituent subcomplexes and modules in *Drosophila* flight muscles. We find that the fully-assembled complex remains largely intact in aged flies. And while the effect of aging on the stability of many Q- and N-module subunits in subcomplexes was stochastic, NDUFS3 was consistently down-regulated in subcomplexes with age. This was associated with an accumulation of many P-module subunits in subcomplexes. The potential significance of these studies is that genetic manipulations aimed at boosting, perhaps, a few CI subunits may suffice to restore the whole CI biosynthesis pathway during muscle aging.

## Introduction

Aging—defined as a progressive functional decline—is a major risk factor for several debilitating diseases. One of the hallmarks of aging is mitochondrial dysfunction^1^. Accordingly, identifying interventions that ameliorate or at least delay mitochondrial dysfunction in model organisms is a crucial need that would likely have therapeutic implications for human aging.

Studies in *Drosophila* have identified several genetic manipulations of mitochondrial proteins that have shown some promise in increasing lifespan. For example, induction of Dynamin-related protein 1 (Drp1), which is a major regulator of mitochondrial fission, during midlife in *Drosophila* increases lifespan^2^. Similarly, forced expression of the yeast alternative NADH:ubiquinone oxidoreductase, which catalyzes the transfer of electrons from NADH to ubiquinone via FAD without pumping protons across the mitochondrial inner membrane increases lifespan^3–5^. Further, overexpression of the alternative NADH:ubiquinone oxidoreductase reduces reactive oxygen species (ROS) production and increases several markers of CI activity^3–5^. Paradoxically, a restrained knockdown of several CI proteins also enhances longevity^6,7^. While the exact mechanisms involved in triggering the increased lifespan caused by mild CI disruption are still being unraveled, compensatory mitochondrial stress signaling cascades seem to contribute prominently. However, the question of whether boosting the expression of individual CI subunits can extend lifespan remains uncharted.

The boot-shaped mitochondrial CI is the most sophisticated component of the oxidative phosphorylation (OXPHOS) system^8,9^. Human CI has 45 subunits organized into two domains of the complex, dubbed the matrix and membrane domains^10^. There are three distinct functional modules of CI known as the N-, Q- and P-modules. The N-module contains the flavin mononucleotide (FMN) cofactor which accepts electrons from NADH in the mitochondrial matrix. The Q-module sits between the N-module and the membrane domain and transfers electrons to ubiquinone. The proton-pumping P-module is essentially the membrane domain [reviewed in^11–13^].

Fourteen core subunits form the catalytic centers of CI and are conserved from the ancestral enzyme in bacteria to the eukaryotic enzyme. Seven core subunits (NDUFS1, NDUFS2, NDUFS3, NDUFS7, NDUFS8, NDUFV1 and NDUFV2) are encoded by the nucleus, translated in the cytoplasm, and imported into the mitochondrion, while the other seven are encoded by mitochondrial DNA (mtDNA) and translated in the mitochondrion (i.e., ND1, ND2, ND3, ND4, ND4L, ND5 and ND6). The 31 remaining subunits are referred to as accessory or supernumerary subunits, as they are not directly involved in performing the bioenergetics functions of CI. During CI assembly, specific subcomplexes consisting of a few CI subunits form largely independently of each other and merge in a stereotypic fashion en route to forming the mature complex^12,14,15^. About a dozen or so CI assembly factors (CIAFs) have been identified. CIAFs are proteins that are usually found in association with specific subcomplexes and assist with the assembly process; but they are subsequently released when assembly is complete. The N-, Q- and P-modules are synthesized from specific subcomplexes or assembly intermediates that can be tracked by immunoblotting or complexome profiling techniques^16–19^.

CI function can deteriorate with age via several mechanisms. First, the complex can disintegrate with age, resulting in a reduction in the amount of CI that can be detected in blue native polyacrylamide gels. Second, the complex can be preserved with age but still result in a deterioration of CI function, perhaps, due to increased protein oxidation or other post-translational modifications on subunits. Third, with the identification of several phospholipid molecules tightly associated with several membrane domain subunits, oxidation of these lipid molecules, could also in principle impair CI function. Indeed, there are many other ways by which CI function can deteriorate with age. Consequently, identifying ways to preserve CI function is essential.

Although the alternative NADH:ubiquinone oxidoreductase from yeast can enhance, restore, or substitute for CI function in multiple organisms, there has been limited enthusiasm for overexpressing individual CI subunits as a possible means to improve CI function^3–5,20–25^. This is largely due to the large number of CI subunits in eukaryotic organisms, making it seem unlikely that forced expression of one or a few CI subunits or CIAFs could improve or ameliorate CI function with age. However, although the proteasome is comparable to CI as it has many subunits, it has been shown in *C. elegans* that overexpression of a single 19S proteasome subunit, RPN-6, is sufficient to confer proteotoxic stress resistance and increase longevity. In addition, overexpression of the human ortholog of RPN-6, PSMD11, is also sufficient to increase both proteasome assembly and activity in human embryonic stem cells^26,27^. As a consequence, we explored the effect of aging on the disintegration or stability of CI and its constituent subcomplexes and modules, with the view that uncovering such information may provide clues for up-regulating specific CI subunits or CIAFs to preserve, restore or enhance CI activity during aging. We find that the fully-assembled complex remains largely intact in 8-week-old flies. Further, aging was associated with an up-regulation of multiple membrane domain subunits—including  some mtDNA-encoded subunits—in subcomplexes. The disintegration or stability of a few subunits was variable. Interestingly, however, NDUFS3, a component of the Q-module, was consistently down-regulated in subcomplexes with age at 25 °C; and Q-module biosynthesis was perturbed in flies exposed to mild thermal stress at 27 °C. As a result, we propose that genetic manipulations aimed at enhancing NDUFS3 expression, and perhaps a few of the subunits that showed stochastic expression changes with age, may be crucial in forestalling CI disintegration under stress or during muscle aging.

## Results

### The amount of fully-assembled CI is not altered during muscle aging or mild thermal stress

In this study, we explored the effect of aging on one of the properties of CI that can impact its function with age—the biogenesis and assembly of CI. We isolated mitochondria from thoraces of adult flies 1 week, 5 weeks and 8 weeks after they eclosed at 25 °C; and solubilized their mitochondrial membranes in digitonin at a ratio of 10 g of digitonin: 3 g of protein. Afterwards, we analyzed the integrity of their OXPHOS complexes using blue native polyacrylamide gel electrophoresis (BN-PAGE) and subsequent Coomassie and silver staining (Fig. S1a,b). Surprisingly, we failed to observe any reproducible changes in the amount of fully-assembled CI or other OXPHOS complexes with age (up to 8 weeks at 25 °C), in mitochondria from adult flight muscles (Fig. S1a,b). We also examined whether raising the adult flies under mild thermal stress at 27 °C—which should accelerate the aging process—would alter the amount of CI assembled. This was accomplished by monitoring CI assembly after eclosure at 25 °C and within 1 h after shifting the flies to 27 °C, as well as 2 and 4 weeks after shifting to 27 °C. Similar to observations made at 25 °C, OXPHOS integrity was normal even after 4 weeks at 27 °C (Fig. S1a,b). These results were corroborated with in-gel CI, complex II (CII), and complex IV (CIV) activities at both 25 °C and 27 °C (Fig. S1c). Taken together, we conclude that the amount of CI assembled remains largely intact in mitochondria from 4- and 8-week aged flight muscles at 27 °C and 25 °C, respectively.

### The expression of dNDUFS3 in subcomplexes is down-regulated with age

Previous studies from our research group have revealed that the amount of CI assembled in *Drosophila* flight muscles as determined by BN-PAGE can sometimes appear normal although de novo biosynthesis of various CI subcomplexes may be impaired^16,28^. This is most likely due to the fact that once OXPHOS complexes are assembled in *Drosophila* flight muscles, they tend to be very stable to the point where very little change, if any, in the amount of OXPHOS complexes assembled can be detected by BN-PAGE, even when de novo CI  biogenesis is hampered. We decided to exploit this property to ascertain how de novo CI biogenesis is altered in the aging muscle despite the relatively normal amounts of fully-assembled CI.

**Figure 1:**
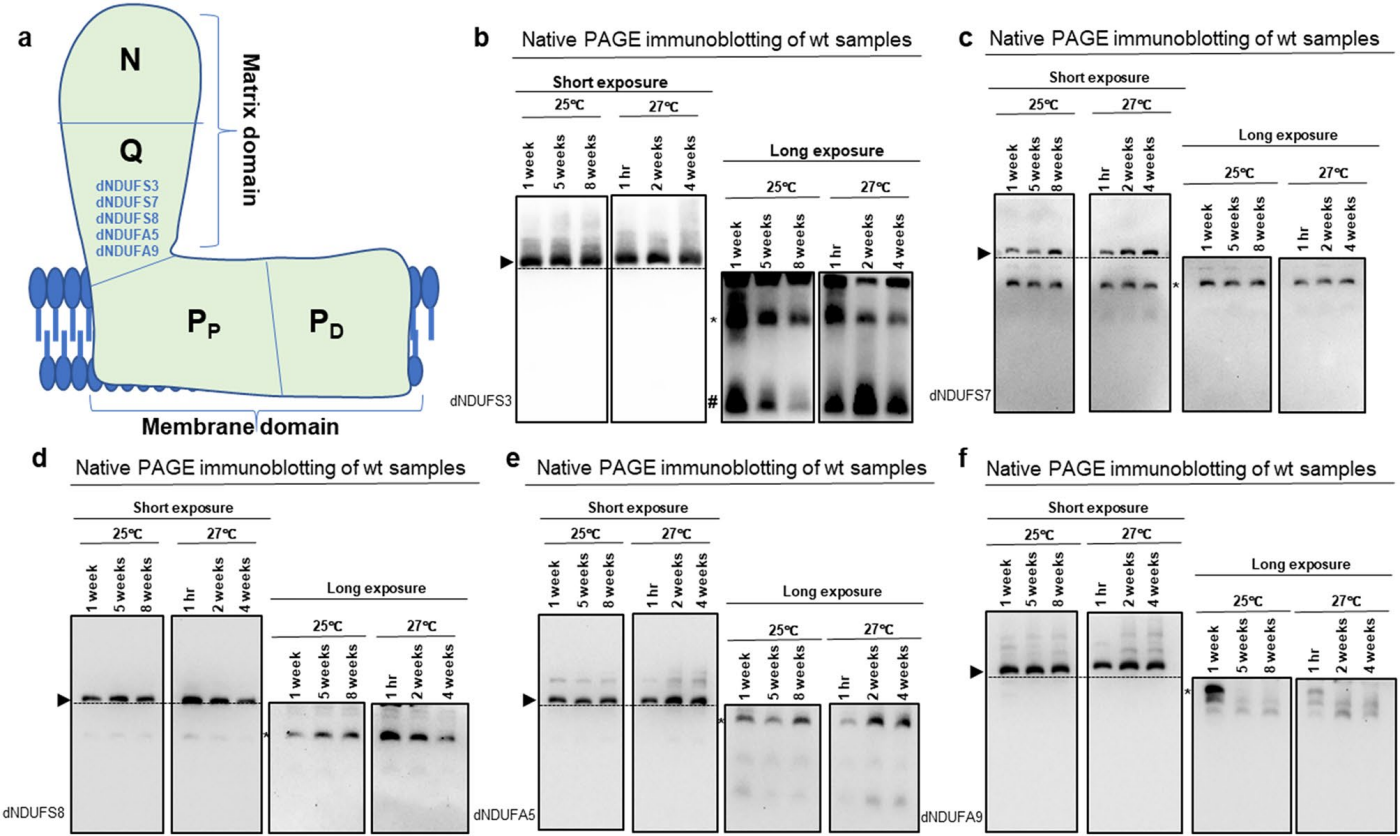
The expression of dNDUFS3 in subcomplexes is down-regulated with age. (**a**) A depiction of mitochondrial CI showing the matrix and membrane domains oriented almost orthogonally to each other, and the relative positions of the N-, Q-, P_P_- and P_D_-modules. A commercially available antibody that detects dNDUFS3 and antibodies we generated against dNDUFS7, dNDUFS8, dNDUFA5 and dNDUFA9 were used to examine the biogenesis of the Q-module. (**b**–**f**) Mitochondrial preparations from thoraces dissected from *Dmef2-Gal4/w*^*1118*^ (wt) flies raised at 25 °C or 27 °C for up to 8 weeks and 4 weeks, respectively, were analyzed by BN-PAGE, followed by immunoblotting with the antibodies indicated. The blots were imaged following a short exposure to detect the holoenzyme (fully-assembled CI) and supercomplexes, after which the region corresponding to the holoenzyme and supercomplexes was cut off, and the rest of the blot re-imaged after a longer exposure to detect the subcomplexes. The following antibodies were used: anti-NDUFS3 which detects dNDUFS3 (**b**), anti-dNDUFS7 (**c**), anti-dNDUFS8 (**d**), anti-dNDUFA5 (**e**) and anti-dNDUFA9 (**f**). The arrowheads mark the positions of the holoenzyme and the asterisks denote subcomplexes discussed in the text. Initiating and intermediary Q-module subcomplexes in panel (**b**) are denoted by # and *, respectively. We used the amount of holoenzyme detected by each antibody as a loading control for the subcomplexes. See Figures S2 and S3 for replicates and S7 for full-length gels and blots.

During CI biogenesis, subcomplexes consisting of a few CI subunits are first synthesized and eventually coalesce with other subcomplexes and CI subunits en route to forming the fully-assembled CI^12,14,15^. The three modules of CI are synthesized from specific subcomplexes (Figs. 1a and S2a). Using the same convention employed in our previous publications, for simplicity and clarity, we prefix all *Drosophila melanogaster* orthologs of CI subunits with the letter “d”; their exact CG numbers have been described previously^15,16,28,29^.

The Q-module consists of dNDUFS2, dNDUFS3, dNDUFS7, dNDUFS8, dNDUFA5 and dNDUFA9. Therefore, we monitored Q-module stability via Western blotting of blue native gels 1 week, 5 weeks, and 8 weeks after eclosure at 25 °C, with anti-NDUFS3 (which detects dNDUFS3), anti-dNDUFS7, anti-dNDUFS8, anti-dNDUFA5 and anti-dNDUFA9 antibodies (Figs. 1b–f and S2b–f). As the amount of the holoenzyme—fully-assembled CI devoid of supercomplexes—is not altered with age, we used the amount of the fully-assembled CI, shown in the short exposure panels, as loading controls for the subcomplexes which are shown in the long exposure panels. We observed an age-dependent decrease in the level of incorporation of dNDUFS3 in both initiating and intermediary subcomplexes of the Q-module at 25 °C (Figs. 1b, S2b and S3). A similar age-dependent decline in the incorporation of dNDUFS3 was observed in the intermediary Q-module subcomplex of samples isolated 2 and 4 weeks after incubation at 27 °C (Figs. 1b, S2b and S3). However, no reproducible pattern of expression was observed for dNDUFS7-containing subcomplexes at either 25 °C or 27 °C (Figs. 1c and S2c). The amount of dNDUFS8 that had incorporated into subcomplexes with age remained relatively uniform at 25 °C but was progressively reduced with age at 27 °C (Figs. 1d and S2d). Similar to dNDUFS7, no consistent pattern of expression was observed for dNDUFA5- or dNDUFA9-containing subcomplexes at either temperature (Figs. 1e,f, S2e,f). Hence, we conclude that while the pattern of expression of different Q-module subunits varies with age, one of the initiating subunits for Q-module synthesis, dNDUFS3, is down-regulated in CI subcomplexes during muscle aging.

### Stochastic expression of N-module subunits in subcomplexes with age

**Figure 2:**
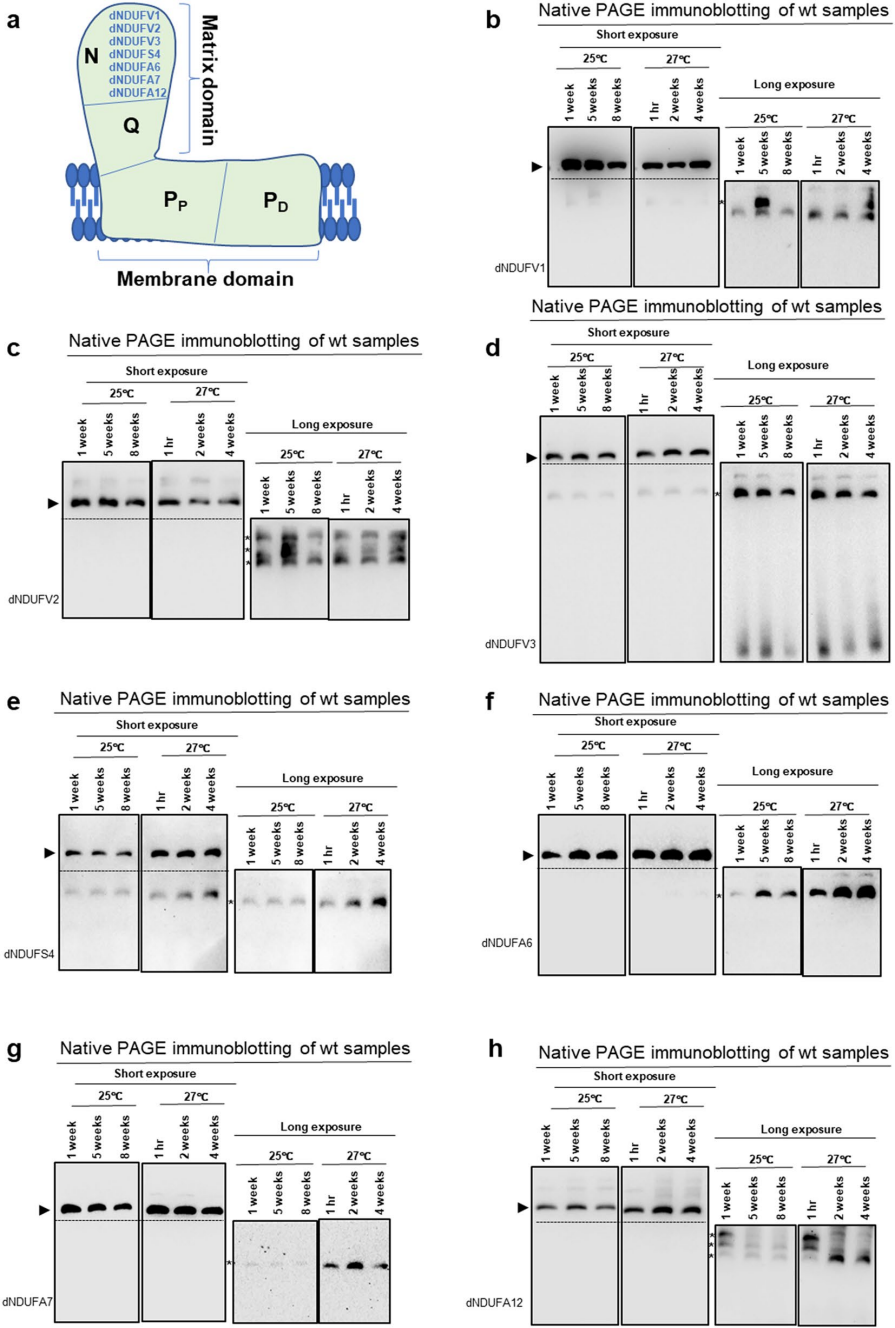
Stochastic expression of N-module subunits in subcomplexes with age. (**a**) An illustration of mitochondrial CI depicting the approximate positions of the N-, Q-, P_P_- and P_D_-modules. Biogenesis of the N-module was tracked by immunoblotting with antibodies against dNDUFV1, dNDUFV2, dNDUFV3, dNDUFS4, dNDUFA6, dNDUFA7 and dNDUFA12. (**b**–**h**) Mitochondrial preparations isolated from thoraces of wt flies at the time points shown and raised at the temperatures indicated were analyzed by BN-PAGE, followed by Western blotting with the antibodies indicated. The antibodies used were anti-dNDUFV1 (**b**), anti-dNDUFV2 (**c**), anti-dNDUFV3 (**d**), anti-dNDUFS4 (**e**), anti-dNDUFA6 (**f**), anti-dNDUFA7 (**g**), and anti-dNDUFA12 (**h**). The arrowheads mark the positions of the holoenzyme which was used as a loading control for the subcomplexes. The asterisks denote subcomplexes discussed in the text. See Fig. S4 for replicates and S7 for full-length gels and blots.

Next, we turned our attention to checking how the integrity of the N-module changes with age. N-module integrity was examined by performing Western blots with antibodies that detect dNDUFV1, dNDUFV2, dNDUFV3, dNDUFS4, dNDUFA6, dNDUFA7 and dNDUFA12 (Figs. 2a and S4a). The stabilization or incorporation of both dNDUFV1 and dNDUFV2 was stochastic, as it appeared to be robustly upregulated at various time points, but with no obvious consistency between the replicates at 25 °C or 27 °C (Figs. 2b,c, S4b,c). The level of expression of dNDUFV3 did not show a consistent variation at either 25 °C or 27 °C (Figs. 2d and S4d). The amount of dNDUFS4 in subcomplexes appeared stable at 25 °C but was slightly upregulated at 27 °C (Figs. 2e and S4e). Finally, the extent of incorporation or stability of three additional N-module subunits—dNDUFA6, dNDUFA7 and dNDUFA12—also showed oscillations at various time points at 25 °C and 27 °C (Figs. 2f–h and S4f–h). Hence, we conclude that subcomplexes containing N-module subunits have a stochastic pattern of expression during normal aging at 25 °C or accelerated aging induced by thermal stress at 27 °C.

### A variable wave of up-regulation of several P_P_-module subcomplexes occurs during muscle aging

**Figure 3:**
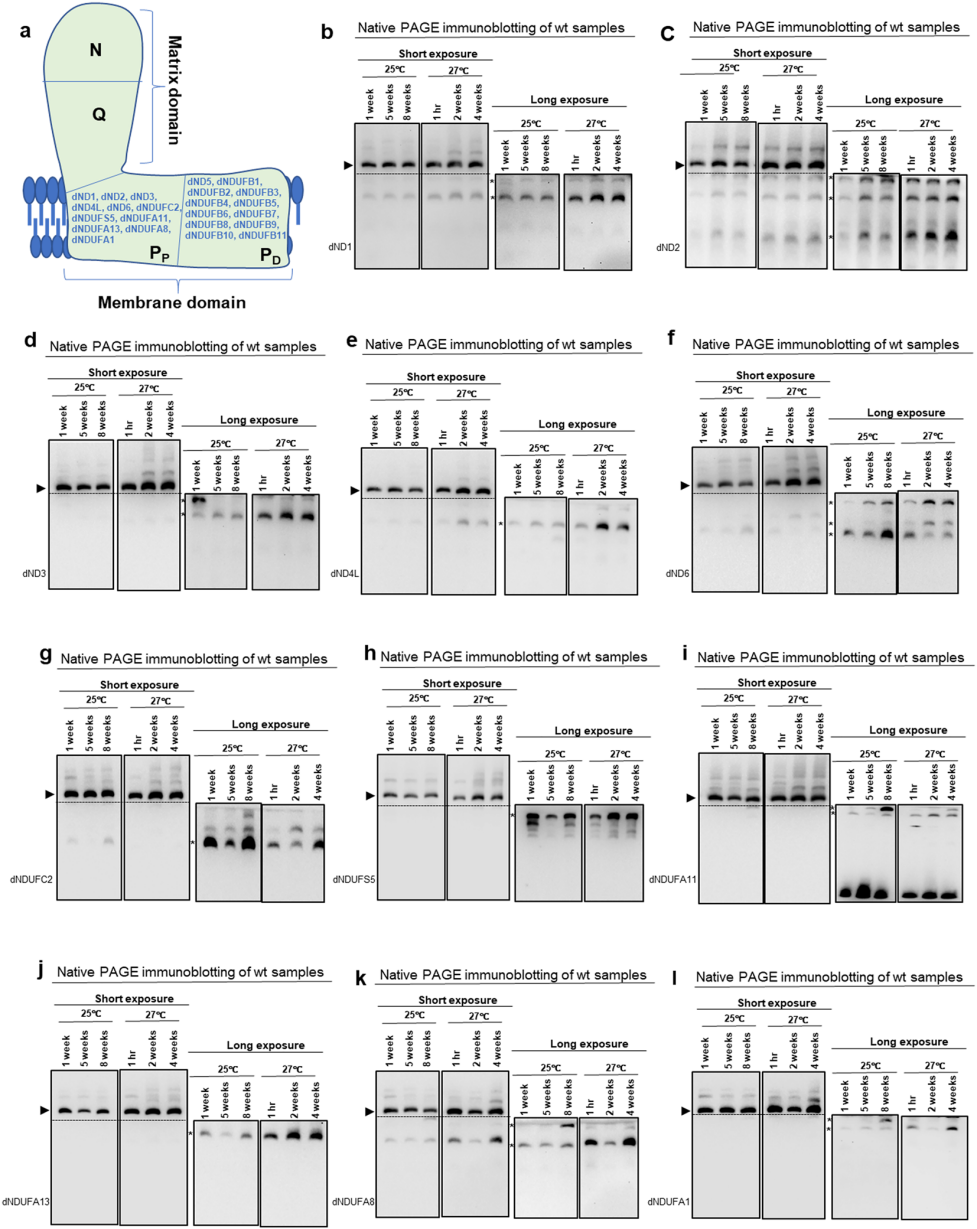
A variable wave of up-regulation of several P_P_-module subcomplexes occurs during muscle aging. (**a**) A diagram of mitochondrial CI showing the antibodies used to monitor the synthesis of the P_P_-and P_D_- modules. (**b**–**l**) Mitochondrial preparations obtained from thoraces of wt flies at the time points and temperatures indicated were analyzed by BN-PAGE, followed by Western blotting. The antibodies used were anti-dND1 (**b**), anti-dND2 (**c**), anti-dND3 (**d**), anti-dND4L (**e**), anti-dND6 (**f**), anti-dNDUFC2 (**g**), anti-dNDUFS5 (**h**), anti-dNDUFA11 (**i**), anti-dNDUFA13 (**j**), anti-dNDUFA8 (**k**), and anti-dNDUFA1 (**l**). See Fig. S5 for replicates and S7 for full-length gels and blots.

The membrane domain can be subdivided into a proximal P_P_-module and a distal P_D_-module, corresponding to regions of the membrane domain closest to, and more distal from the region where the matrix and membrane domains adjoin, respectively (Figs. 3a and S5a) [reviewed in^11–13^]. The seven mtDNA-encoded CI subunits—dND1, dND2, dND3 dND4, dND4L, dND5 and dND6—are localized to the membrane domain. P_P_-module mtDNA-encoded CI subunits are dND1, dND2, dND3 dND4L and dND6. To determine how the integrity of the P_P_-module varies with age, we first examined the extent of incorporation of the mtDNA-encoded subunits of the P_P_-module. In general, subcomplexes containing the P_P_-module mtDNA-encoded CI subunits vacillated between a slight increase to remaining largely unchanged at 27 °C with age; however, these subcomplexes remained largely stable at 25 °C (Figs. 3b–f and S5b–f).

We also examined the stability of some subcomplexes containing nuclear-encoded P_P_-module subunits such as dNDUFC2, dNDUFS5, dNDUFA11, dNDUFA13, dNDUFA8 and dNDUFA1. The amounts of subcomplexes containing dNDUFC2, dNDUFS5, dNDUFA11 and dNDUFA13 were variable at midlife at 25 °C (4 weeks); nevertheless, by 8 weeks at 25 °C some subcomplexes containing these 4 subunits were generally elevated relative to what was observed at midlife (Figs. 3g–j and S5g–j). Similarly, the amount of dNDUFC2-containing subcomplexes was reduced at 2 weeks at 27 °C but increased at 4 weeks (Figs. 3g and S5g). However, we detected subcomplexes containing dNDUFS5, dNDUFA11 and dNDUFA13 that were increased at both 2 and 4 weeks at 27 °C (Figs. 3h–j and S5h–j). Additionally, we detected dNDUFA8- and dNDUFA1-containing subcomplexes that were also up-regulated after 8 weeks at 25 °C and 4 weeks at 27 °C, respectively (Figs. 3k,l and S5k,l). When considered as a whole, we conclude that a variable wave of upregulation of several P_P_-module subcomplexes occurs during muscle aging.

### Multiple P_D_-module subcomplexes accumulate during muscle aging

**Figure 4:**
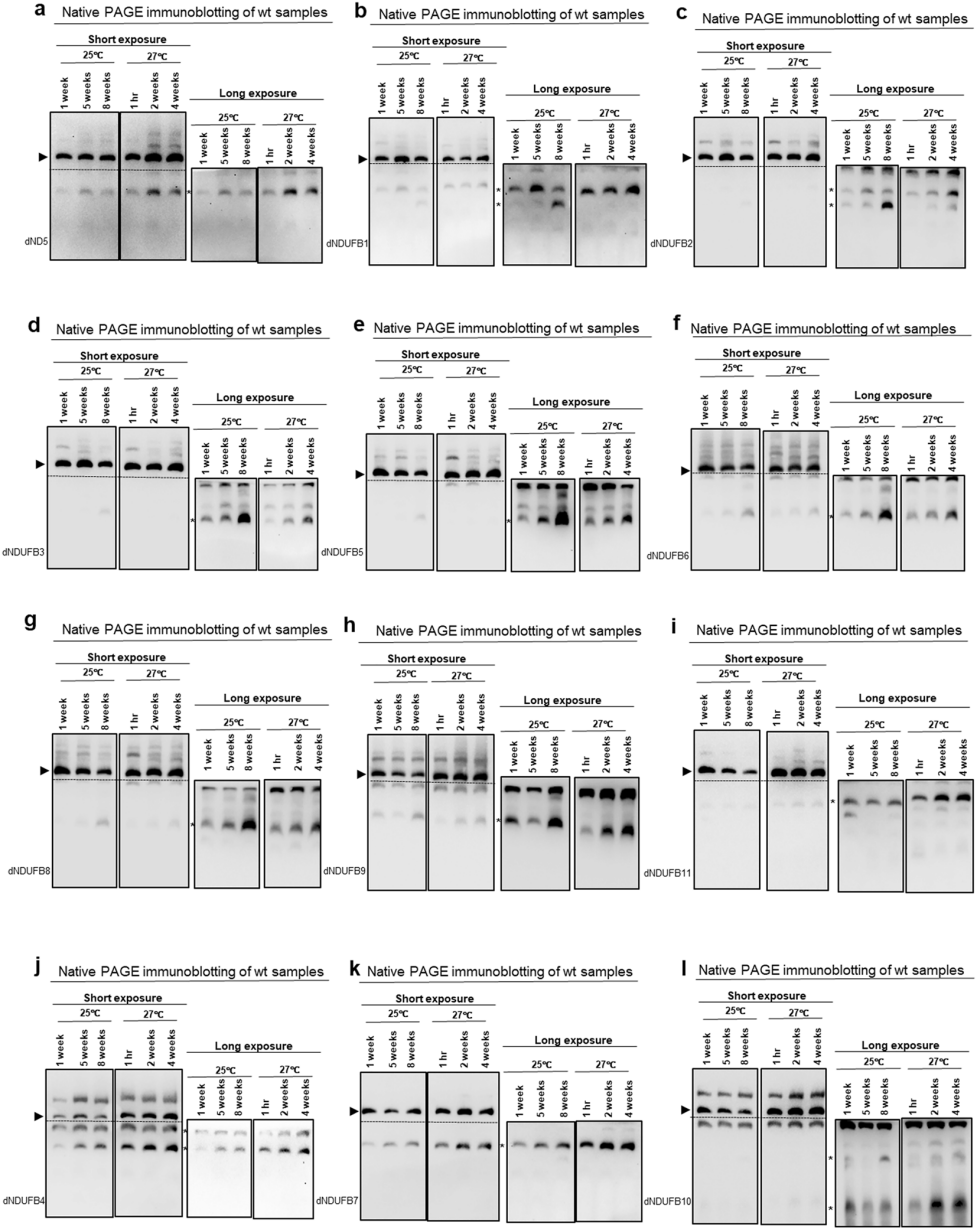
Multiple P_D_-module subcomplexes accumulate during muscle aging. (**a**–**l**) Mitochondrial preparations from thoraces of wt flies at the time points and temperatures indicated were analyzed by BN-PAGE, followed by immunoblotting. The antibodies used for the immunoblots were anti-dND5 (**a**), anti-dNDUFB1 (**b**), anti-dNDUFB2 (**c**), anti-dNDUFB3 (**d**), anti-dNDUFB5 (**e**), anti-dNDUFB6 (**f**), anti-dNDUFB8 (**g**), anti-dNDUFB9 (**h**), anti-dNDUFB11 (**i**), anti-dNDUFB4 (**j**), anti-dNDUFB7 (**k**) and anti-dNDUFB10 (**l**). See Fig. S6 for replicates and S7 for full-length gels and blots.

Lastly, we examined the effect of aging on subcomplexes containing P_D_-module subunits (Fig. 3a). Immunoblotting revealed that some P_D_-module subcomplexes containing dND5, dNDUFB1, dNDUFB2, dNDUFB3, dNDUFB5, dNDUFB6, dNDUFB8 and dNDUFB9 accumulated with age at both 25 °C and 27 °C (Figs. 4a–h and S6a–h). We also observed that dNDUFB5-, dNDUFB6- and dNDUFB8-containing subcomplexes were particularly up-regulated at 8 weeks and 4 weeks at 25 °C and 27 °C, respectively (Figs. 4e–g and S6e–g). Further, dNDUFB11-containing subcomplexes accumulated with age at 27 °C; but this was not apparent when we analyzed subcomplexes at 25 °C (Figs. 4i and S6i). Subcomplexes containing dNDUFB4, dNDUFB7 and dNDUFB10, however, showed a variable pattern of expression, appearing to be increased at some time points but not in a consistent manner at both 25 °C and 27 °C (Figs. 4j–l and S6j–l). We note that the up-regulation of P_D_-module subcomplexes was not restricted to the nuclear-encoded subunits as a subcomplex containing dND5 also displayed patterns of upregulation at both 25 °C and 27 °C (Figs. 4a and S6a). On the whole, we conclude that multiple P_D_-module subcomplexes accumulate during muscle aging.

## Discussion

We have examined how the integrity of the fully-assembled respiratory CI and the level of expression or stability of multiple CI subcomplexes in *Drosophila* flight muscles changes with increased lifespan. Surprisingly, our analyses of OXPHOS integrity by blue native PAGE and in-gel OXPHOS activity assays revealed that the integrity of the fully-assembled CI is not conspicuously altered with age. Instead, it is the de novo biogenesis of some CI subcomplexes that varies with age, such that flight muscle aging is associated with distinct changes in subcomplexes of different modules of the complex. The age-dependent alterations of different subunits within the same module was not always concordant. As a case in point, there was a decline in the amount of subcomplexes containing dNDUFS3, but the pattern of expression of other Q-module subunits in subcomplexes varied with age. In addition, while the age-dependent subcomplex profiles of many N-module subunits was random, membrane domain subcomplexes generally trended toward up-regulation with age. In this context, we hypothesize that if these observations are reproducible in humans, it will indicate that therapeutic attempts to improve CI function in aged individuals may not necessarily require increasing the expression of all CI subcomplexes but perhaps only the select few that deteriorate during aging.

Interestingly, we also uncovered what appeared to be variable waves of compensatory up-regulation of some N-module subcomplexes. We postulate that this is part of an adaptive mitochondrial stress response triggered, conceivably, as a result of increased ROS formation. RNAi-mediated knockdown of the *C. elegans* ortholog of NDUFS3, nuo-2, increases lifespan, possibly due to the induction of compensatory mitochondrial stress responses^30^. Additionally, as dNDUFS3 is one of the subunits that houses Fe–S clusters, degeneration of dNDUFS3 in subcomplexes would likely expose the highly redox-sensitive Fe–S clusters to ROS formation, which in turn, could also cause the activation of ROS-dependent adaptive mitochondrial stress signaling cascades. Undoubtedly, these periodic cycles of induction of N-module subcomplexes during muscle aging would have to be studied in more detail to identify the precise regulatory controls of this phenomenon, as the signaling pathways regulating such events could also be exploited in therapeutic settings to stimulate various aspects of CI biogenesis.

Given that dNDUFS3 is one of the critical subunits that is involved in the initiation of CI biogenesis, it is tempting to speculate on why its expression level in subcomplexes deteriorates so rapidly with age. Possible reasons include a hypersensitivity to ROS-induced protein modifications due to its close proximity to Fe–S clusters. But this cannot be the sole reason as the amounts of several Fe–S cluster-interacting core subunits in subcomplexes (such as dNDUFS7, dNDUFS8, dNDUFV1 and dNDUFV2) did not fall with age, at least not as rapidly as dNDUFS3. These findings further highlight the master regulatory role that dNDUFS3 has in stimulating CI biogenesis, and now, as we report, in the stalling of biogenesis as well.

A stoichiometric imbalance between nuclear- and mtDNA-encoded CI subunits, referred to as mitonuclear protein imbalance, activates the mitochondrial unfolded protein response (UPR^mt^)^31^. While we have not examined the level of expression of all nuclear- and mtDNA-encoded CI subunits to assess whether a mitonuclear imbalance exists at the protein translational level to trigger the UPR^mt^, the robust accumulation of several membrane domain subcomplexes could also produce a membrane domain:matrix domain protein imbalance that may also activate the UPR^mt^, and possibly other components of the mitochondrial protein quality control machinery. This would have beneficial effects on the whole mitochondrial proteome. In this regard, the dichotomy in subcomplex accumulation between the membrane and matrix domains could elicit a compensatory adaptive response.

We note that although the amount of fully-assembled CI remained relatively unchanged after 8 weeks at 25 °C, it is possible that some of the subcomplexes that accumulate could be re-initiated into the CI biosynthesis pathway at a later time point to replenish degenerating modules or the fully-assembled CI. In this regard, it has been shown in a mammalian cell line that a CI salvage pathway that turns over the N-module at a higher rate than other parts of the complex exists^17^. This ensures CI homeostasis at a lower bioenergetic cost than if the whole complex had to be degraded and resynthesized. However, we chose not to analyze the period beyond 8 weeks as it would be difficult to examine the impact of other aspects of aging on the CI assembly process. We also note that the apparent stability of the fully-assembled CI could be a reflection of a steady state where individual oxidatively damaged CI subunits are replaced without requiring a replacement of the whole complex as has been described previously^32^.

To summarize, we have characterized how the CI subcomplex profile changes with age at 25 °C and 27 °C. In general, we find that the deterioration of CI biogenesis observed with increasing age at 25 °C is accelerated at 27 °C. We also find that the rate of decay of subcomplexes is uneven, raising the possibility that forced expression of a few CI subunits such as dNDUFS3, may be all that is necessary to re-initiate the whole CI biogenesis pathway in mid- to late-adult life. We further observed that while many membrane domain subcomplexes accumulate with age, the fate of matrix domain subcomplexes with age was less predictable. Although we have not explored how CIAFs are altered in subcomplexes with age, our findings hint at the possibility that overexpression of a few CIAFs may be able to restore a deficient CI biogenesis pathway during muscle aging.

## Methods

### *Drosophila* stocks and husbandry

*Drosophila* strains were reared in vials containing agar, yeast, molasses and cornmeal medium supplemented with propionic acid and methylparaben in humidified environmental chambers (Forma environmental chambers) on a 12-h:12-h dark:light cycle. Genetic crosses were set up between female flies of the genotype, *y w; Dmef2-Gal4* (Ranganayakulu et al.,1996)^33^, and *w*^*1118*^ males at 25 °C. After the flies eclosed, they were maintained at 25 °C or 27 °C as explained below.

To examine the effect of normal aging on CI biogenesis, flies were aged at 25 °C for 1, 5 and 8 weeks prior to dissection of thoraces and subsequent mitochondria purification. To examine the effect of accelerated aging due to mild thermal stress on CI biogenesis, newly eclosed flies at 25 °C were immediately moved to 27 °C for up to an hour as the first time point used in our blue native immunoblots, and then 2 and 4 weeks prior to mitochondrial isolation from thoraces.

### Mitochondria isolation

Mitochondrial purification was performed essentially as described previously^28,34^. Thoraces were quickly dissected and gently crushed with a dounce homogenizer (10 strokes) in 500 μl of a prechilled mitochondrial isolation buffer consisting of 250 mM sucrose, 0.15 mM MgCl_2_, 10 mM tris–HCl, pH 7.4, supplemented with Halt Protease inhibitors (Pierce). Tissue homogenates were centrifuged twice at 500*g* for 5 min at 4 °C to eliminate the cuticle and other insoluble material. Afterwards, the supernatant was recovered and centrifuged at 5000*g* for 5 min at 4 °C to obtain the mitochondria-enriched pellet, which was washed twice in the mitochondrial isolation buffer and preserved at − 80 °C until further processing.

### Blue native polyacrylamide gel electrophoresis (BN-PAGE)

BN-PAGE was performed using NativePAGE gels (Life Technologies) and following the manufacturer’s protocol as described previously^28,35^.

### Silver staining

Silver staining of blue native gels was performed with a SilverXpress staining kit (Life Technologies) as described previously^28,35^, following the manufacturer’s instructions.

### In-gel complex I, II and IV activity

In-gel OXPHOS activities were performed as described previously^28,35^.

In-gel complex I activity was assessed by incubating the native gels in 0.1 mg/ml NADH, 2.5 mg/ml nitrotetrazolium blue chloride (NTB), 5 mM tris–HCl (pH 7.4) at 25 °C.

In-gel complex II activity was assessed by incubating the native gels in 20 mM sodium succinate, 0.2 mM phenazine methosulfate, 2.5 mg/ml NTB, 5 mM tris–HCl (pH 7.4) at 25 °C.

In-gel complex IV activity was assessed by incubating the native gels in 50 mM sodium phosphate (pH 7.2), 0.05% 3,3’- diaminobenzidine tetrahydrochloride (DAB), 50 μM horse heart cytochrome c at 25 °C.

### Generation of peptide polyclonal antibodies

Rabbit polyclonal antibodies recognizing various segments of specific target proteins in *Drosophila* were generated by Biomatik using the synthetic peptides listed below:

**Table:** 

Peptide antigen: Cys-RYWKESQDPKPTDFLSK	Target Protein: dNDUFA6 (CG7712)
Peptide antigen: ELLTNKGTHEPSFLSP-Cys	Target Protein: dNDUFC2 (CG12400)
Peptide antigen: NMDERFSRVMYQRDFRLTD-Cys	Target Protein: dNDUFA1 (CG34439)
Peptide antigen: Cys-PNSRKWSNTELGVPKDGF	Target Protein: dNDUFB2 (CG40472)
Peptide antigen: GEPYTVPHASTYKVES-Cys	Target Protein: dNDUFB3 (CG10320)
Peptide antigen: SNEEQEFIKRKHEAT-Cys	Target Protein: dNDUFB4 (CG12859)
Peptide antigen: THYMKPDVMPGPD-Cys	Target Protein: dNDUFB7 (CG5548)
Peptide antigen: Cys-EKQYGKPDPKDLGHH	Target Protein: dNDUFB9 (CG9306)
Peptide antigen: RRHGPVGSGMKEEAAH -Cys	Target Protein: dNDUFB10 (CG8844)
Peptide antigen: NTSPKKDETITAPTS-Cys	Target Protein: dNDUFB11 (CG6008)

### Western blotting

Western blotting was performed as previously described^16^. In brief, following the separation of protein complexes on 3–12% precast Bis–Tris Native PAGE gels (Life Technologies), the proteins were transferred to polyvinylidene difluoride (PVDF) membranes (Bio-Rad). Subsequently, the PVDF membrane was blocked in 5% (wt/vol) nonfat dry milk (NFDM) in tris-buffered saline (TBS) for 30 min and incubated in the appropriate primary antibody dissolved in 2% BSA and 0.1% Tween 20 in TBS (TBST) overnight at 4 °C. Following the overnight incubation, the blot was rinsed four times for 10 min each in 0.1% TBST, blocked for 30 min in 5% (wt/vol) NFDM in TBST, and incubated for 2 h at room temperature with the appropriate HRP-conjugated secondary antibody dissolved in 2% BSA and 0.1% TBST. Afterwards, samples were rinsed four times for 10 min each in 0.1% TBST. Immunoreactivity was detected by a SuperSignal West Pico PLUS Chemiluminescent kit (ThermoFisher) and analyzed by a ChemiDoc gel imaging system from Bio-Rad. The primary antibodies used were anti-NDUFS3 (Abcam, ab14711), anti-dNDUFS7^28^*,* anti-dNDUFS8^28^*,* anti-dNDUFA5^29^, anti-dNDUFA9^29^, anti-dNDUFV1^16^*,* anti-dNDUFV2^29^, anti-dNDUFV3^28^, anti-dNDUFS4^29^*,* anti-dNDUFA6 (generated during this study), anti-dNDUFA7^29^, anti-dNDUFA12^28^*,* anti-dNDUFS5^16^, anti-dNDUFC2 (this study), anti-dNDUFA1 (this study), anti-dNDUFA8^28^*,* anti-dNDUFA11^28^, anti-dNDUFA13^29^, anti-dND1^16^, anti-dND2^16^, anti-dND3^16^*,* anti-dND4L^16^, anti-dND5^16^*,* anti-dND6^16^*,* anti-dNDUFB1^29^, anti-dNDUFB2 (this study), anti-dNDUFB3 (this study), anti-dNDUFB4 (this study), anti-dNDUFB5^16^, anti-dNDUFB6^16^, anti-dNDUFB7 (this study), anti-dNDUFB8^16^, anti-dNDUFB9 (this study), anti-dNDUFB10 (this study), and anti-dNDUFB11 (this study). Secondary antibodies used were goat anti-rabbit horseradish peroxidase (PI31460 from Pierce) and goat anti-mouse horseradish peroxidase (PI31430 from Pierce).

## Supplementary Information

Supplementary Information.

## Data Availability

All data needed to evaluate the conclusions in the paper are present in the paper and/or the Supplementary Materials.
