# Supplementary material for: The membrane domain of respiratory complex I accumulates during muscle aging in Drosophila melanogaster
Source: Sci Rep. 2022 Dec 27;12:22433. doi: 10.1038/s41598-022-26414-5 (PMC9794728; doi:10.1038/s41598-022-26414-5)
Supplement: Supplementary file 1 — Supplementary Information. [file 41598_2022_26414_MOESM1_ESM.pdf]

Supplementary Information for

The membrane domain of respiratory complex I accumulates during muscle aging in *Drosophila melanogaster.*

Kaniz FB Hossain[1], Anjaneyulu Murari[1], Bibhuti Mishra[1], and Edward Owusu-Ansah[1*]

[1] Department of Physiology and Cellular Biophysics,

Columbia University Irving Medical Center,

New York, NY 10032, USA.

*Lead Contact and Correspondence: eo2364@cumc.columbia.edu

Figure S1: The effect of aging and thermal stress on mitochondria CI assembly

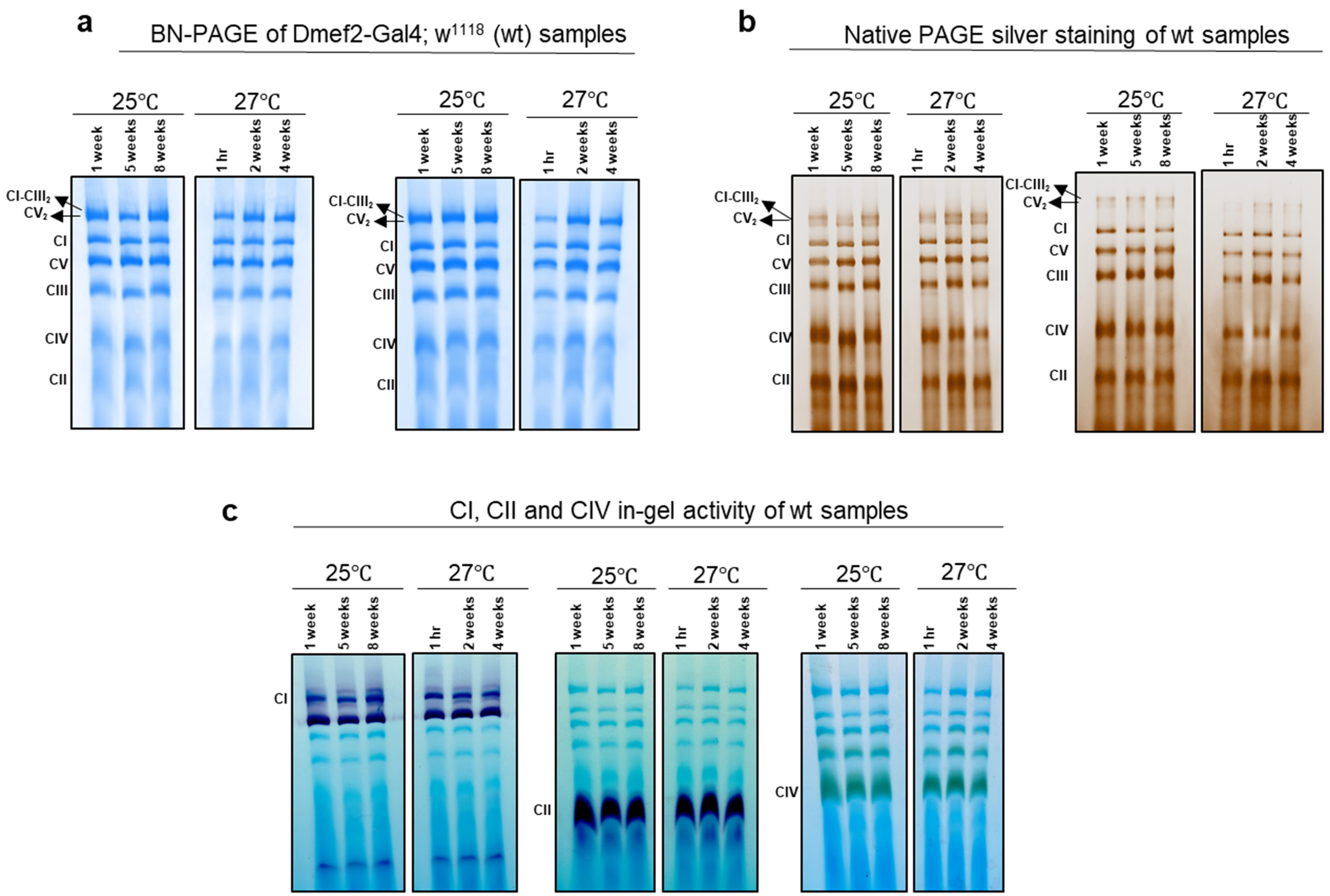

The amount of fully-assembled CI is not altered during muscle aging or mild thermal stress.

Figure S2: The expression of dNDUFS3 in subcomplexes is down-regulated with age

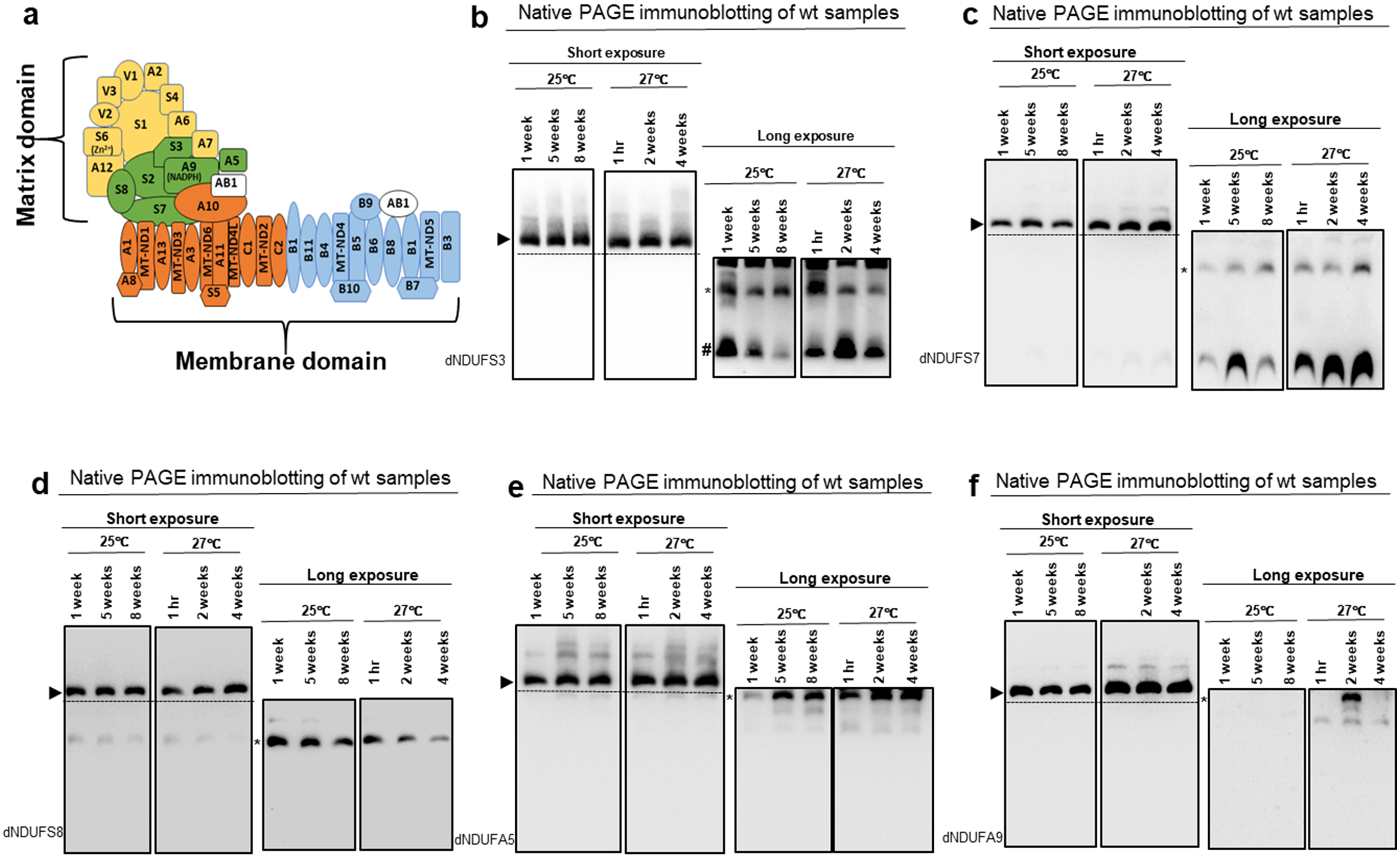

The figure legend is the same as Figure 1

Figure S3: The expression of dNDUFS3 in subcomplexes is down-regulated with age.

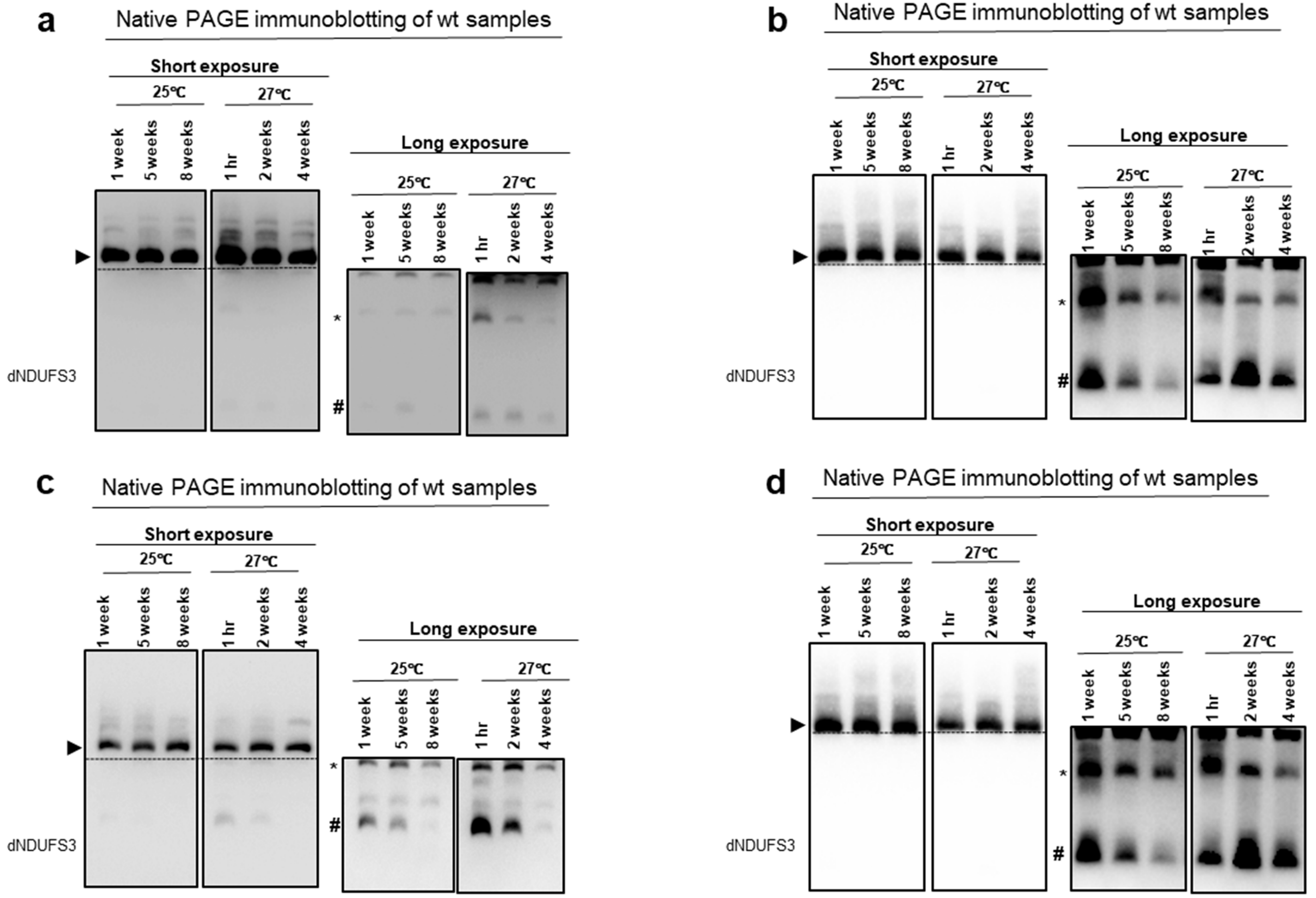

Additional replicates of data shown in Figure 1B

Figure S4: Stochastic expression of N-module subunits in subcomplexes with age

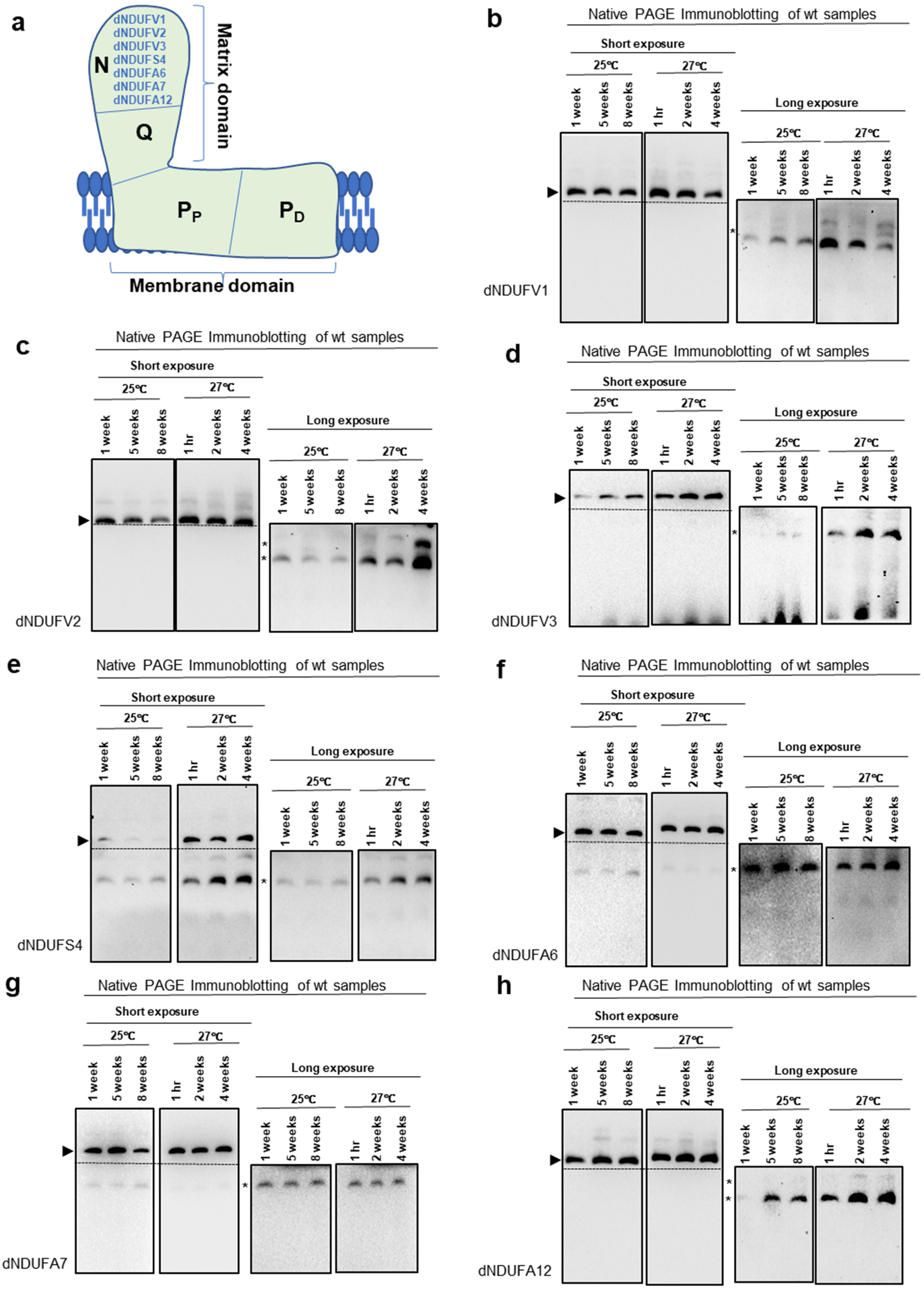

The figure legend is the same as Figure 2

Figure S5: P_P-module subcomplexes during muscle aging

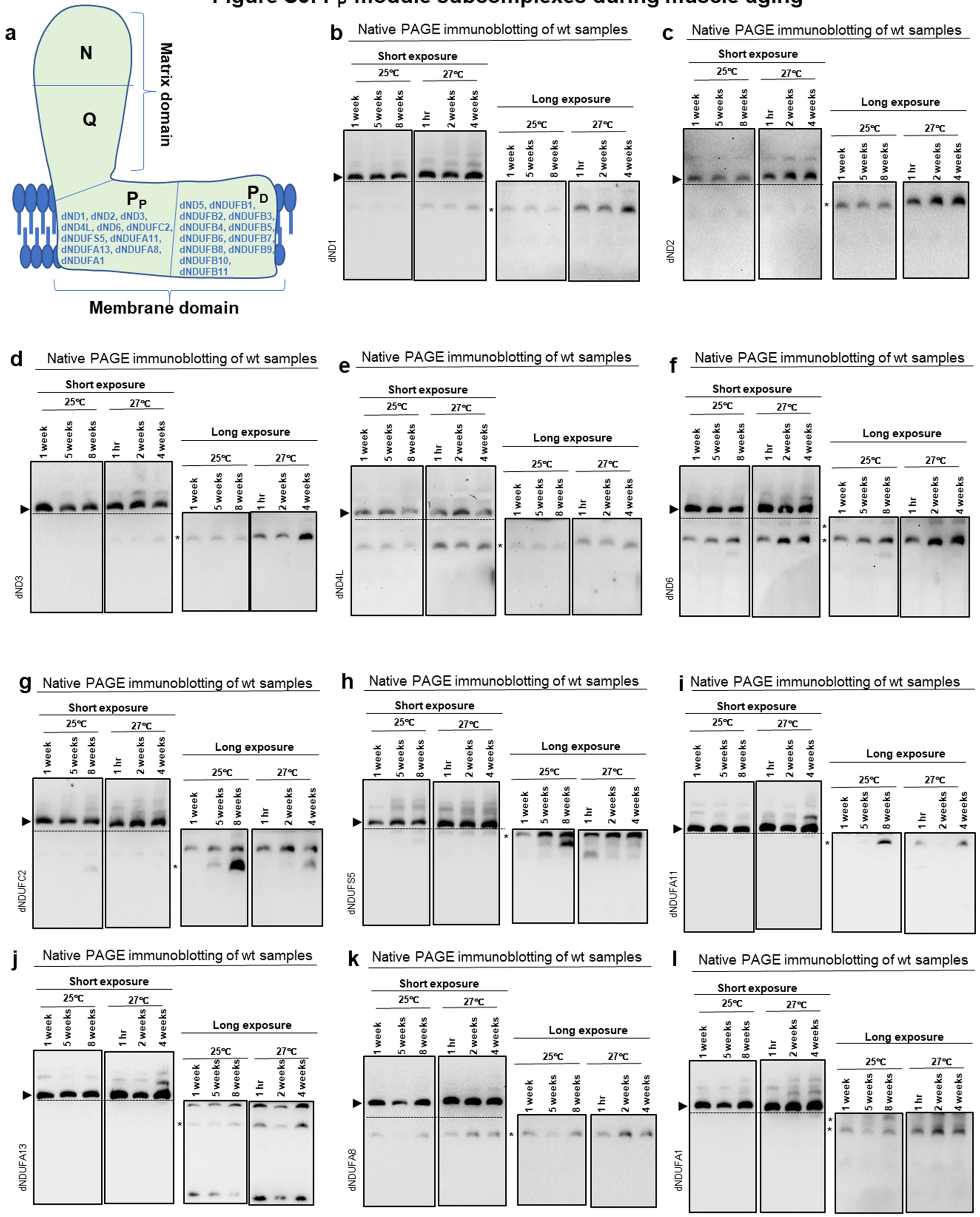

The figure legend is the same as Figure 3

Figure S6: P_D-module subcomplexes during muscle aging

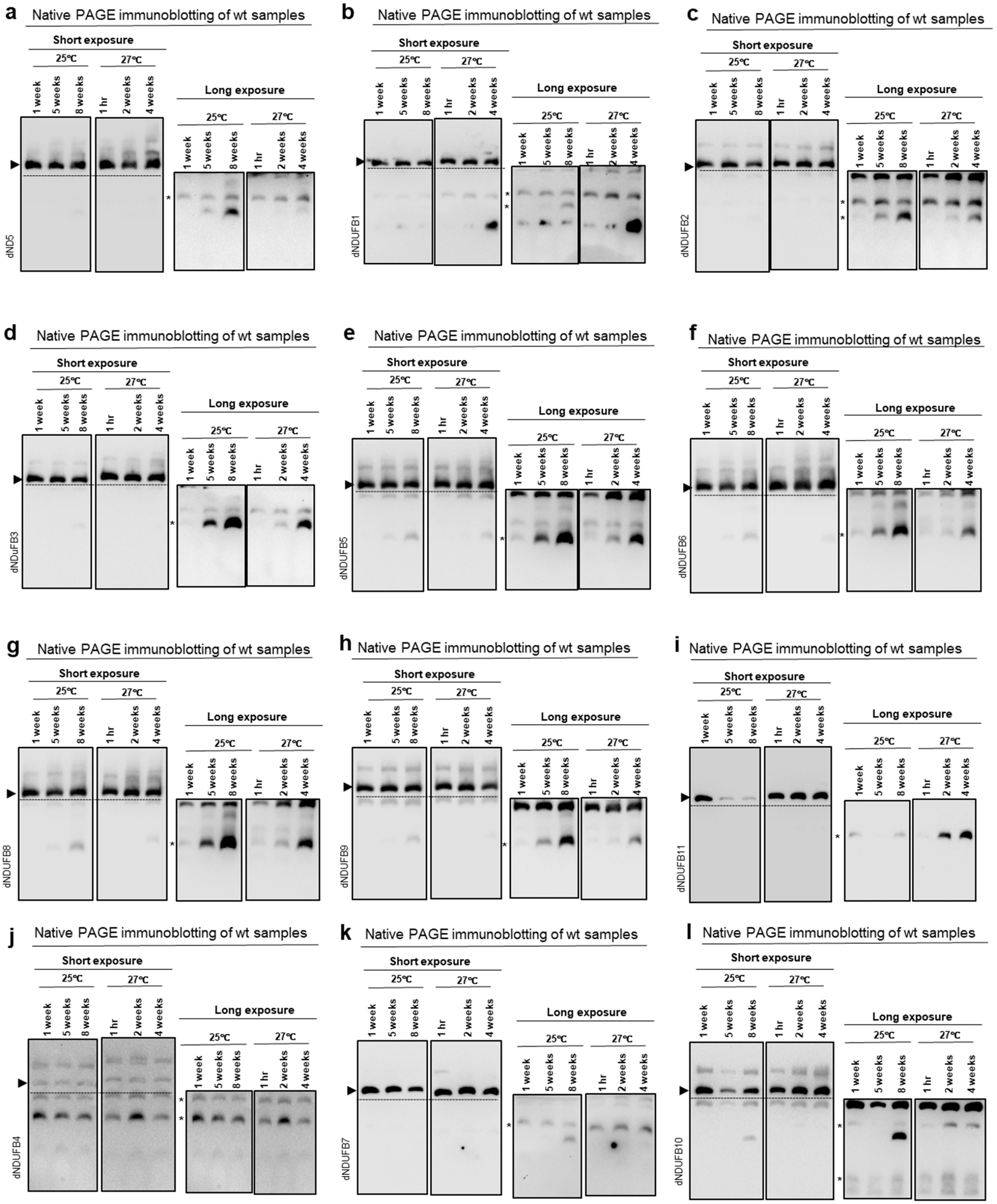

The figure legend is the same as Figure 4

Figure S7: All full-length blots and gels

Figure 1B

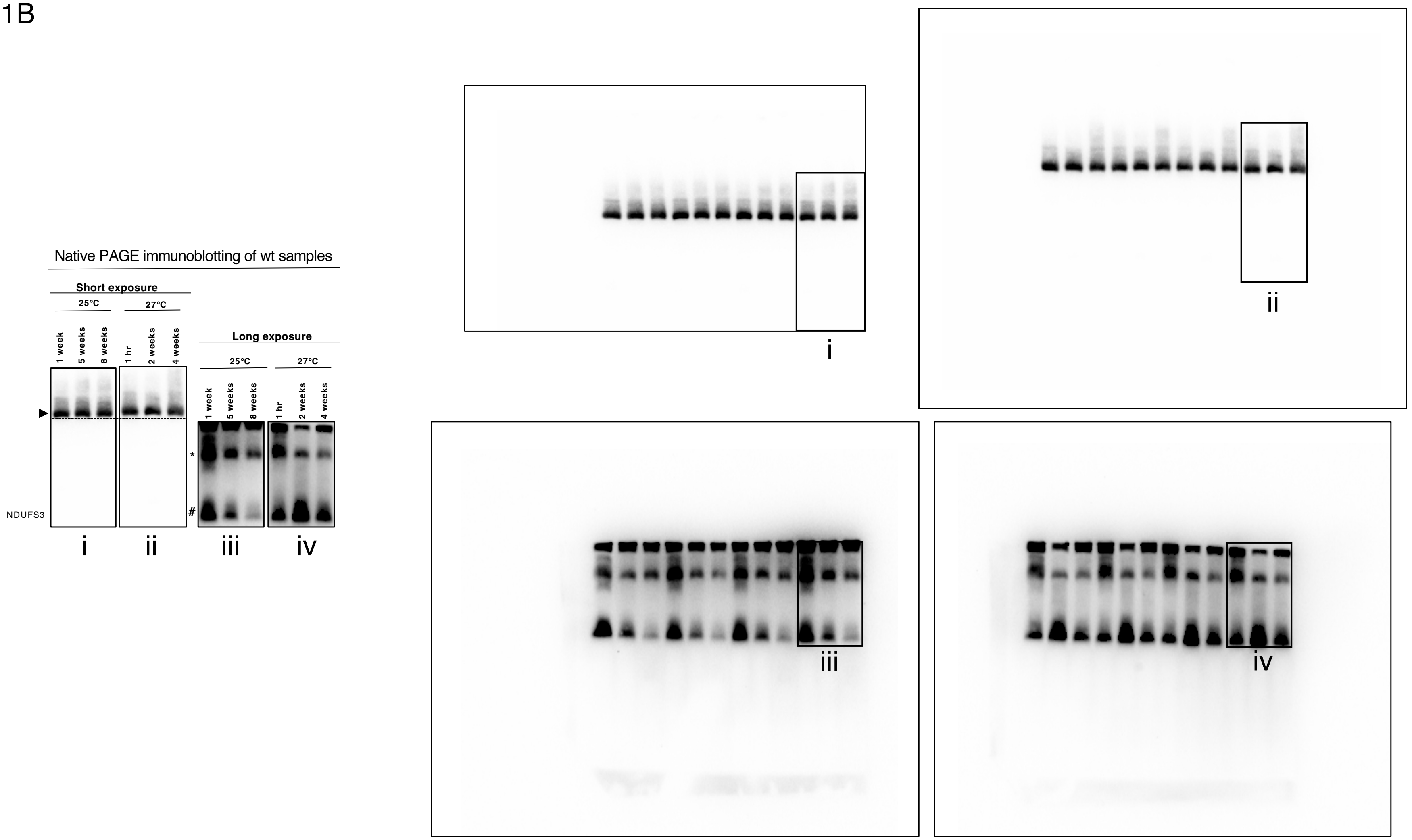
Native PAGE immunoblotting of wt samples
Short exposure
25°C
27°C
1 week
5 weeks
8 weeks
1 hr
2 weeks
4 weeks
Long exposure
25°C
27°C
1 week
5 weeks
8 weeks
1 hr
2 weeks
4 weeks
*
#
NDUFS3
i
ii
iii
iv
i
ii
iii
iv

1B additional exposures

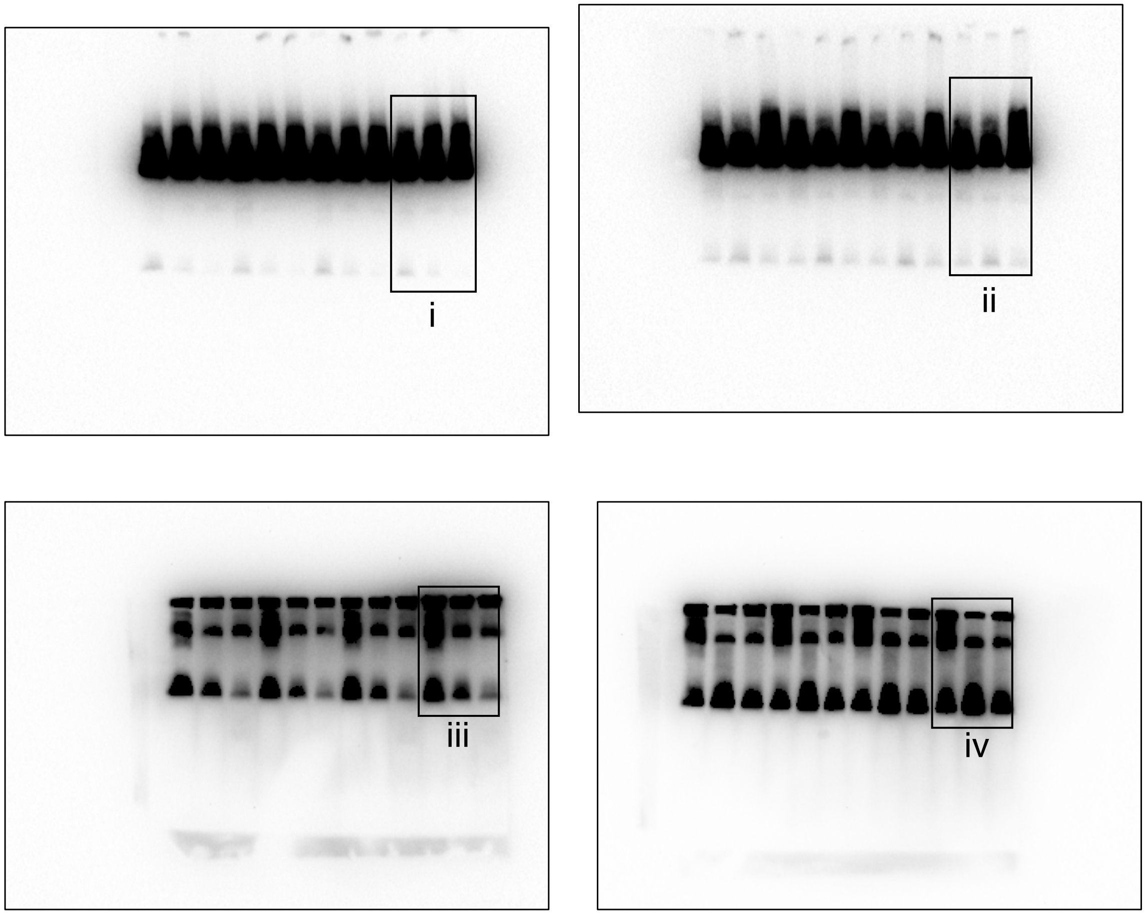

Figure 1C

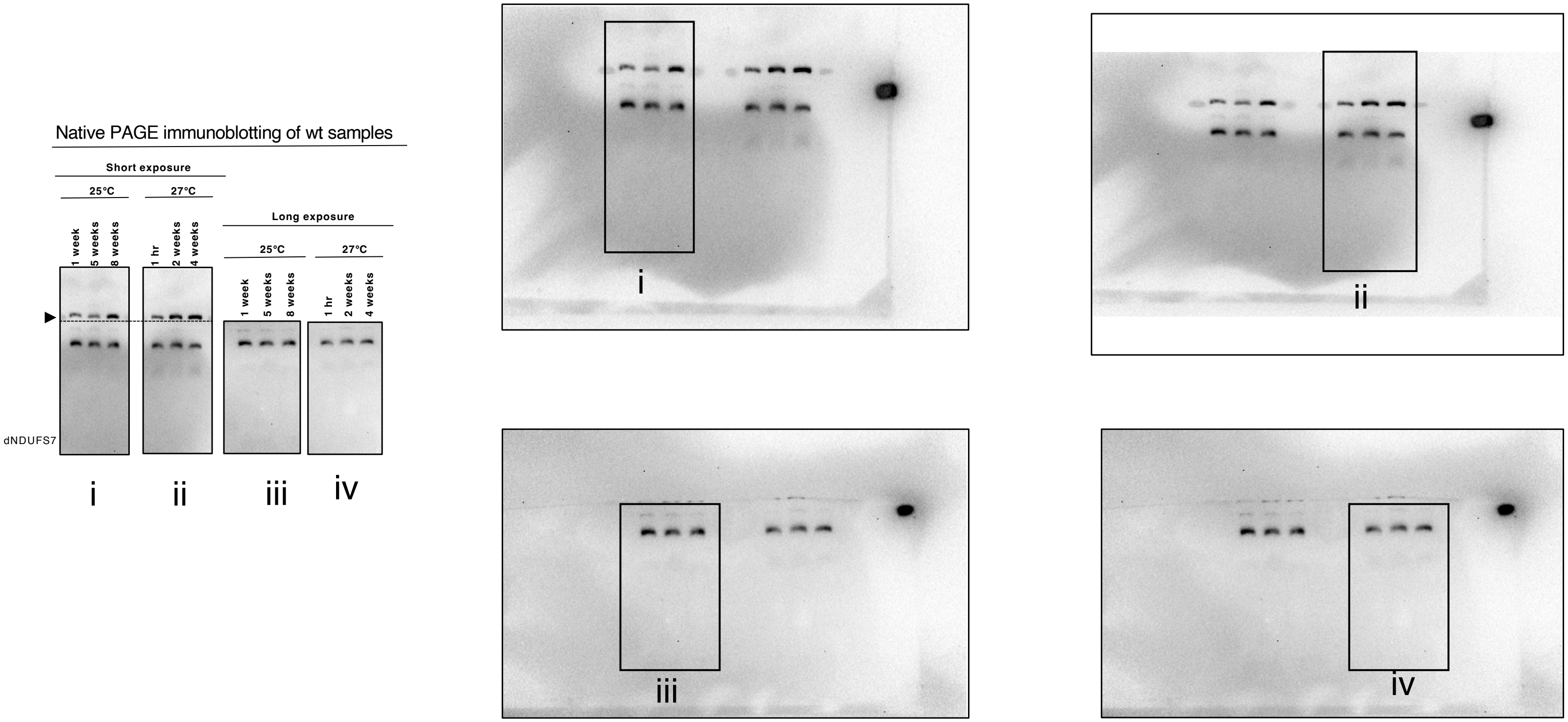

Native PAGE immunoblotting of wt samples
Short exposure
25°C
27°C
Long exposure
25°C
27°C
1 week
5 weeks
8 weeks
1 hr
2 weeks
4 weeks
1 week
5 weeks
8 weeks
1 hr
2 weeks
4 weeks
dNDUFS7
i
ii
iii
iv
i
ii
iii
iv

Figure 1D

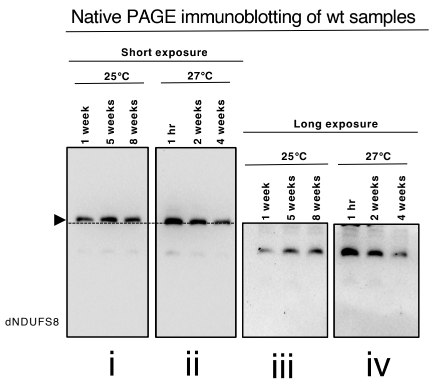

Native PAGE immunoblotting of wt samples
Short exposure
25°C
27°C
1 week
5 weeks
8 weeks
1 hr
2 weeks
4 weeks
Long exposure
25°C
27°C
1 week
5 weeks
8 weeks
1 hr
2 weeks
4 weeks
dNDUFS8
i
ii
iii
iv

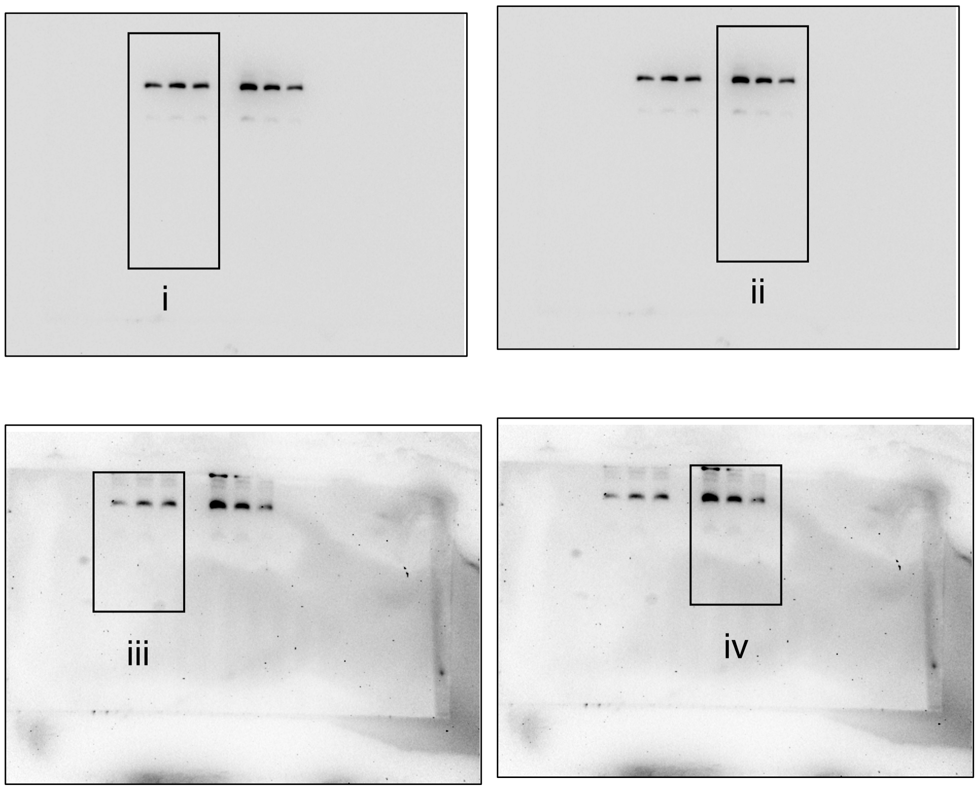

i
ii
iii
iv

Figure 1E

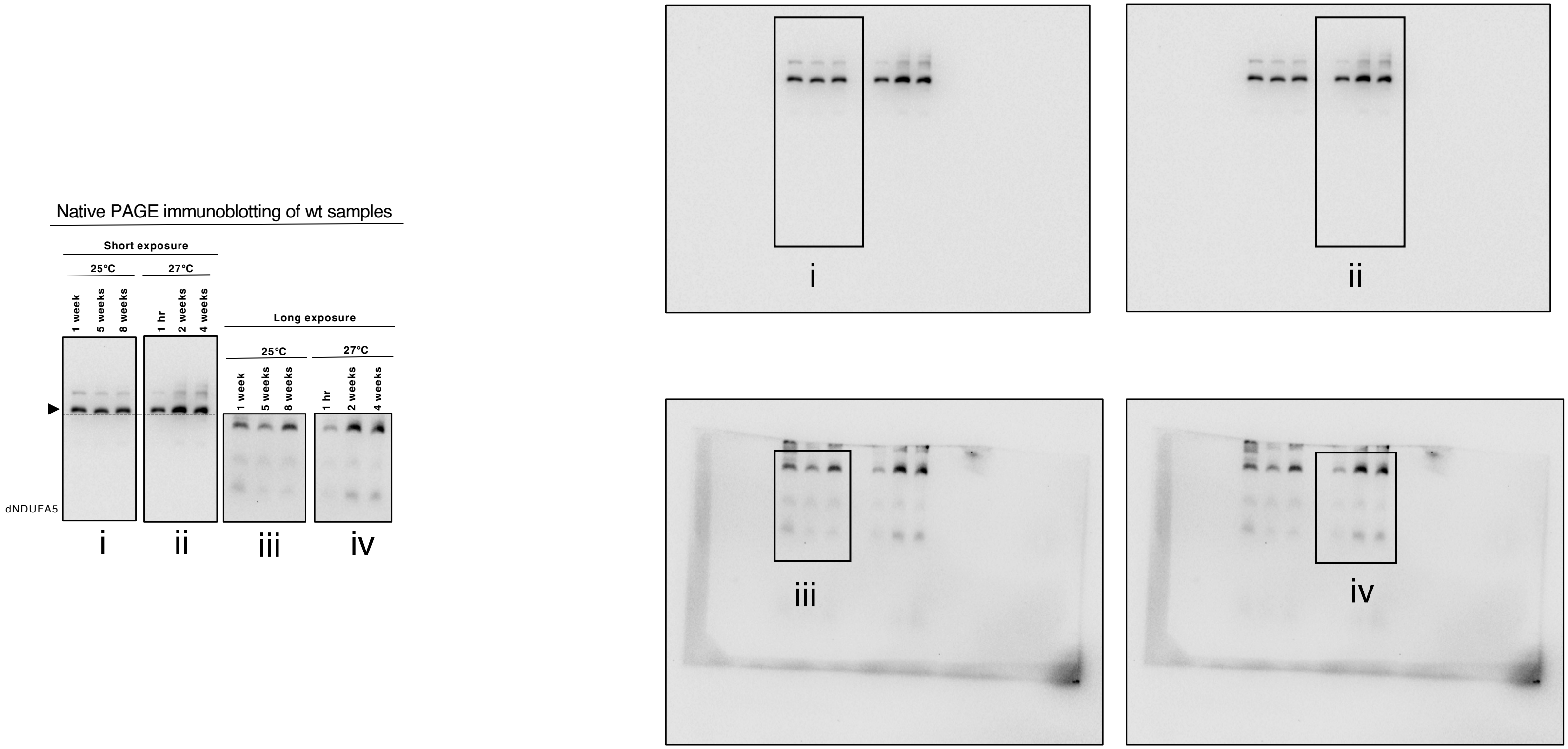
Native PAGE immunoblotting of wt samples
Short exposure
25°C
27°C
1 week
5 weeks
8 weeks
1 hr
2 weeks
4 weeks
Long exposure
25°C
27°C
1 week
5 weeks
8 weeks
1 hr
2 weeks
4 weeks
dNDUFA5
i
ii
iii
iv
i
ii
iii
iv

Figure 1F

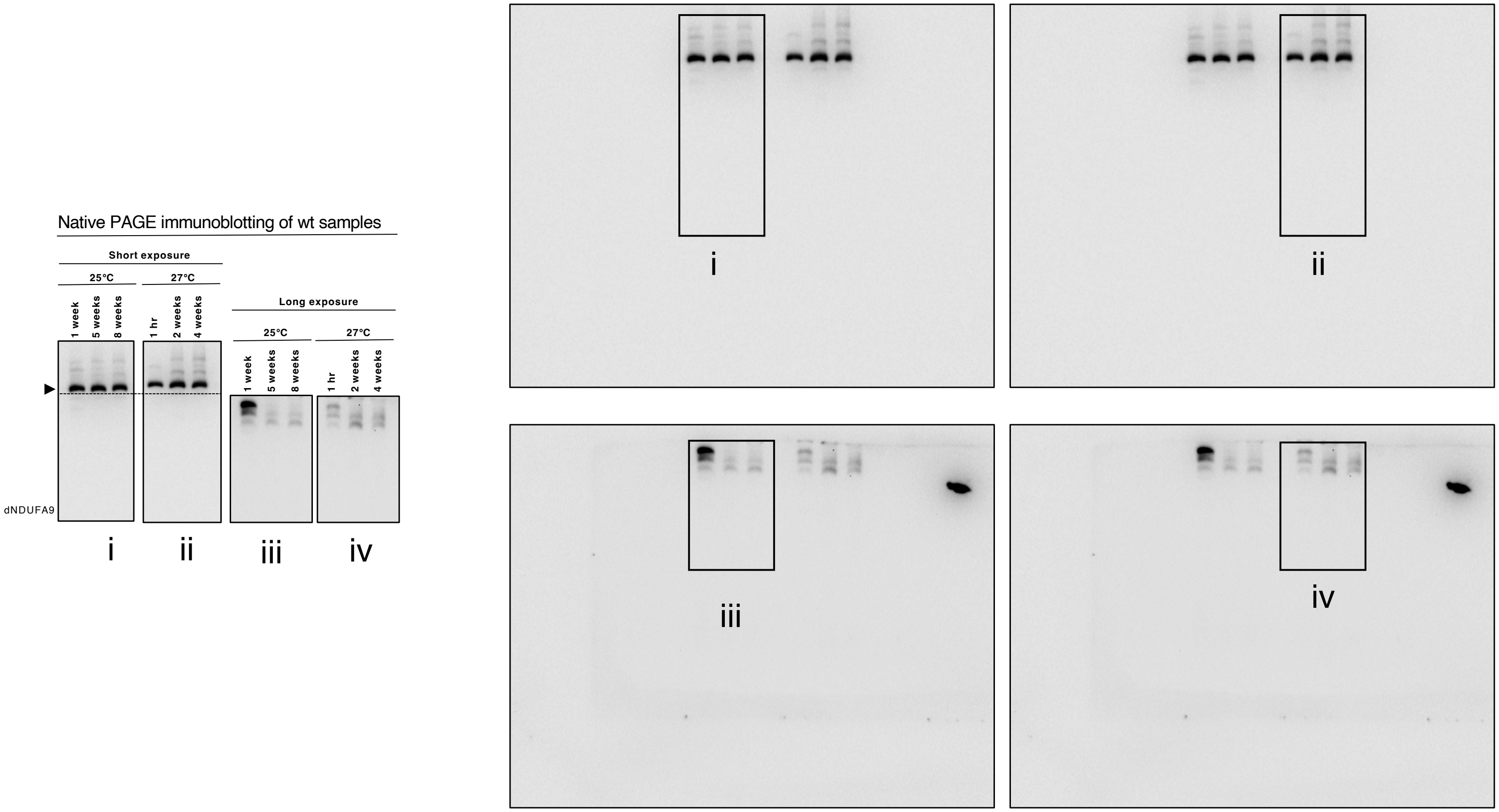
Native PAGE immunoblotting of wt samples
Short exposure
25°C
27°C
1 week
5 weeks
8 weeks
1 hr
2 weeks
4 weeks
Long exposure
25°C
27°C
1 week
5 weeks
8 weeks
1 hr
2 weeks
4 weeks
dNDUFA9
i
ii
iii
iv
i
ii
iii
iv

Figure 2B

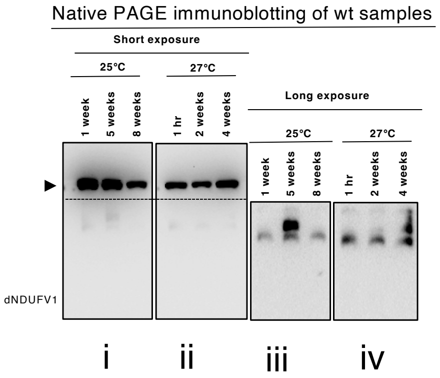

Native PAGE immunoblotting of wt samples
Short exposure
25°C
27°C
1 week
5 weeks
8 weeks
1 hr
2 weeks
4 weeks
Long exposure
25°C
27°C
1 week
5 weeks
8 weeks
1 hr
2 weeks
4 weeks
dNDUFV1
i
ii
iii
iv

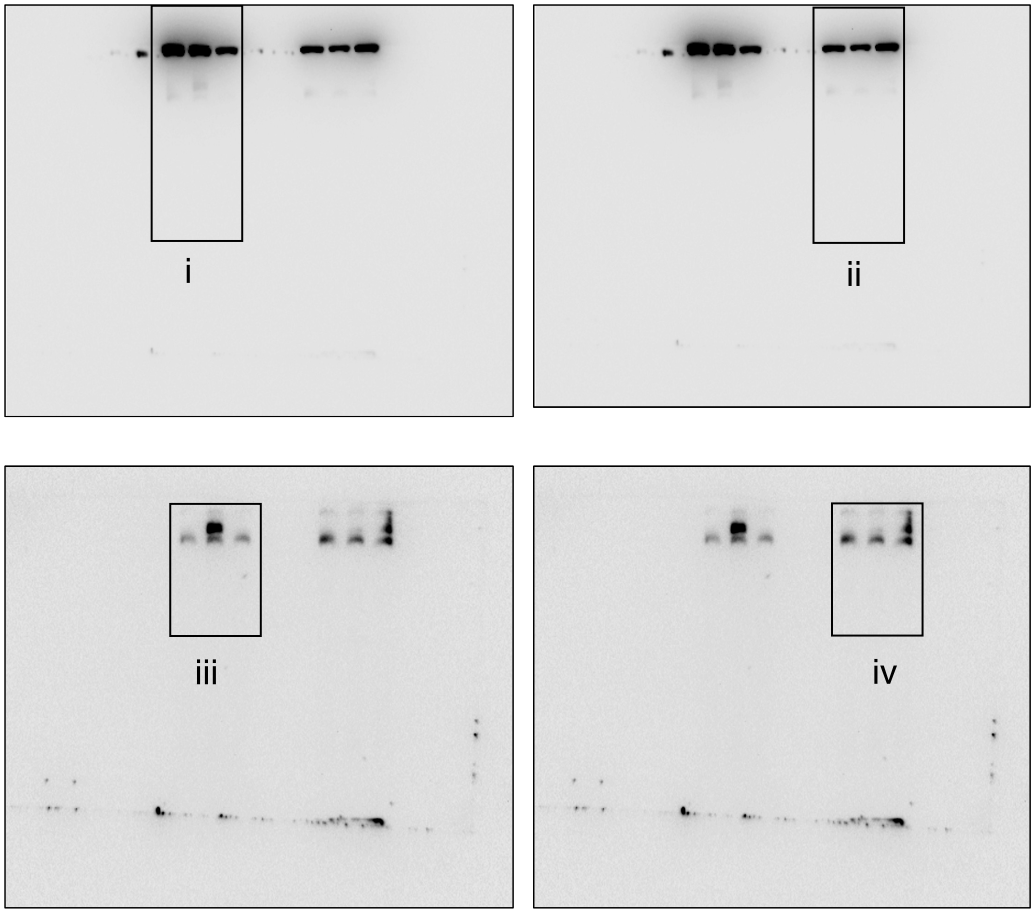

i
ii
iii
iv

Figure 2C

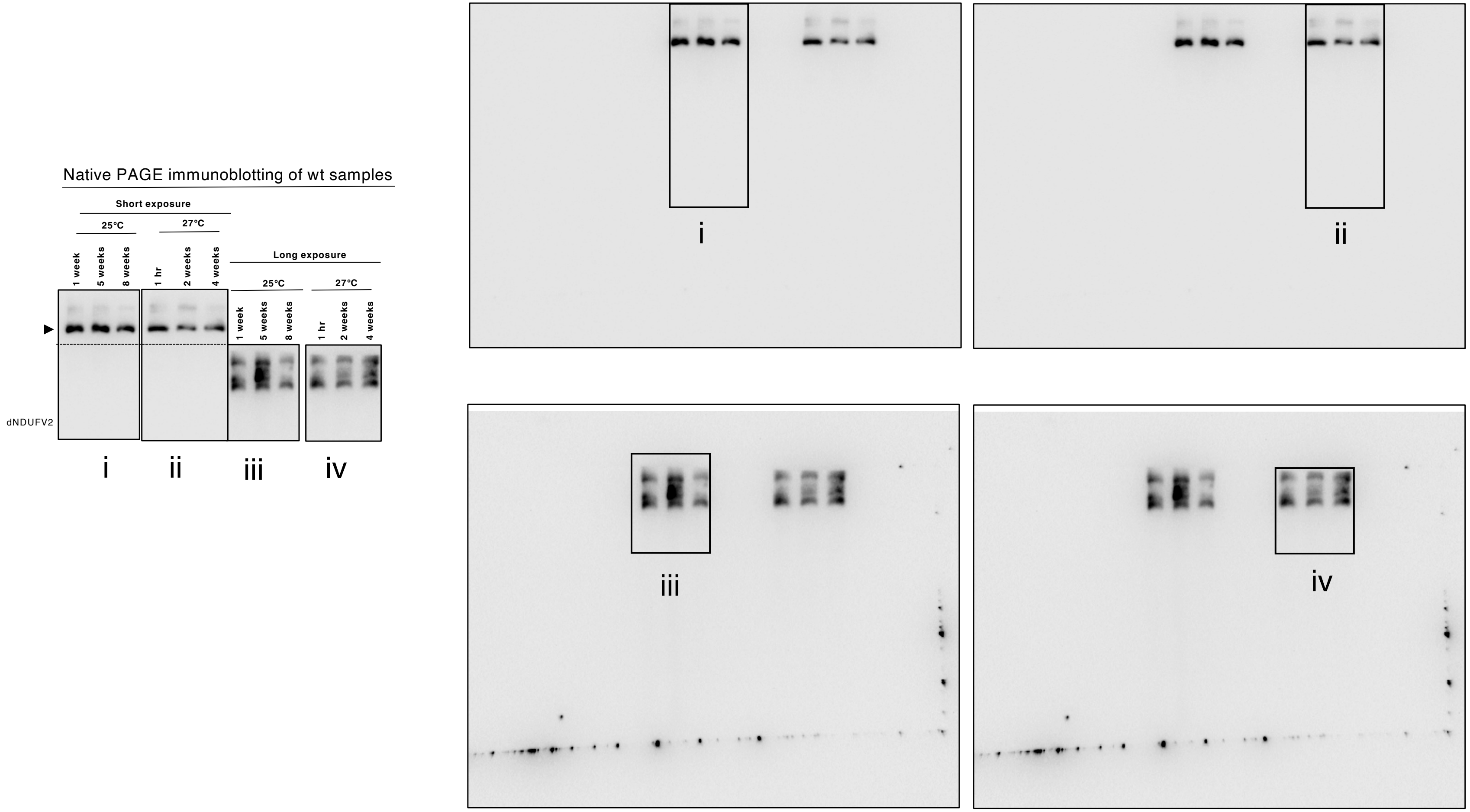

Native PAGE immunoblotting of wt samples
Short exposure
25°C
27°C
1 week
5 weeks
8 weeks
1 hr
2 weeks
4 weeks
Long exposure
25°C
27°C
1 week
5 weeks
8 weeks
1 hr
2 weeks
4 weeks
dNDUFV2
i
ii
iii
iv
i
ii
iii
iv

Figure 2D

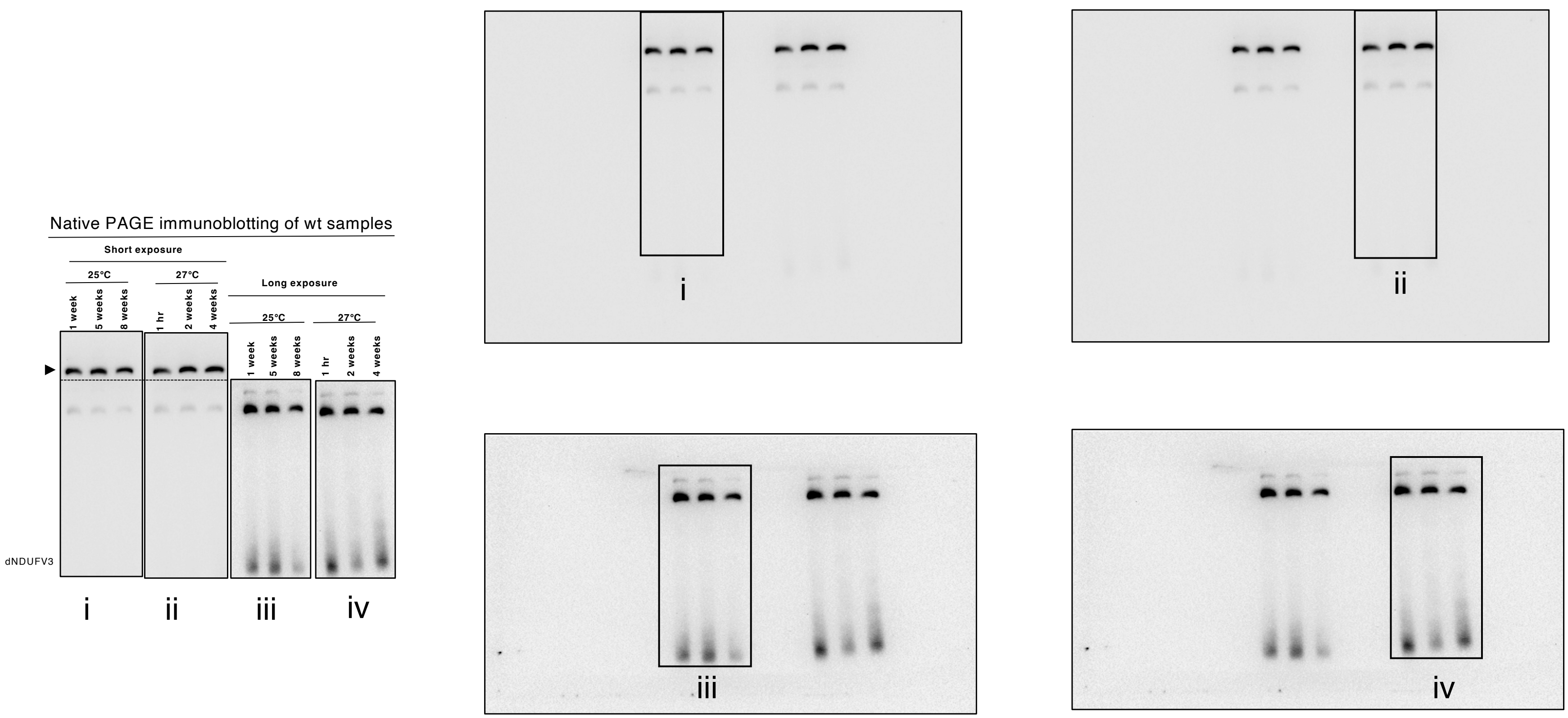
Native PAGE immunoblotting of wt samples
Short exposure
25°C
27°C
1 week
5 weeks
8 weeks
1 hr
2 weeks
4 weeks
Long exposure
25°C
27°C
1 week
5 weeks
8 weeks
1 hr
2 weeks
4 weeks
dNDUFV3
i
ii
iii
iv
i
ii
iii
iv

Figure 2E

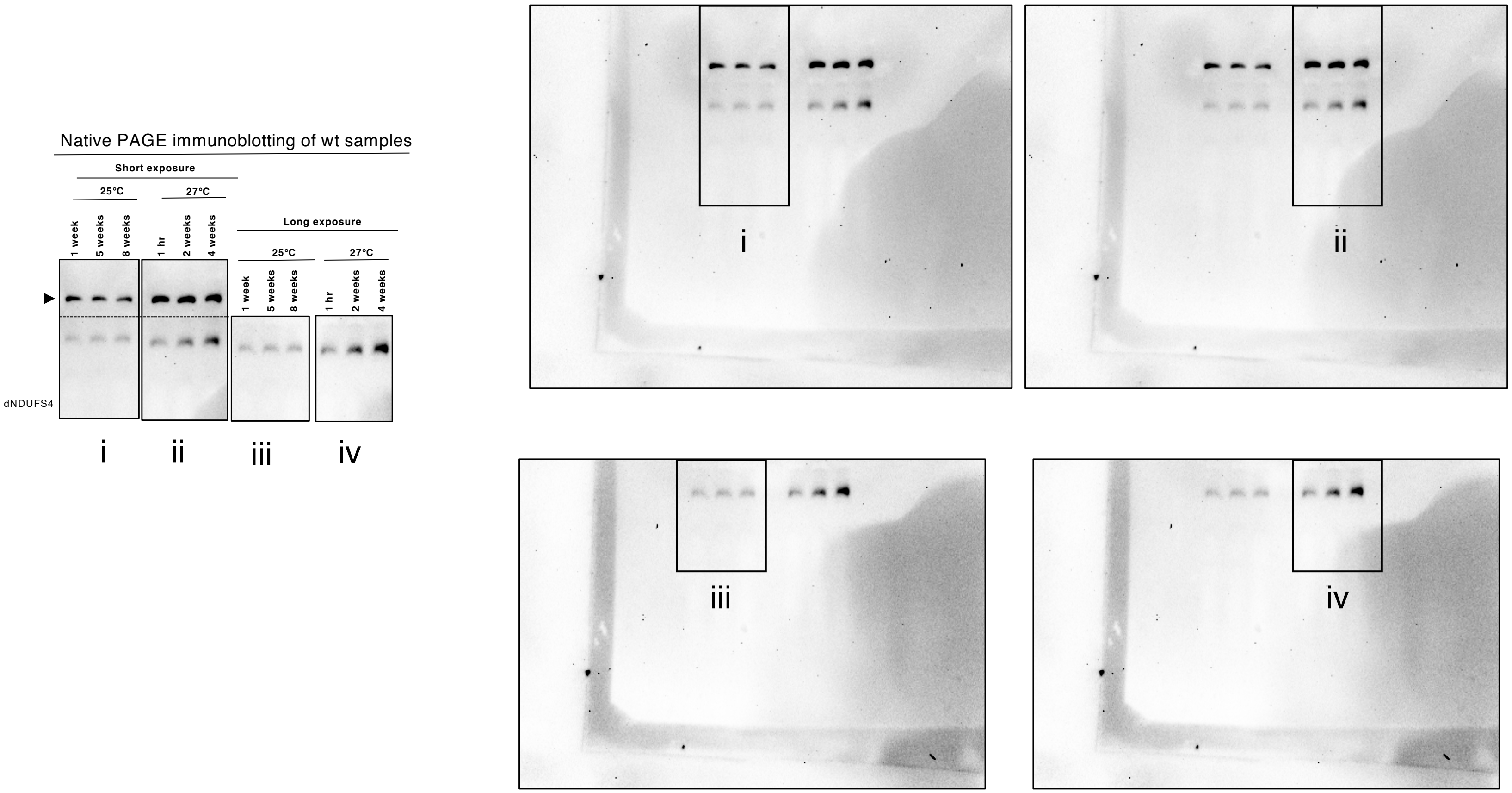

Native PAGE immunoblotting of wt samples
Short exposure
25°C
27°C
1 week
5 weeks
8 weeks
1 hr
2 weeks
4 weeks
Long exposure
25°C
27°C
1 week
5 weeks
8 weeks
1 hr
2 weeks
4 weeks
dNDUFS4
i
ii
iii
iv
i
ii
iii
iv

Figure 2F

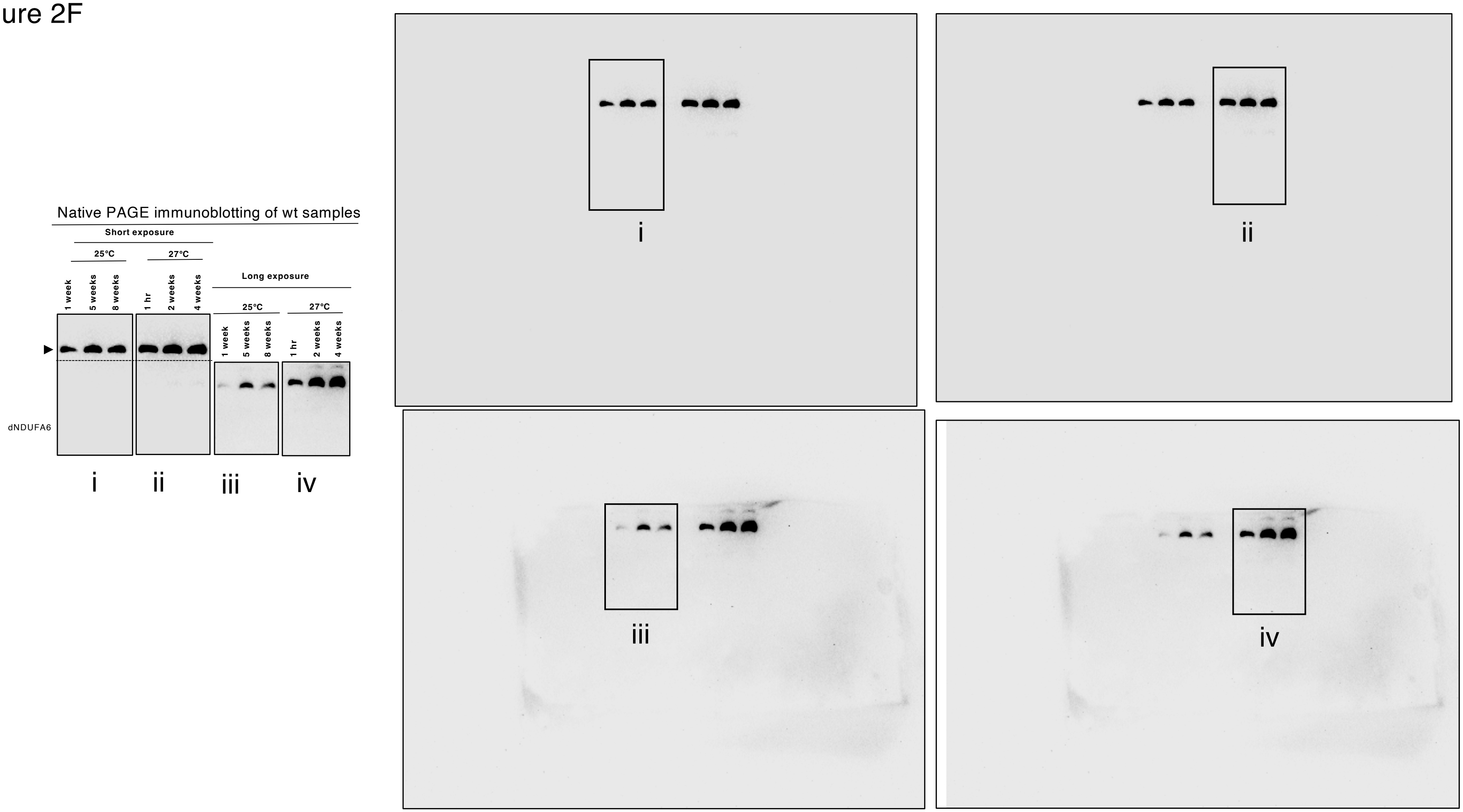
Native PAGE immunoblotting of wt samples
Short exposure
25°C
27°C
1 week
5 weeks
8 weeks
1 hr
2 weeks
4 weeks
Long exposure
25°C
27°C
1 week
5 weeks
8 weeks
1 hr
2 weeks
4 weeks
dNDUFA6
i
ii
iii
iv
i
ii
iii
iv

Figure 2G

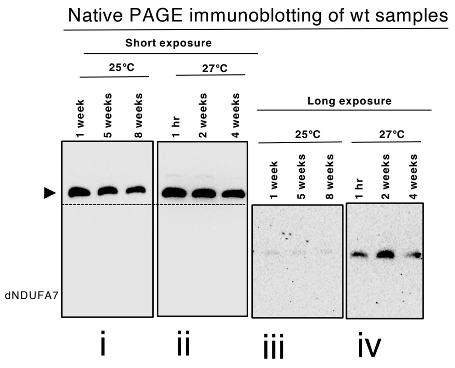

Native PAGE immunoblotting of wt samples
Short exposure
25°C
27°C
1 week
5 weeks
8 weeks
1 hr
2 weeks
4 weeks
Long exposure
25°C
27°C
1 week
5 weeks
8 weeks
1 hr
2 weeks
4 weeks
dNDUFA7
i
ii
iii
iv

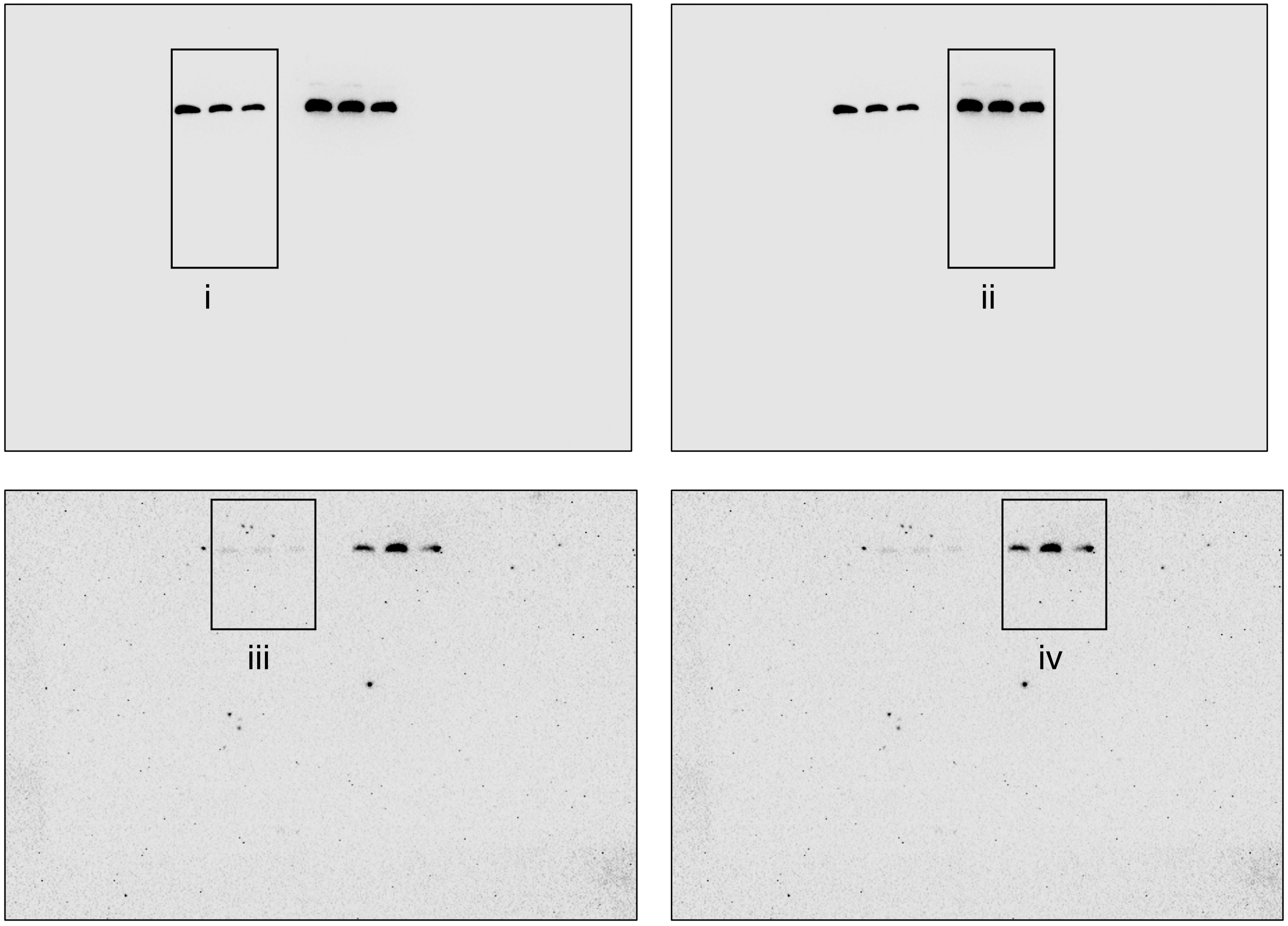

i
ii
iii
iv

Figure 2H

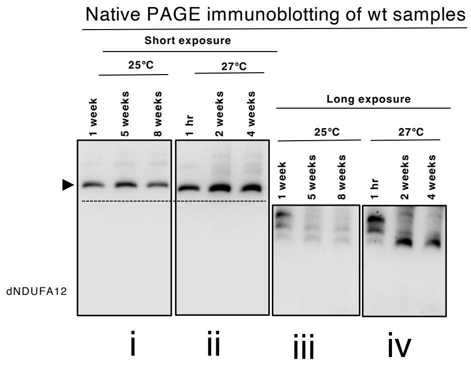

Native PAGE immunoblotting of wt samples
Short exposure
25°C
27°C
1 week
5 weeks
8 weeks
1 hr
2 weeks
4 weeks
Long exposure
25°C
27°C
1 week
5 weeks
8 weeks
1 hr
2 weeks
4 weeks
dNDUFA12
i
ii
iii
iv

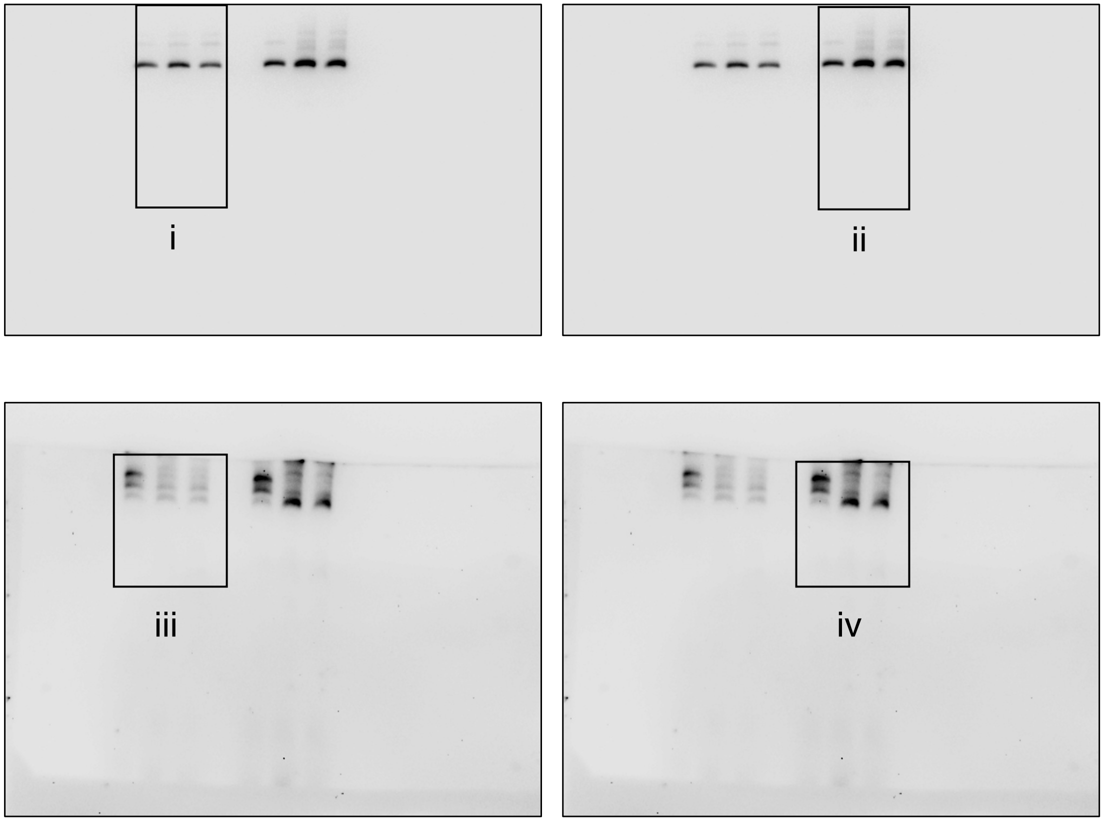

i
ii
iii
iv

Figure 3B

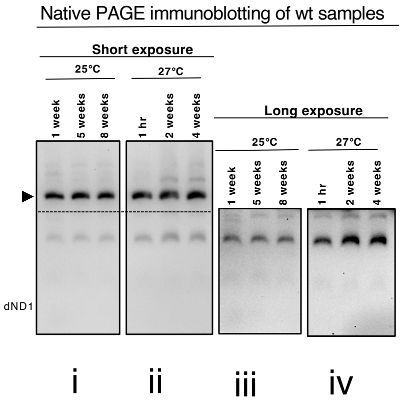

Native PAGE immunoblotting of wt samples
Short exposure
25°C
27°C
1 week
5 weeks
8 weeks
1 hr
2 weeks
4 weeks
Long exposure
25°C
27°C
1 week
5 weeks
8 weeks
1 hr
2 weeks
4 weeks
dND1
i
ii
iii
iv

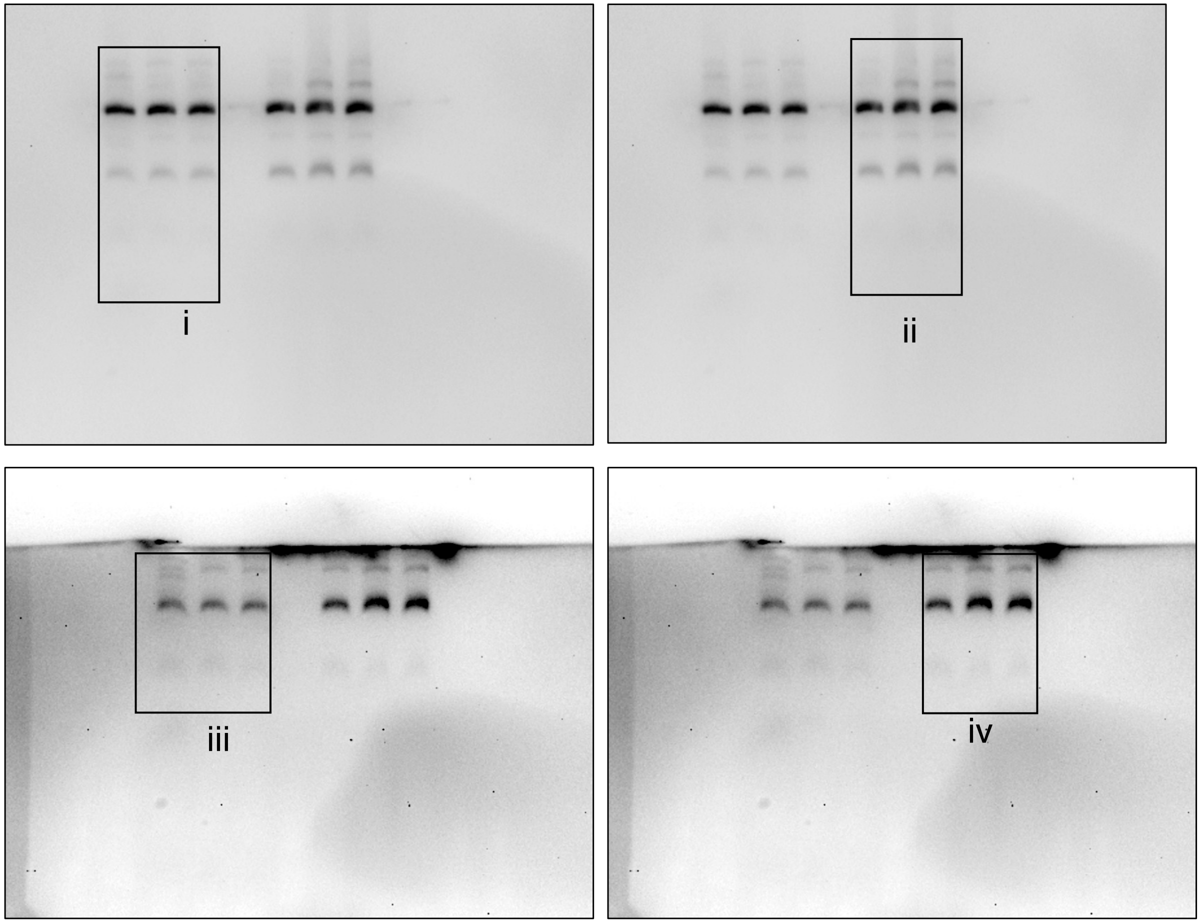

i
ii
iii
iv

Figure 3C

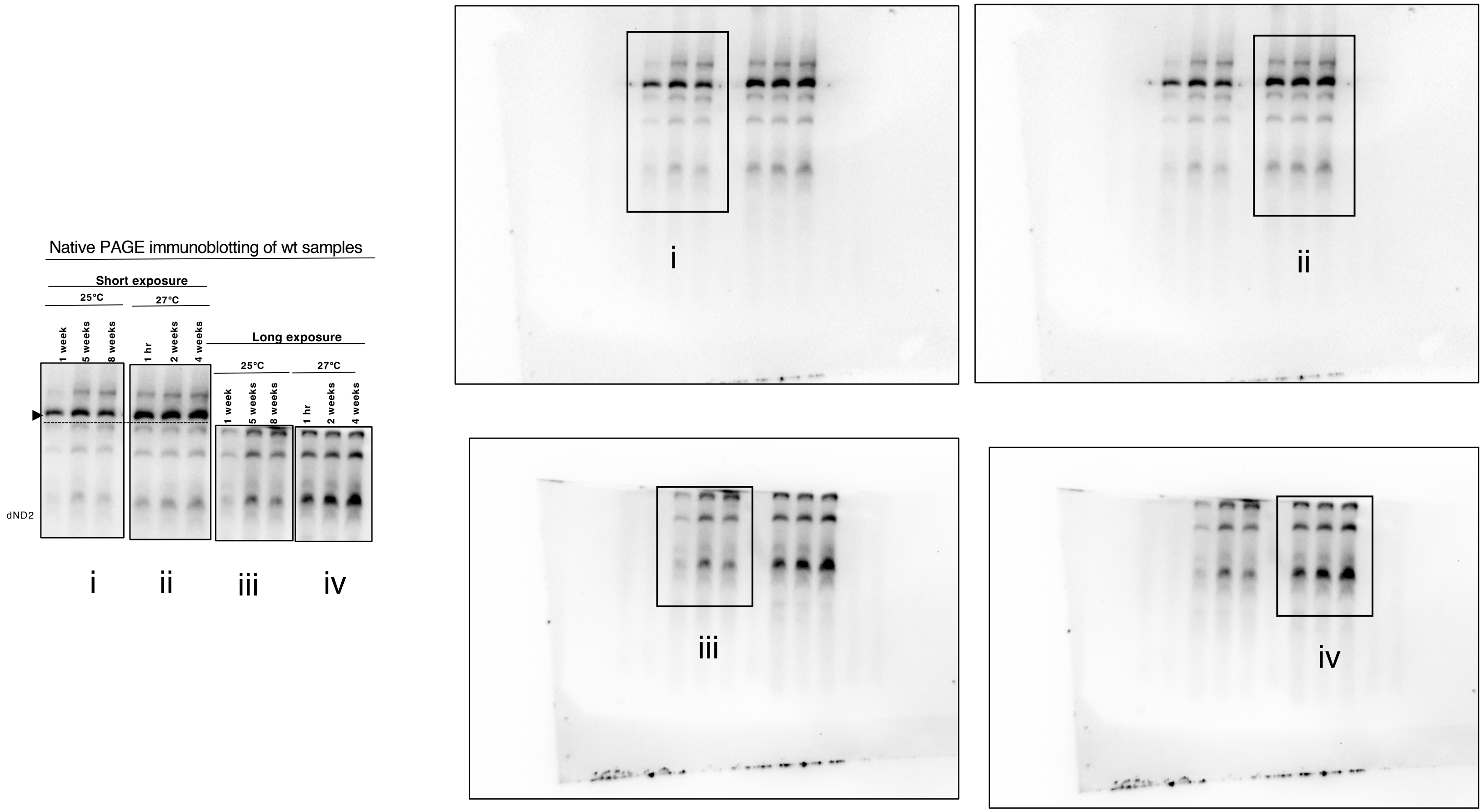

Native PAGE immunoblotting of wt samples
Short exposure
25°C
27°C
1 week
5 weeks
8 weeks
1 hr
2 weeks
4 weeks
Long exposure
25°C
27°C
1 week
5 weeks
8 weeks
1 hr
2 weeks
4 weeks
dND2
i
ii
iii
iv
i
ii
iii
iv

Figure 3D

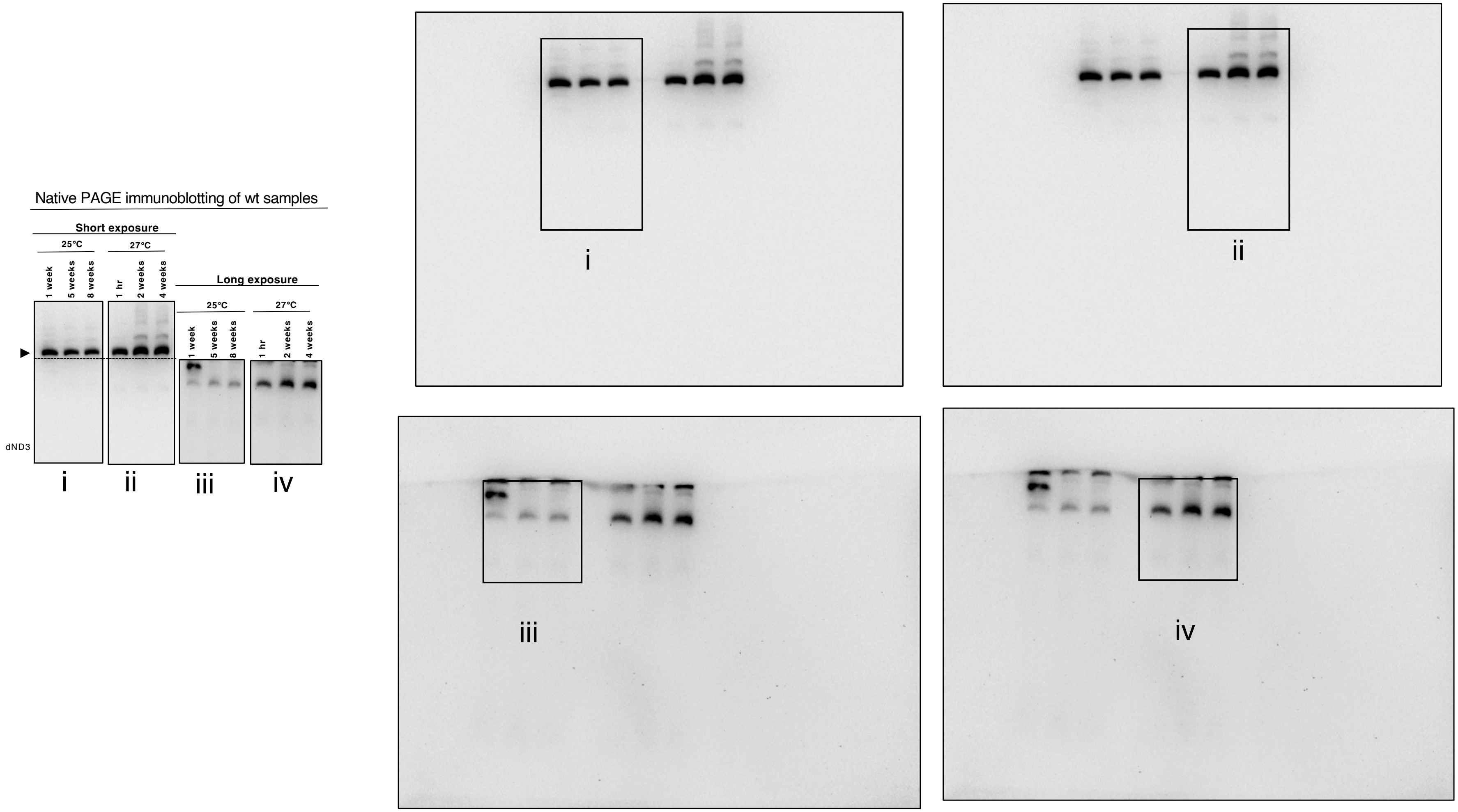

Native PAGE immunoblotting of wt samples
Short exposure
25°C
27°C
1 week
5 weeks
8 weeks
1 hr
2 weeks
4 weeks
Long exposure
25°C
27°C
1 week
5 weeks
8 weeks
1 hr
2 weeks
4 weeks
dND3
i
ii
iii
iv
i
ii
iii
iv

Figure 3E

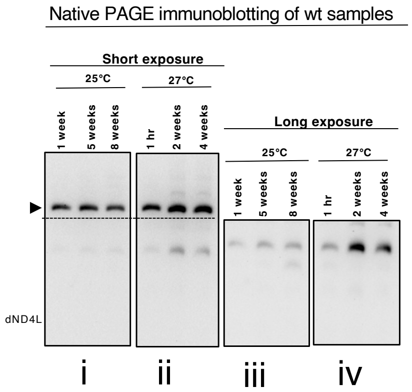

Native PAGE immunoblotting of wt samples
Short exposure
25°C
27°C
1 week
5 weeks
8 weeks
1 hr
2 weeks
4 weeks
Long exposure
25°C
27°C
1 week
5 weeks
8 weeks
1 hr
2 weeks
4 weeks
dND4L
i
ii
iii
iv

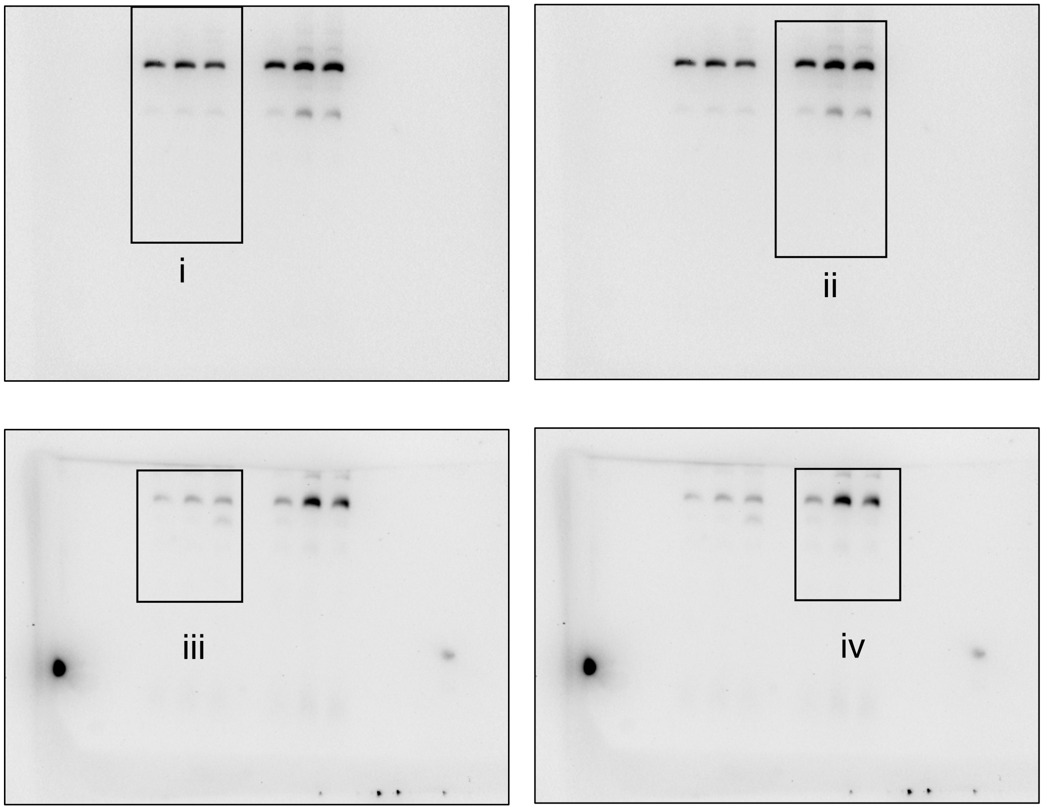

i
ii
iii
iv

Figure 3F

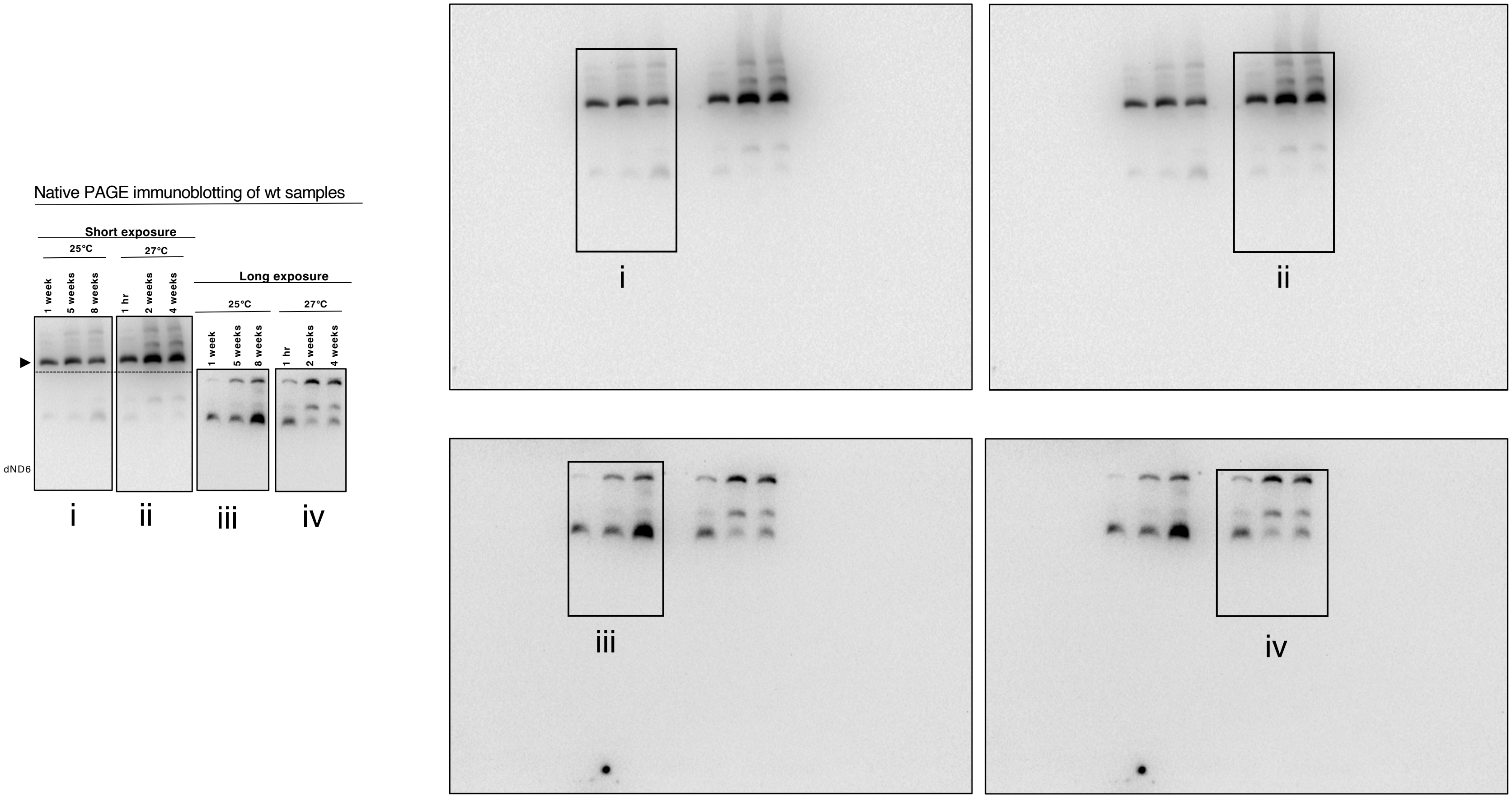
Native PAGE immunoblotting of wt samples
Short exposure
25°C
27°C
1 week
5 weeks
8 weeks
1 hr
2 weeks
4 weeks
Long exposure
25°C
27°C
1 week
5 weeks
8 weeks
1 hr
2 weeks
4 weeks
dND6
i
ii
iii
iv
i
ii
iii
iv

Figure 3G

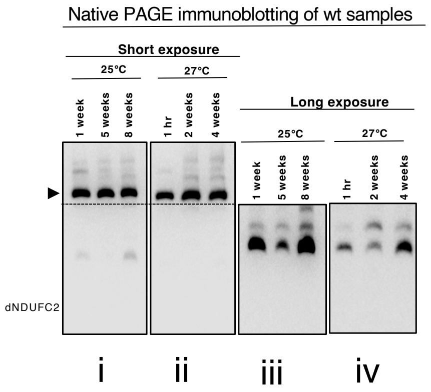

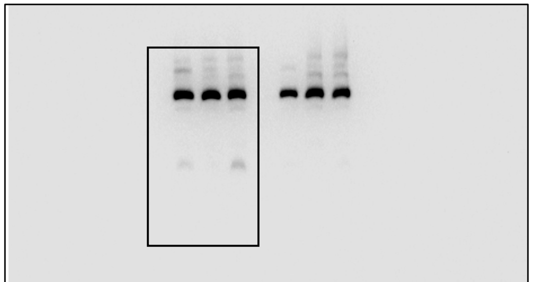

i

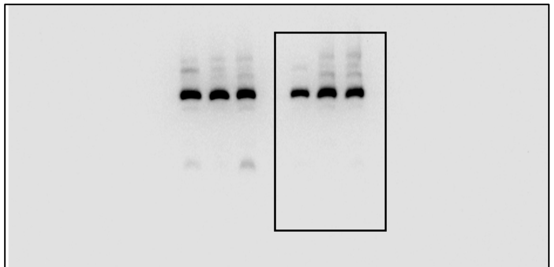

ii

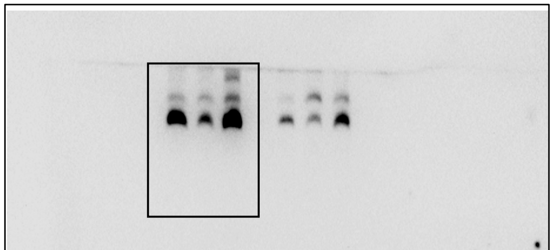

iii

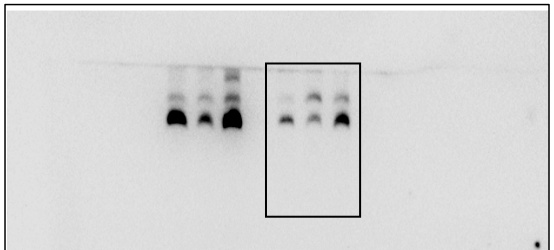

iv

Figure 3H

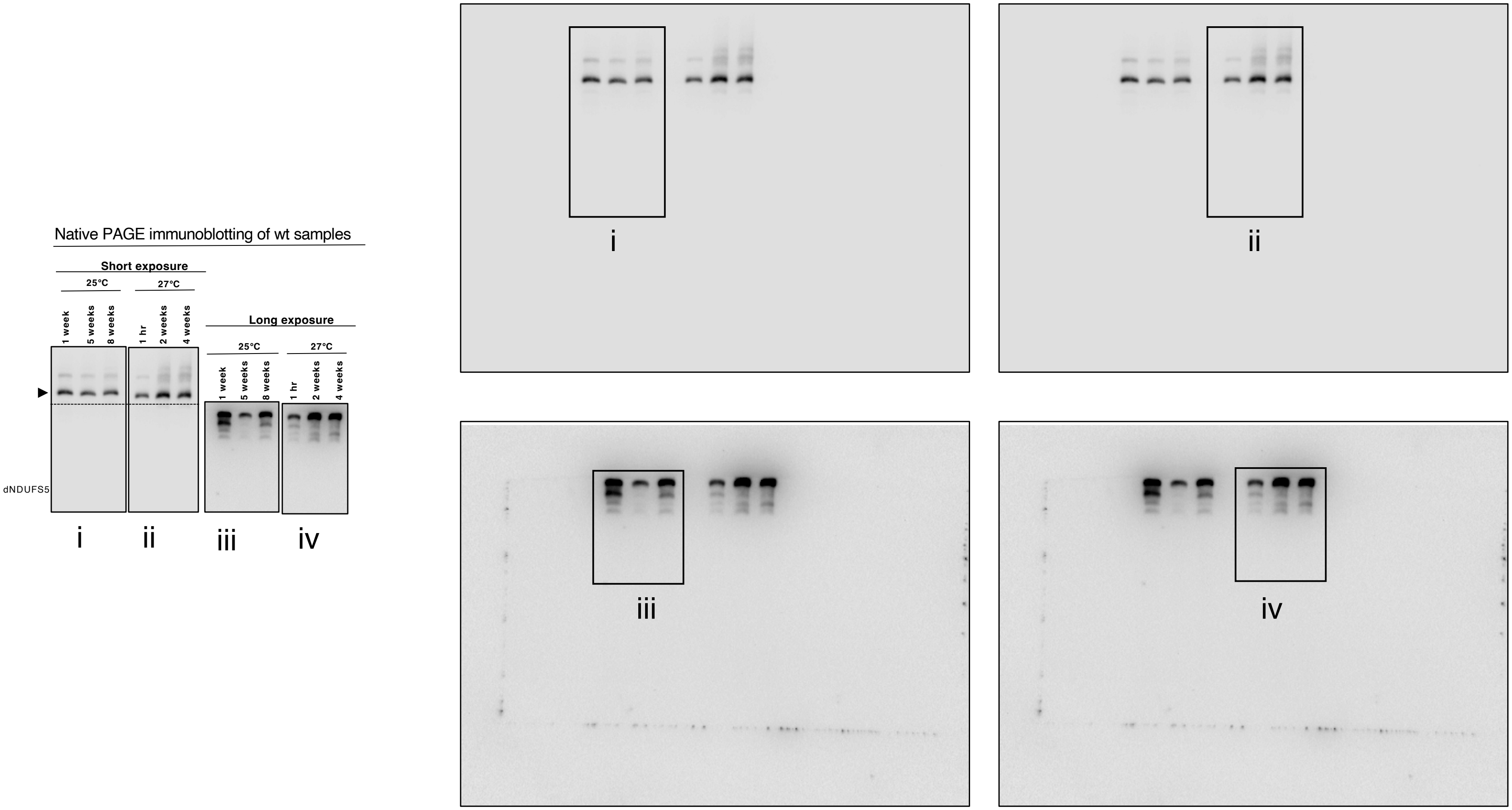
Native PAGE immunoblotting of wt samples
Short exposure
25°C
27°C
1 week
5 weeks
8 weeks
1 hr
2 weeks
4 weeks
Long exposure
25°C
27°C
1 week
5 weeks
8 weeks
1 hr
2 weeks
4 weeks
dNDUFS5
i
ii
iii
iv
i
ii
iii
iv

Figure 3I

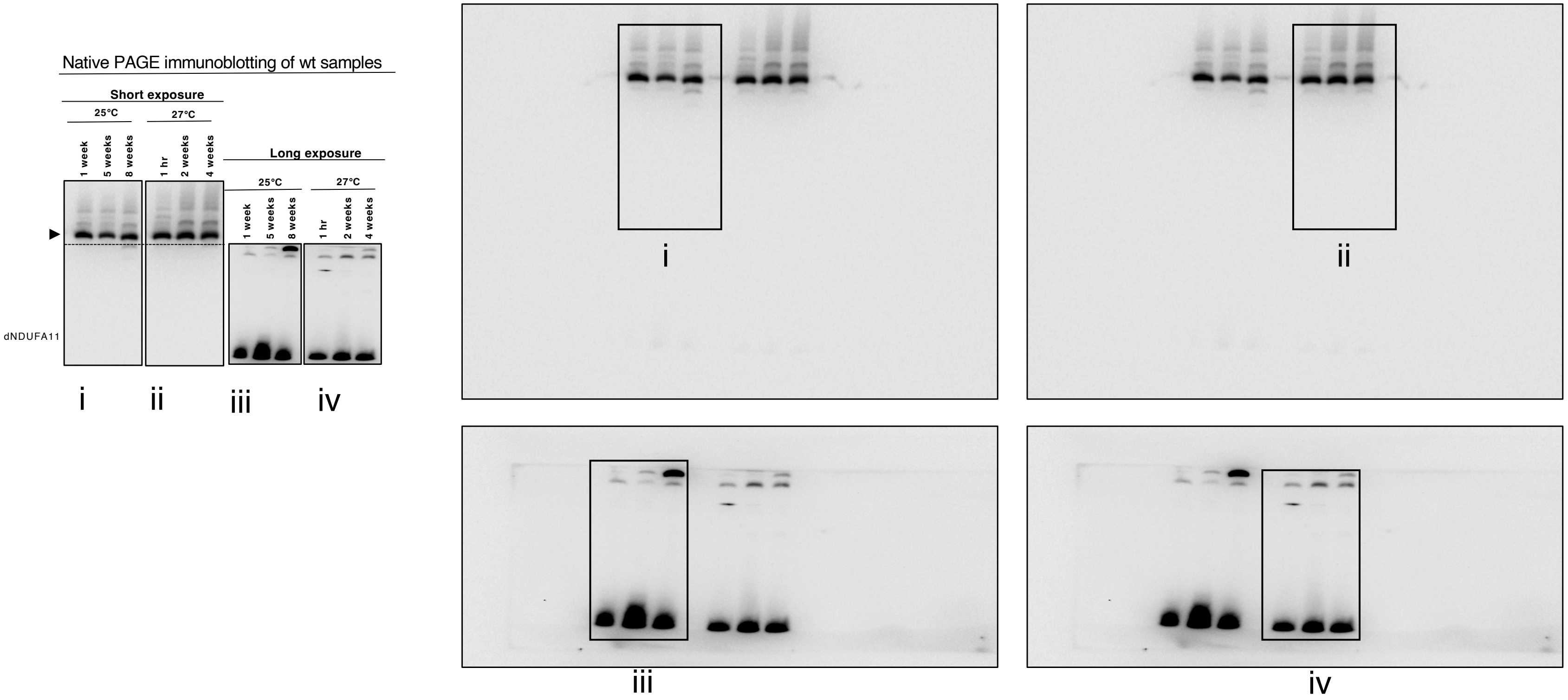
Native PAGE immunoblotting of wt samples
Short exposure
25°C
27°C
1 week
5 weeks
8 weeks
1 hr
2 weeks
4 weeks
Long exposure
25°C
27°C
1 week
5 weeks
8 weeks
1 hr
2 weeks
4 weeks
dNDUFA11
i
ii
iii
iv
i
ii
iii
iv

Figure 3J

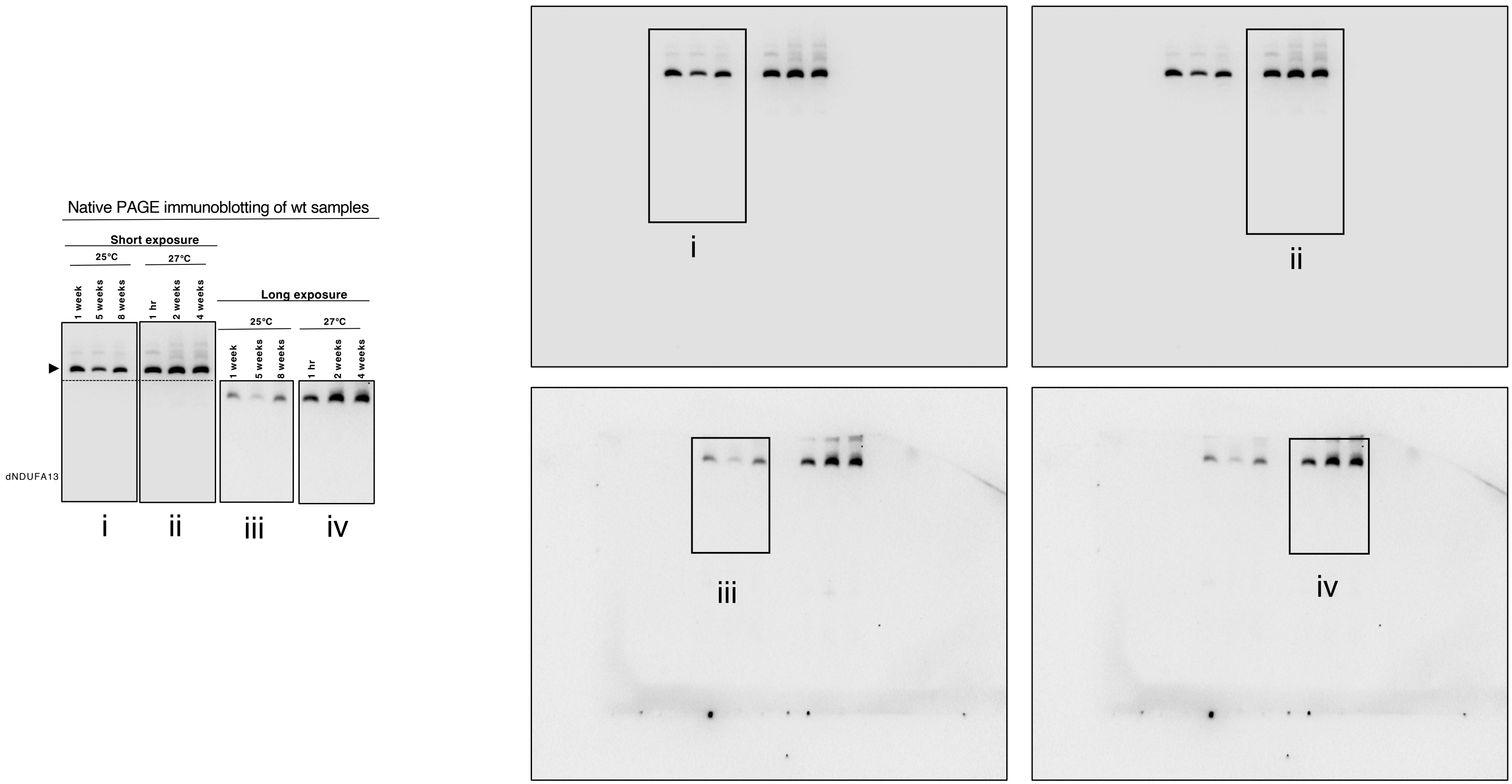

Native PAGE immunoblotting of wt samples
Short exposure
25°C
27°C
1 week
5 weeks
8 weeks
1 hr
2 weeks
4 weeks
Long exposure
25°C
27°C
1 week
5 weeks
8 weeks
1 hr
2 weeks
4 weeks
dNDUFA13
i
ii
iii
iv
i
ii
iii
iv

Figure 3K

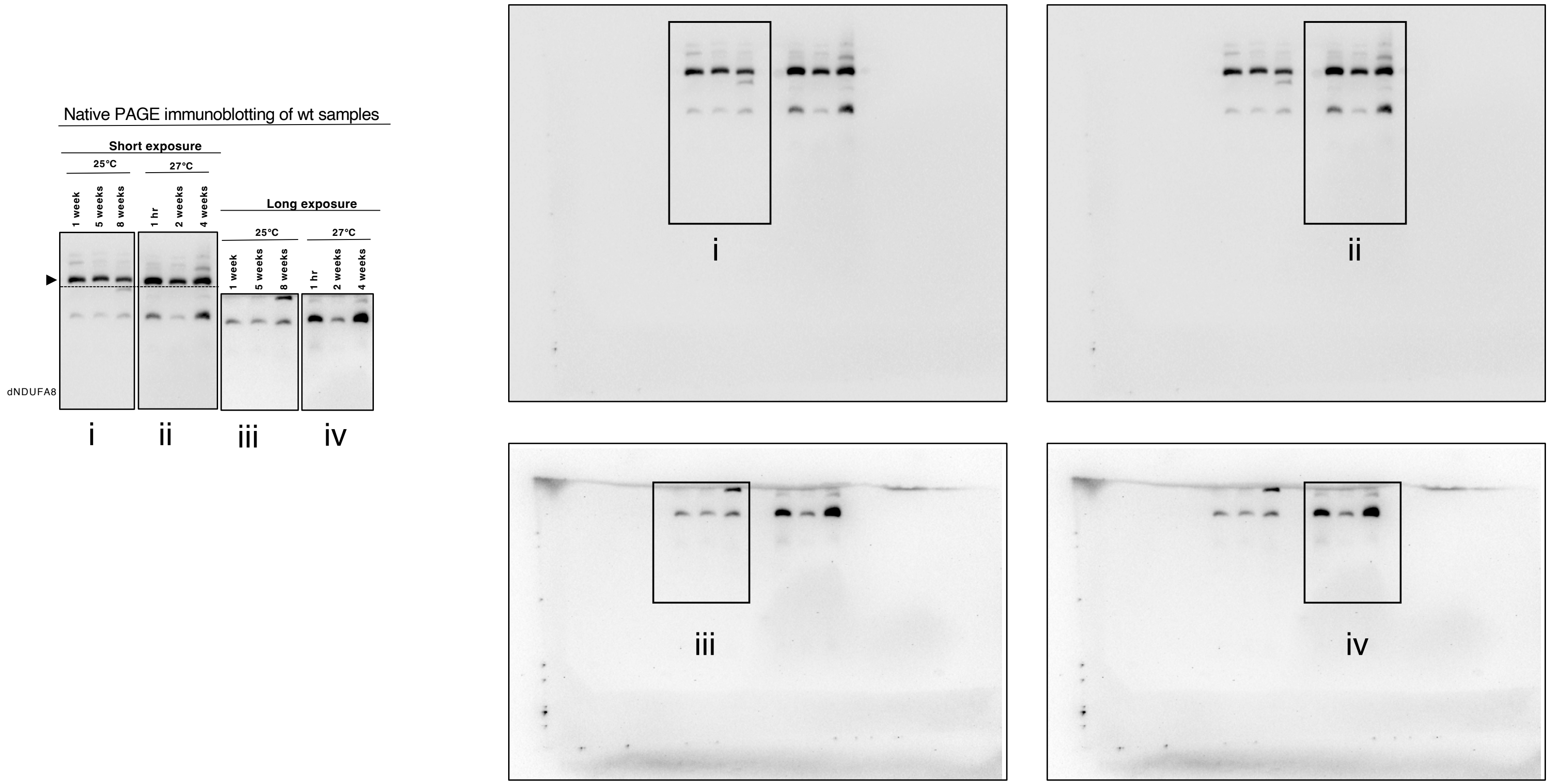

Native PAGE immunoblotting of wt samples
Short exposure
25°C
27°C
1 week
5 weeks
8 weeks
1 hr
2 weeks
4 weeks
Long exposure
25°C
27°C
1 week
5 weeks
8 weeks
1 hr
2 weeks
4 weeks
dNDUFA8
i
ii
iii
iv
i
ii
iii
iv

Figure 3L

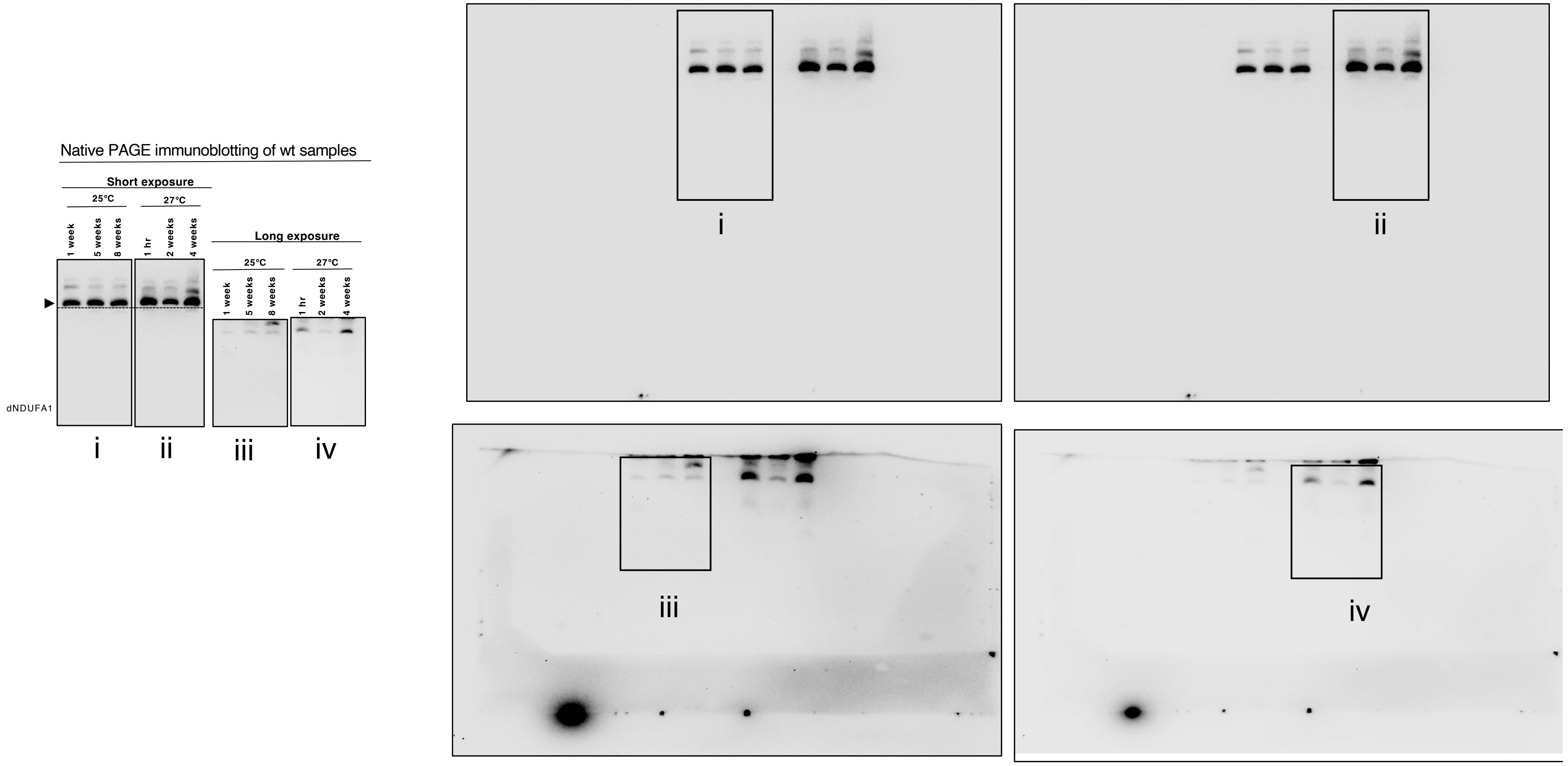

Native PAGE immunoblotting of wt samples
Short exposure
25°C
27°C
1 week
5 weeks
8 weeks
1 hr
2 weeks
4 weeks
Long exposure
25°C
27°C
1 week
5 weeks
8 weeks
1 hr
2 weeks
4 weeks
dNDUFA1
i
ii
iii
iv
i
ii
iii
iv

Figure 4A

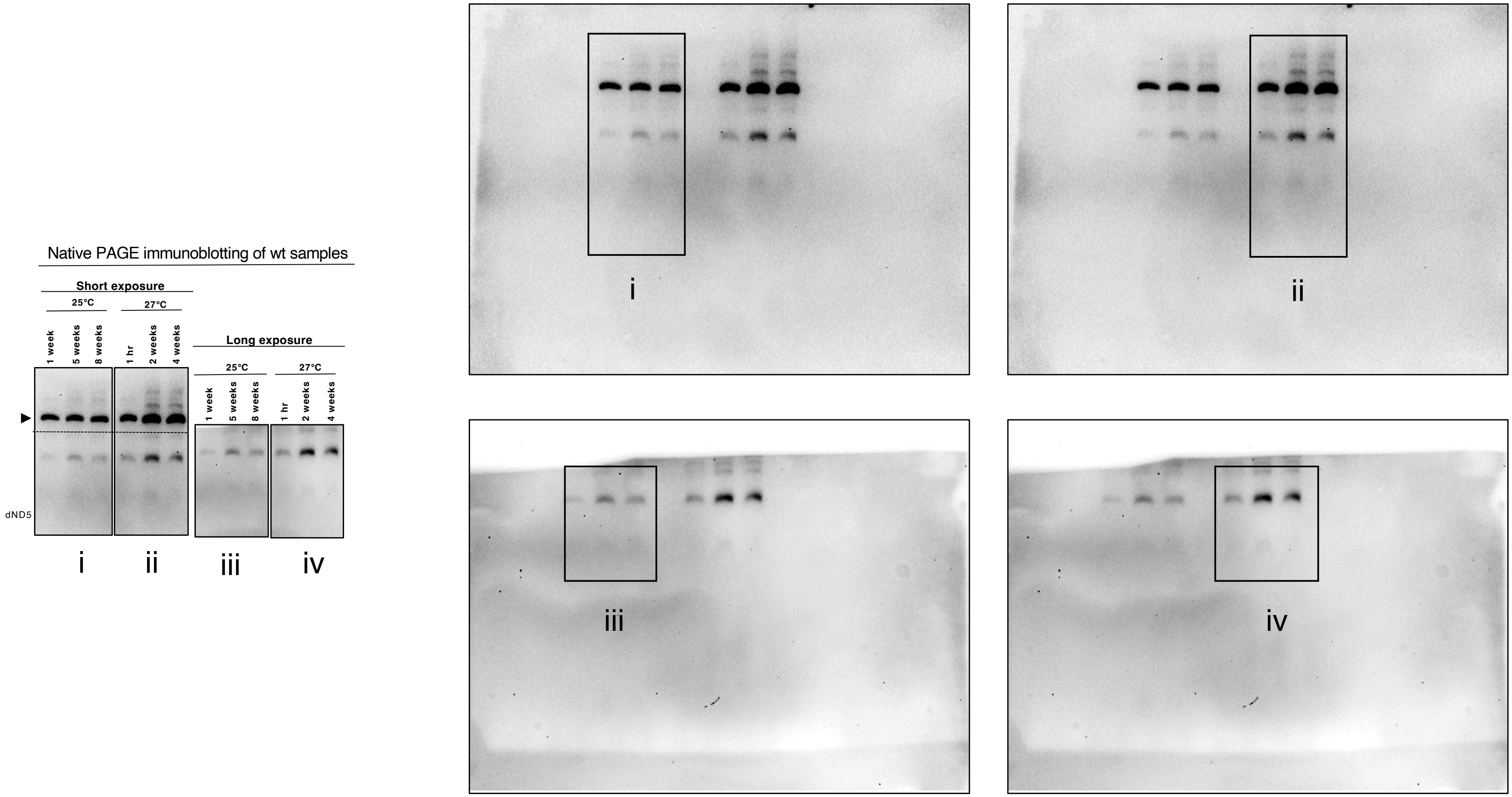
Native PAGE immunoblotting of wt samples
Short exposure
25°C
27°C
1 week
5 weeks
8 weeks
1 hr
2 weeks
4 weeks
Long exposure
25°C
27°C
1 week
5 weeks
8 weeks
1 hr
2 weeks
4 weeks
dND5
i
ii
iii
iv
i
ii
iii
iv

Figure 4B

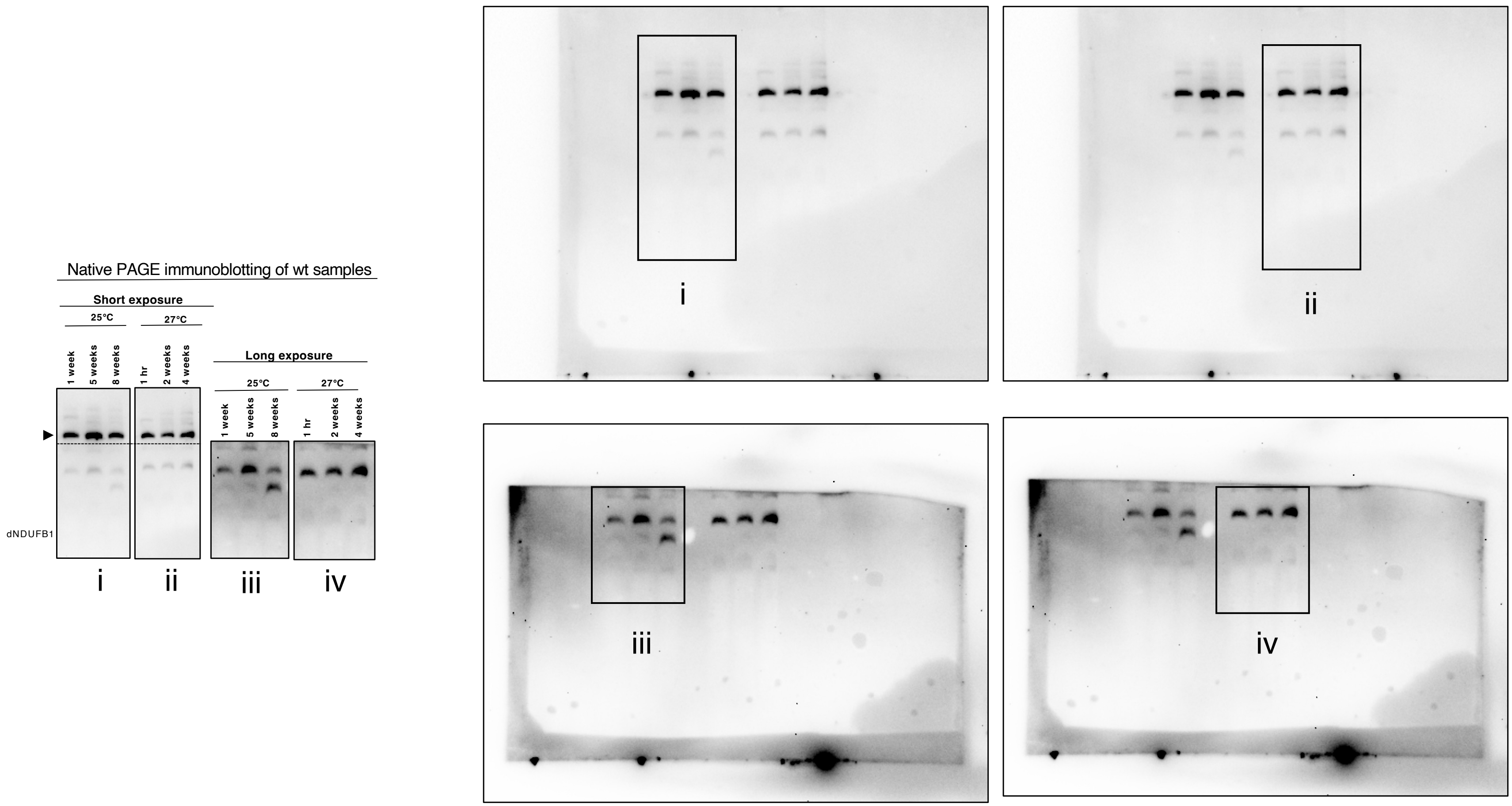

Native PAGE immunoblotting of wt samples
Short exposure
25°C
27°C
1 week
5 weeks
8 weeks
1 hr
2 weeks
4 weeks
Long exposure
25°C
27°C
1 week
5 weeks
8 weeks
1 hr
2 weeks
4 weeks
dNDUFB1
i
ii
iii
iv
i
ii
iii
iv

Figure 4C

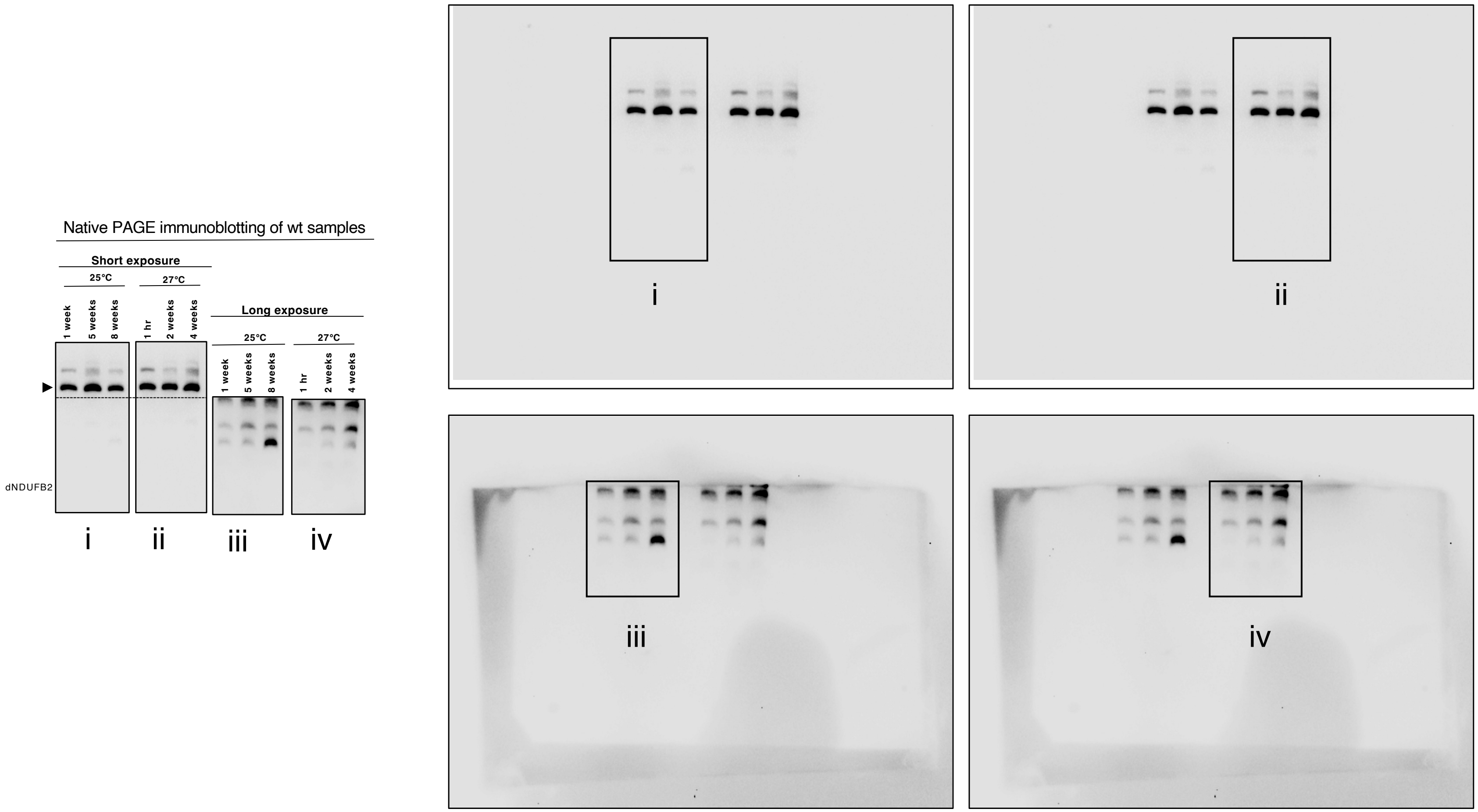
Native PAGE immunoblotting of wt samples
Short exposure
25°C
27°C
1 week
5 weeks
8 weeks
1 hr
2 weeks
4 weeks
Long exposure
25°C
27°C
1 week
5 weeks
8 weeks
1 hr
2 weeks
4 weeks
dNDUFB2
i
ii
iii
iv
i
ii
iii
iv

Figure 4D

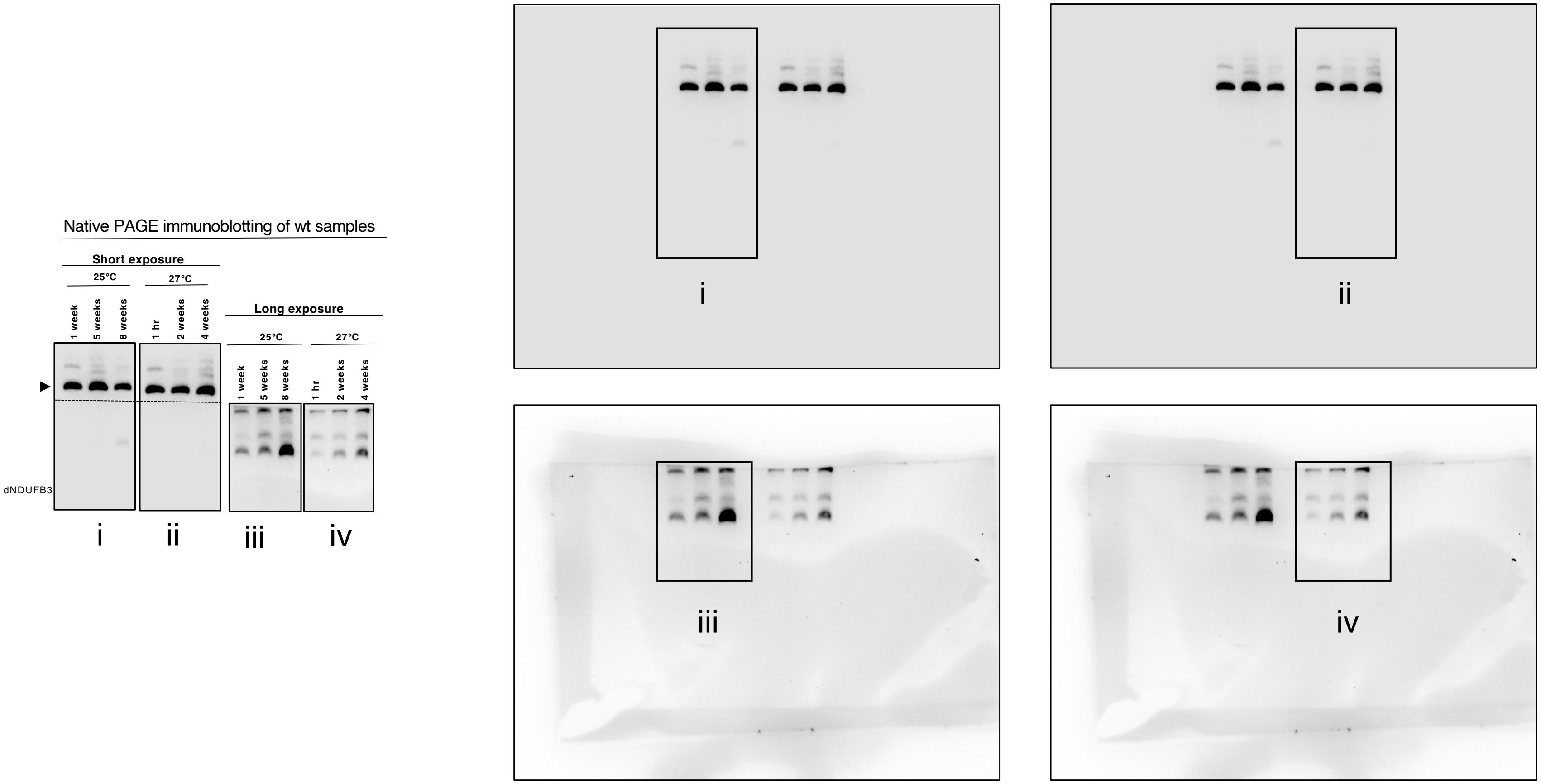

Native PAGE immunoblotting of wt samples
Short exposure
25°C
27°C
1 week
5 weeks
8 weeks
1 hr
2 weeks
4 weeks
Long exposure
25°C
27°C
1 week
5 weeks
8 weeks
1 hr
2 weeks
4 weeks
dNDUFB3
i
ii
iii
iv
i
ii
iii
iv

Figure 4E

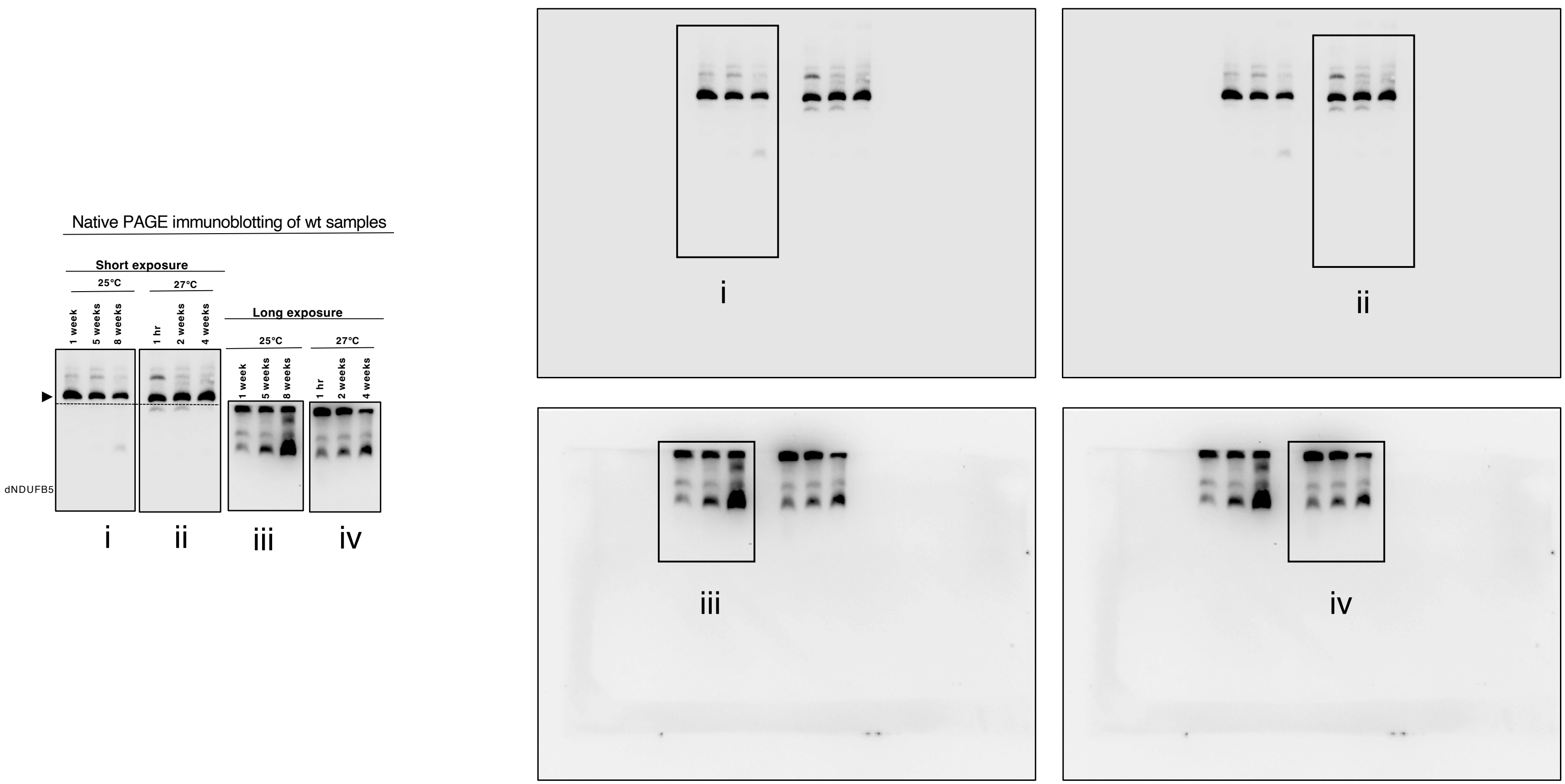

Native PAGE immunoblotting of wt samples
Short exposure
25°C
27°C
1 week
5 weeks
8 weeks
1 hr
2 weeks
4 weeks
Long exposure
25°C
27°C
1 week
5 weeks
8 weeks
1 hr
2 weeks
4 weeks
dNDUFB5
i
ii
iii
iv
i
ii
iii
iv

Figure 4F

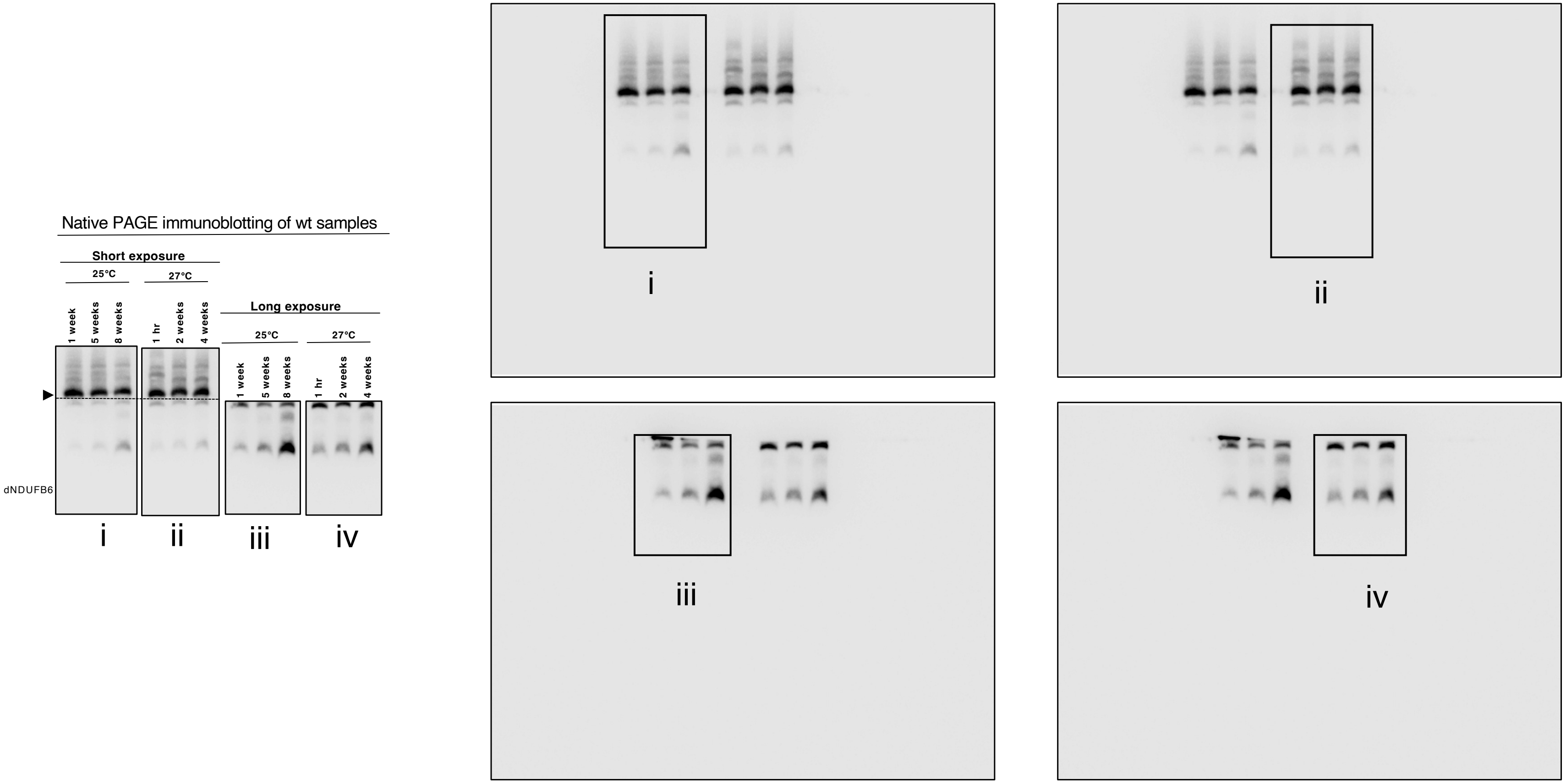
Native PAGE immunoblotting of wt samples
Short exposure
25°C
27°C
1 week
5 weeks
8 weeks
1 hr
2 weeks
4 weeks
Long exposure
25°C
27°C
1 week
5 weeks
8 weeks
1 hr
2 weeks
4 weeks
dNDUFB6
i
ii
iii
iv
i
ii
iii
iv

Figure 4G

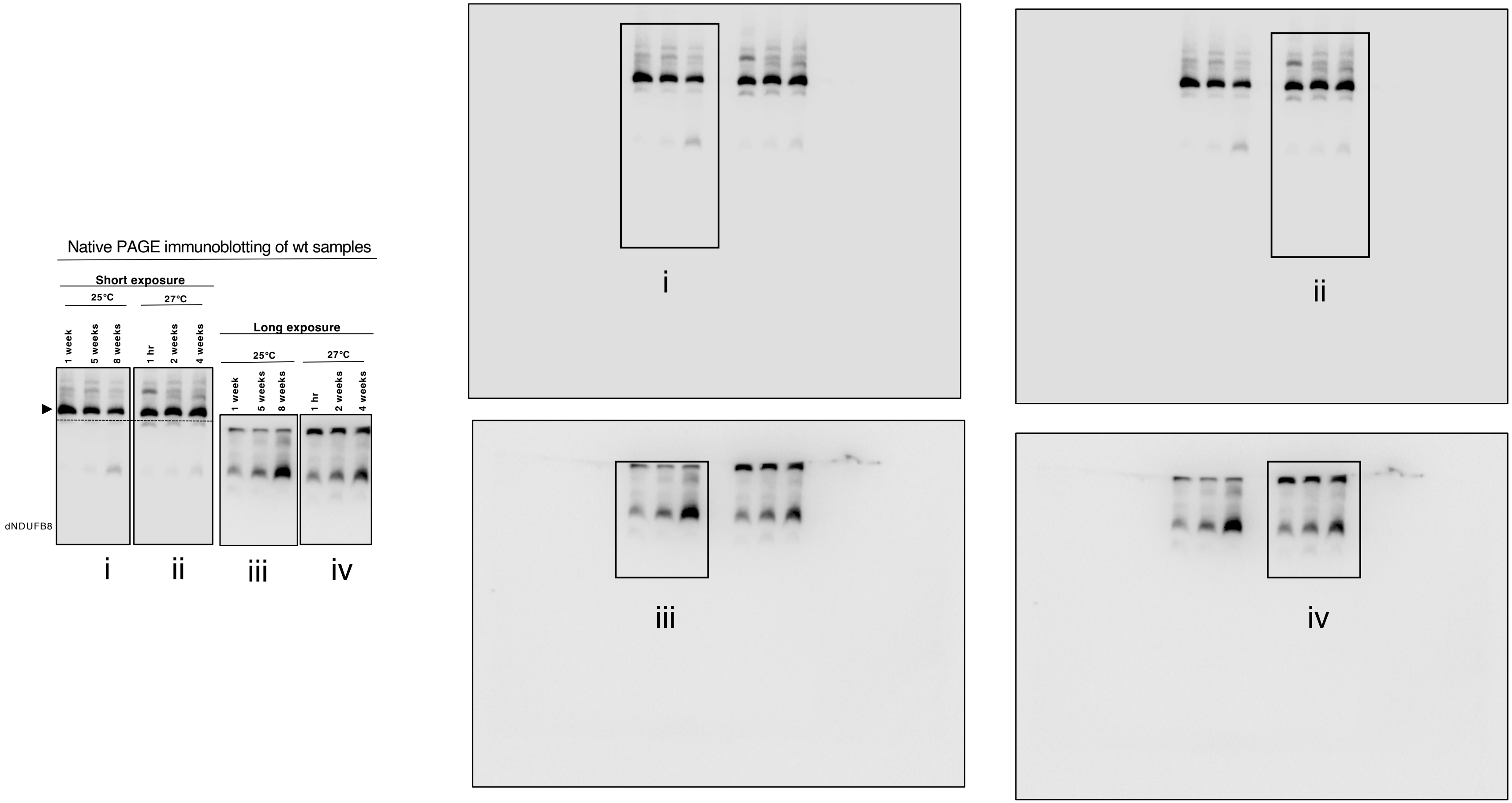

Native PAGE immunoblotting of wt samples
Short exposure
25°C
27°C
1 week
5 weeks
8 weeks
1 hr
2 weeks
4 weeks
Long exposure
25°C
27°C
1 week
5 weeks
8 weeks
1 hr
2 weeks
4 weeks
dNDUFB8
i
ii
iii
iv
i
ii
iii
iv

Figure 4H

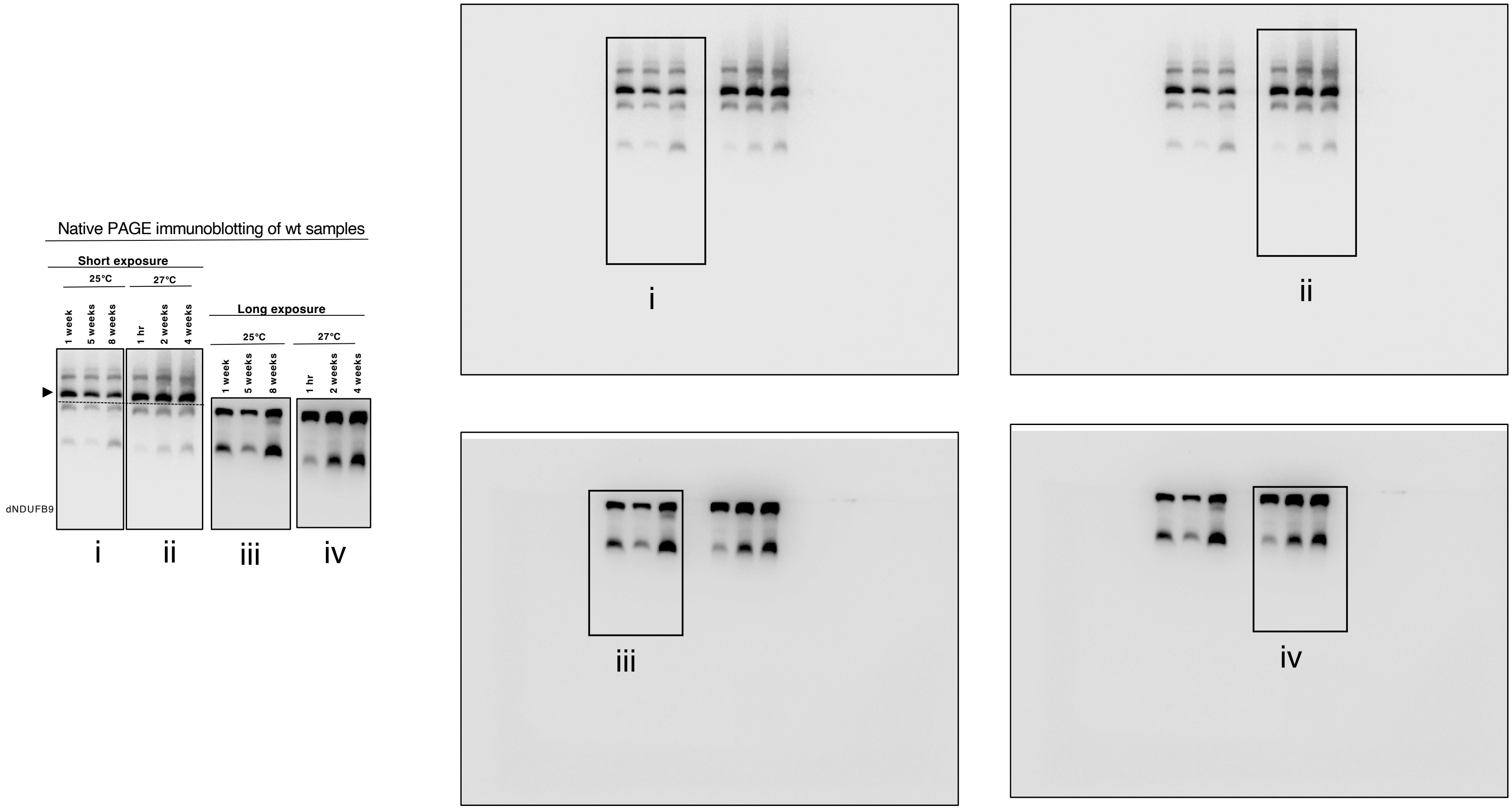

Native PAGE immunoblotting of wt samples
Short exposure
25°C
27°C
1 week
5 weeks
8 weeks
1 hr
2 weeks
4 weeks
Long exposure
25°C
27°C
1 week
5 weeks
8 weeks
1 hr
2 weeks
4 weeks
dNDUFB9
i
ii
iii
iv
i
ii
iii
iv

Figure 4I

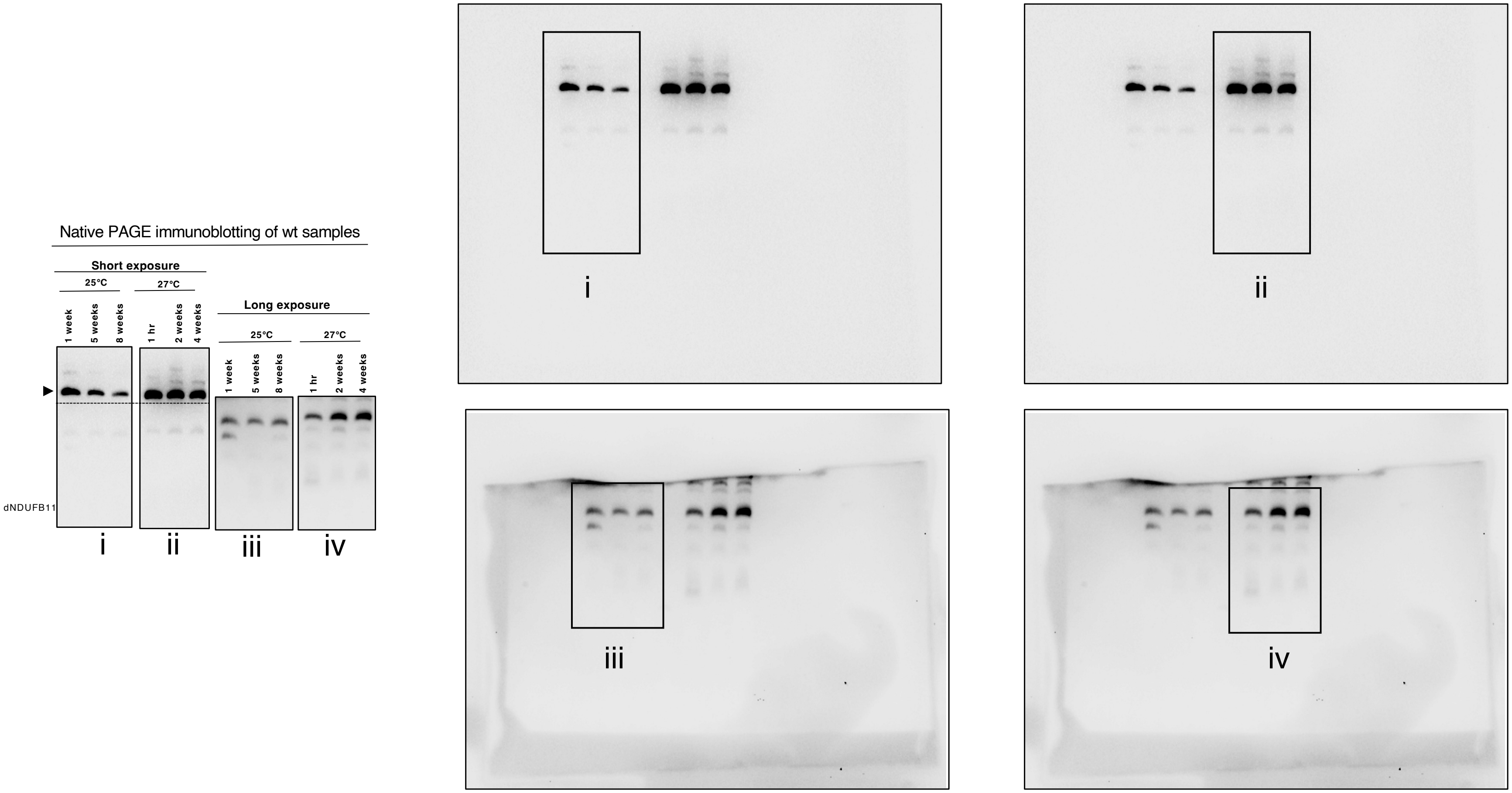

Native PAGE immunoblotting of wt samples
Short exposure
25°C
27°C
1 week
5 weeks
8 weeks
1 hr
2 weeks
4 weeks
Long exposure
25°C
27°C
1 week
5 weeks
8 weeks
1 hr
2 weeks
4 weeks
dNDUFB11
i
ii
iii
iv
i
ii
iii
iv

Figure 4J

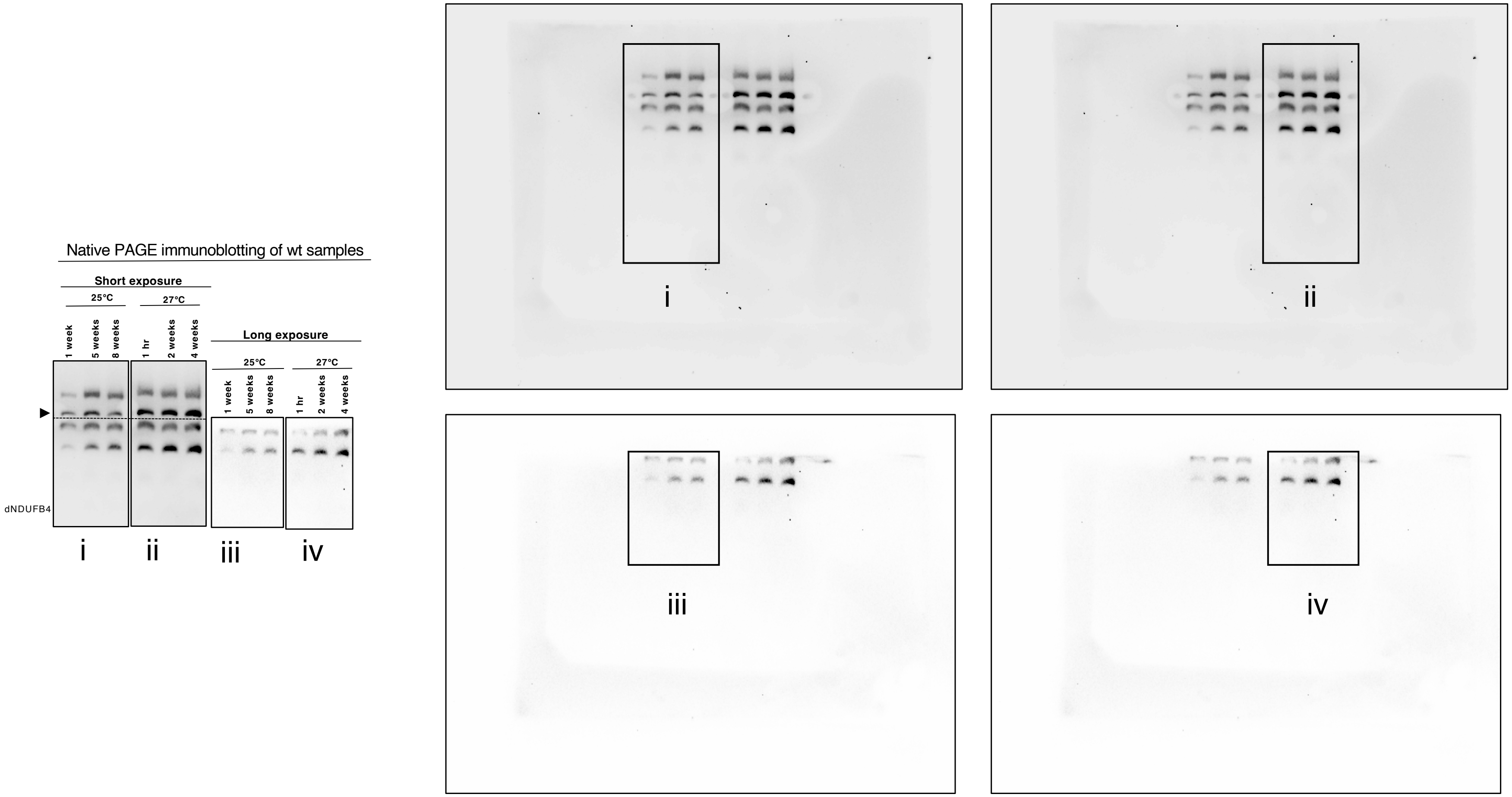

Native PAGE immunoblotting of wt samples
Short exposure
25°C
27°C
1 week
5 weeks
8 weeks
1 hr
2 weeks
4 weeks
Long exposure
25°C
27°C
1 week
5 weeks
8 weeks
1 hr
2 weeks
4 weeks
dNDUFB4
i
ii
iii
iv
i
ii
iii
iv

Figure 4K

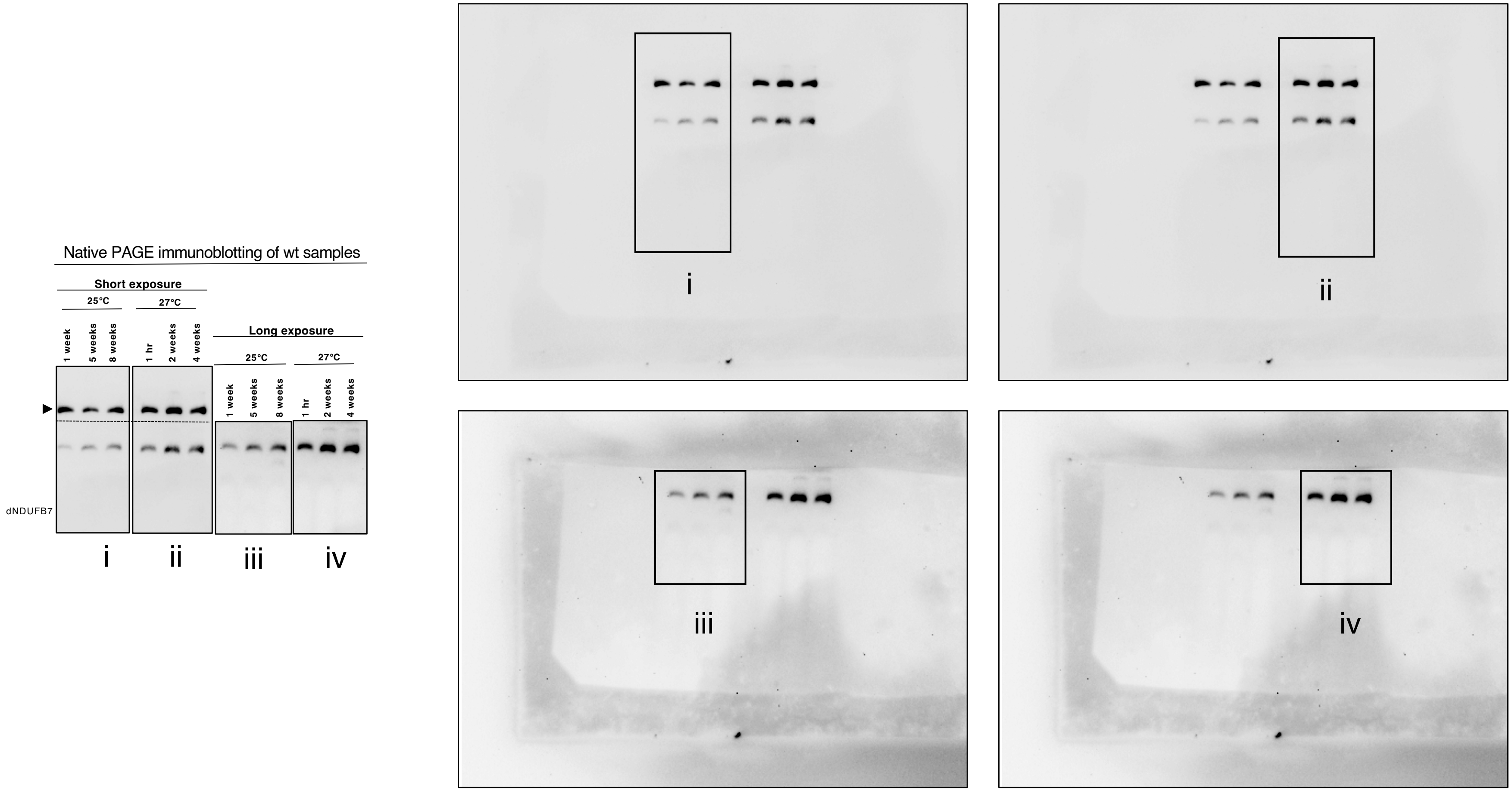

Native PAGE immunoblotting of wt samples
Short exposure
25°C
27°C
1 week
5 weeks
8 weeks
1 hr
2 weeks
4 weeks
Long exposure
25°C
27°C
1 week
5 weeks
8 weeks
1 hr
2 weeks
4 weeks
dNDUFB7
i
ii
iii
iv
i
ii
iii
iv

Figure 4L

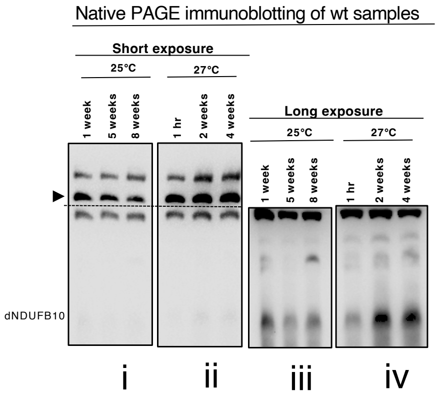

Native PAGE immunoblotting of wt samples
Short exposure
25°C
27°C
1 week
5 weeks
8 weeks
1 hr
2 weeks
4 weeks
Long exposure
25°C
27°C
1 week
5 weeks
8 weeks
1 hr
2 weeks
4 weeks
dNDUFB10
i
ii
iii
iv

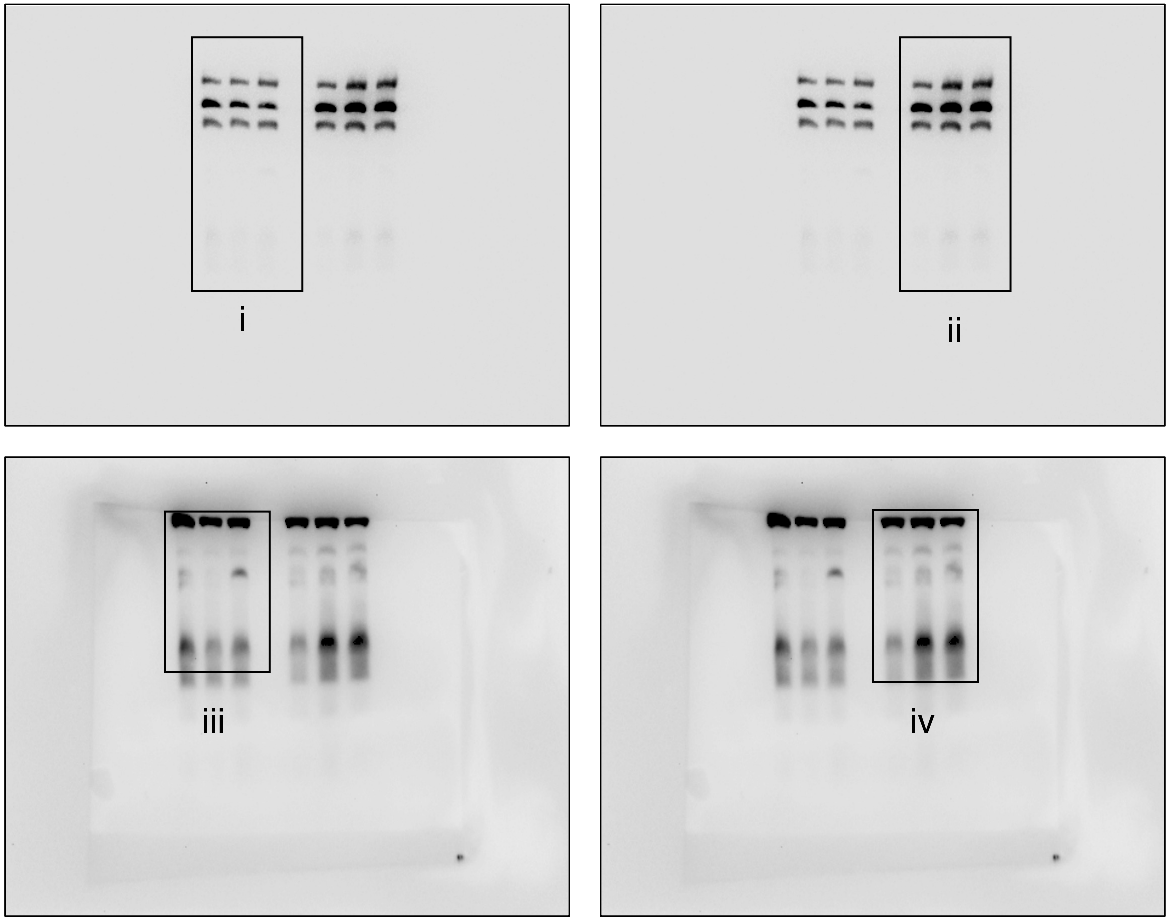

i
ii
iii
iv

Figure S1A

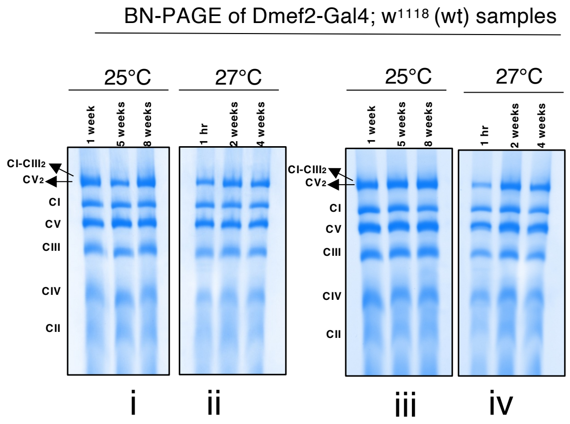

BN-PAGE of Dmef2-Gal4; w1118 (wt) samples
25°C
27°C
25°C
27°C
1 week
5 weeks
8 weeks
1 hr
2 weeks
4 weeks
CI-CIII2
CV2
CI
CV
CIII
CIV
CII
i
ii
iii
iv

Figure S1A, full image

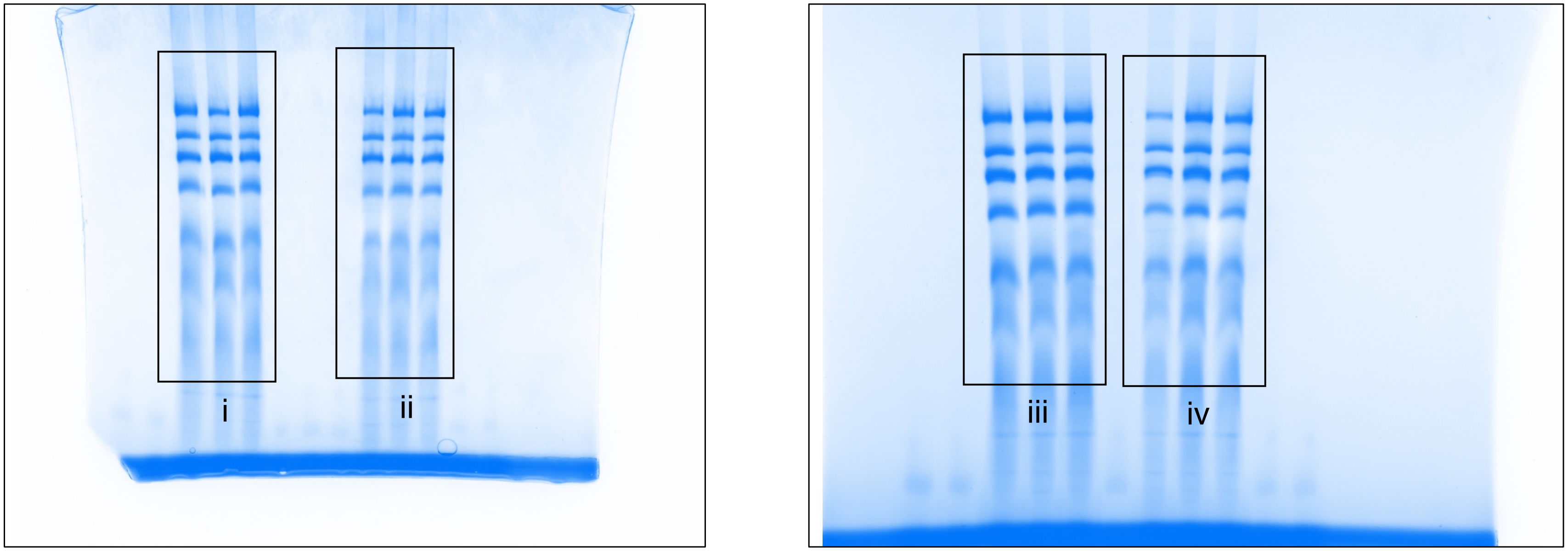

Figure S1B

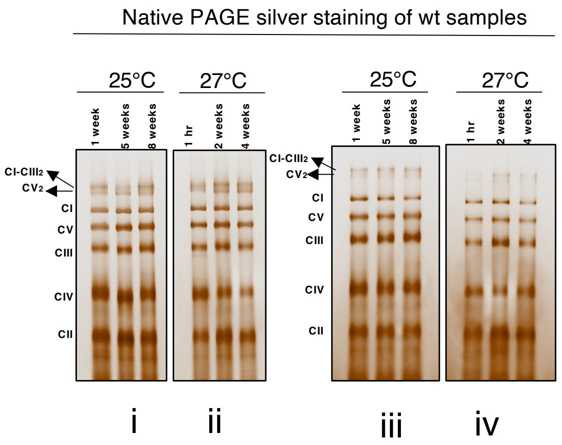

Native PAGE silver staining of wt samples
25°C
27°C
25°C
27°C
1 week
5 weeks
8 weeks
1 hr
2 weeks
4 weeks
1 week
5 weeks
8 weeks
1 hr
2 weeks
4 weeks
CI-CIII2
CV2
CI
CV
CIII
CIV
CII
CI-CIII2
CV2
CI
CV
CIII
CIV
CII
i
ii
iii
iv

Figure S1B, full image

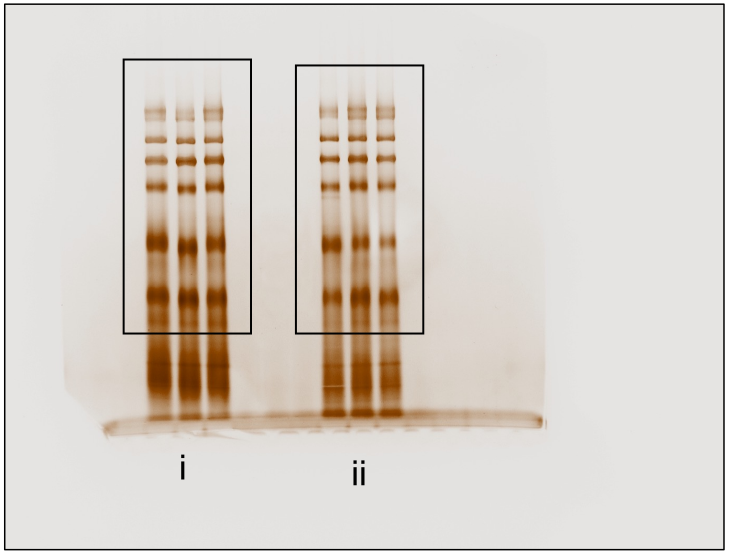

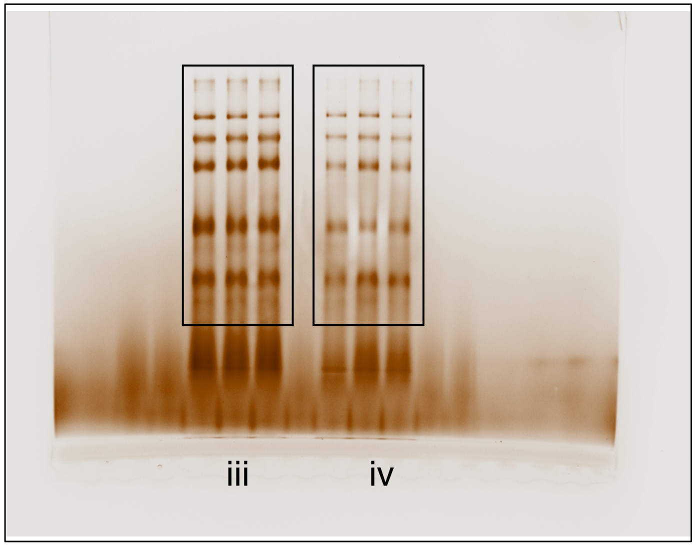

Figure S1C

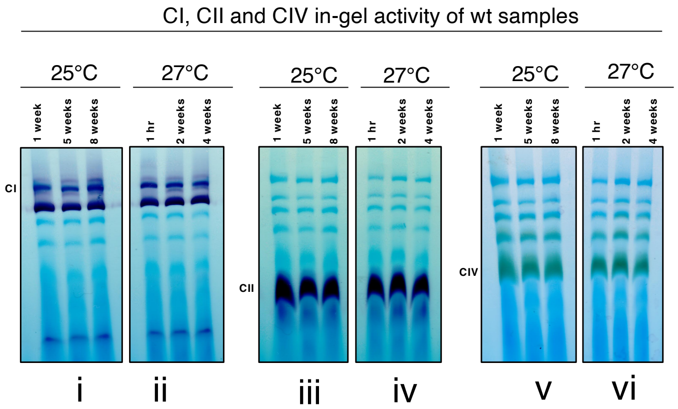

CI, CII and CIV in-gel activity of wt samples
25°C
27°C
25°C
27°C
25°C
27°C
1 week
5 weeks
8 weeks
1 hr
2 weeks
4 weeks
1 week
5 weeks
8 weeks
1 hr
2 weeks
4 weeks
1 week
5 weeks
8 weeks
1 hr
2 weeks
4 weeks
CI
CII
CIV
i
ii
iii
iv
v
vi

Figure S1C, full image

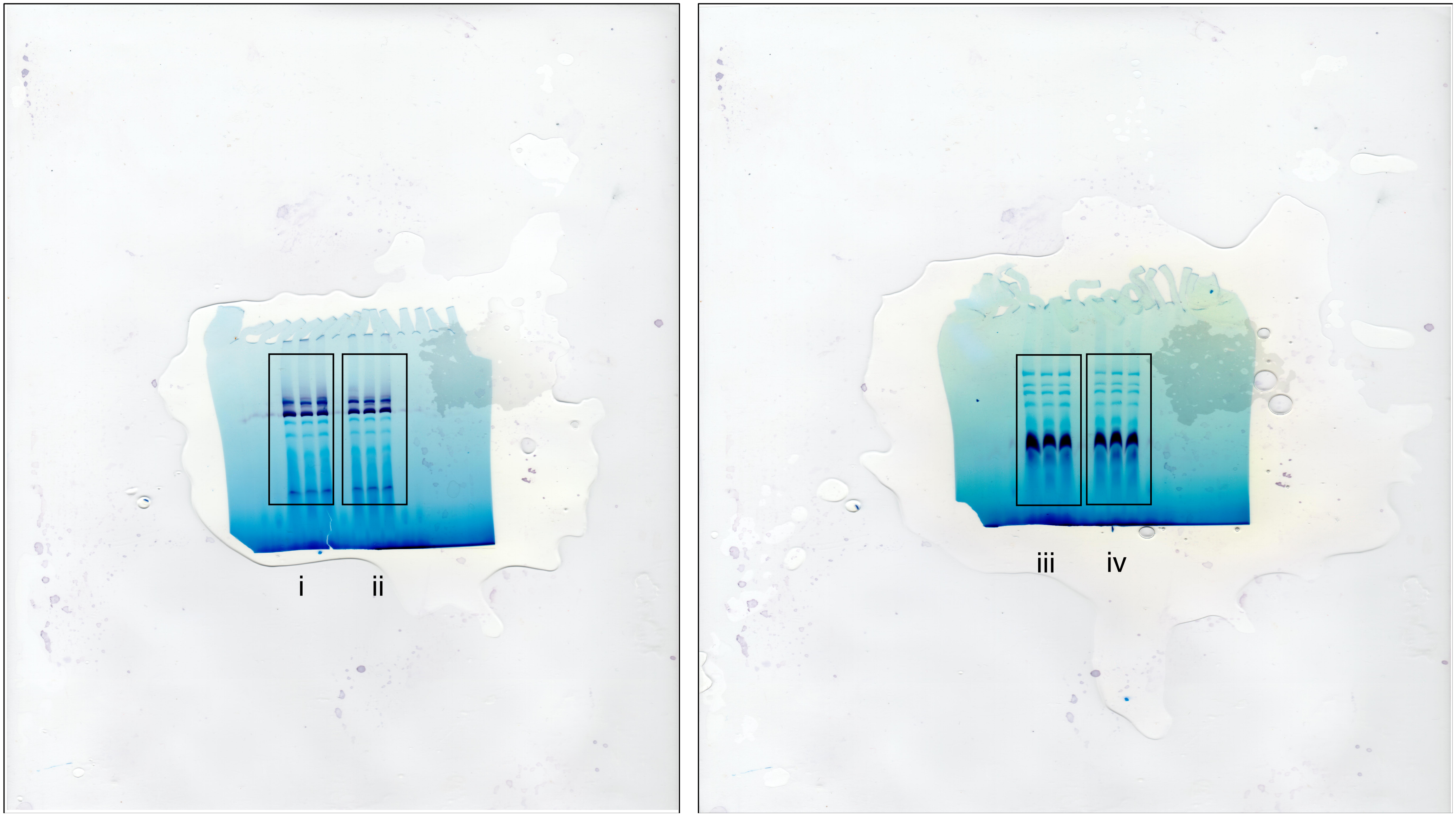

Figure S1C, full image

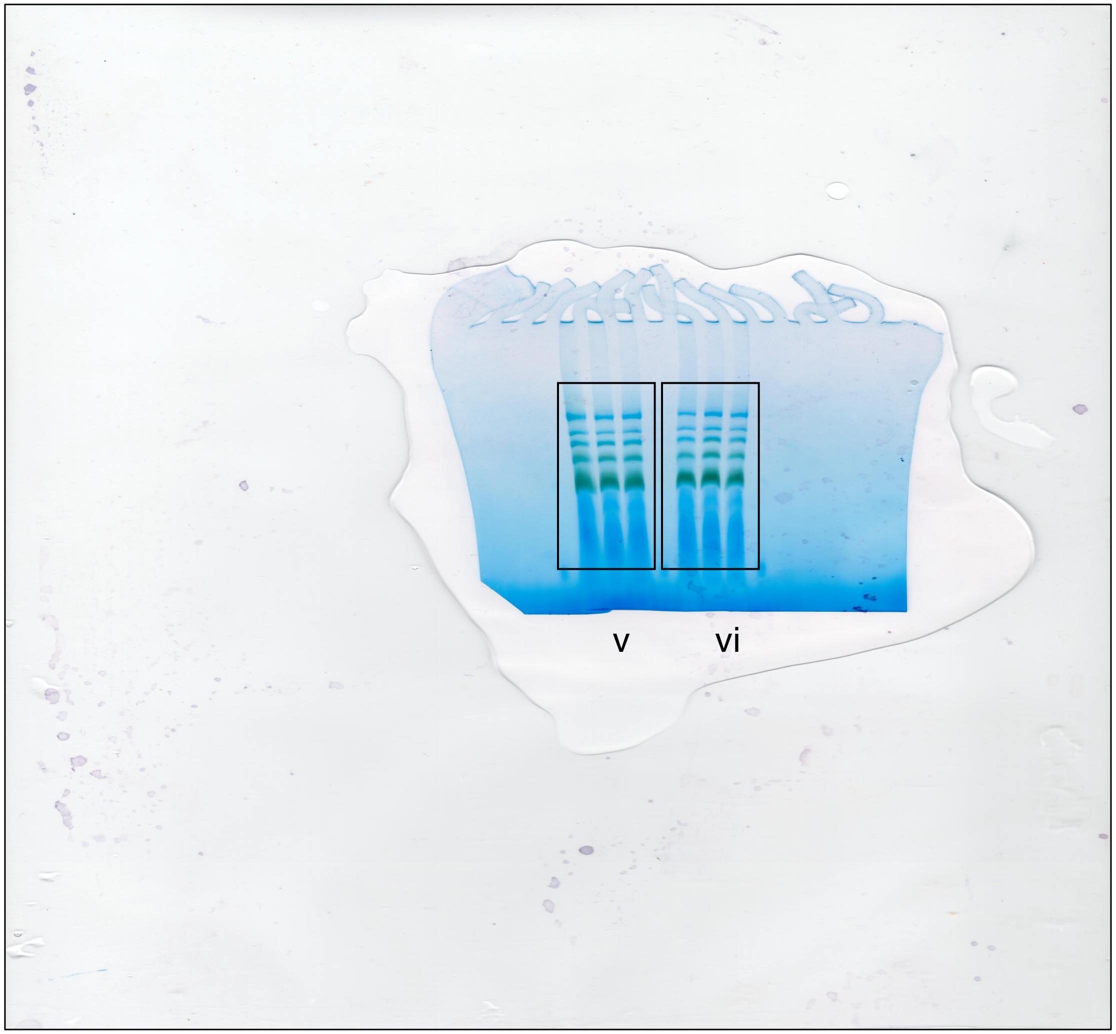

Figure S2B

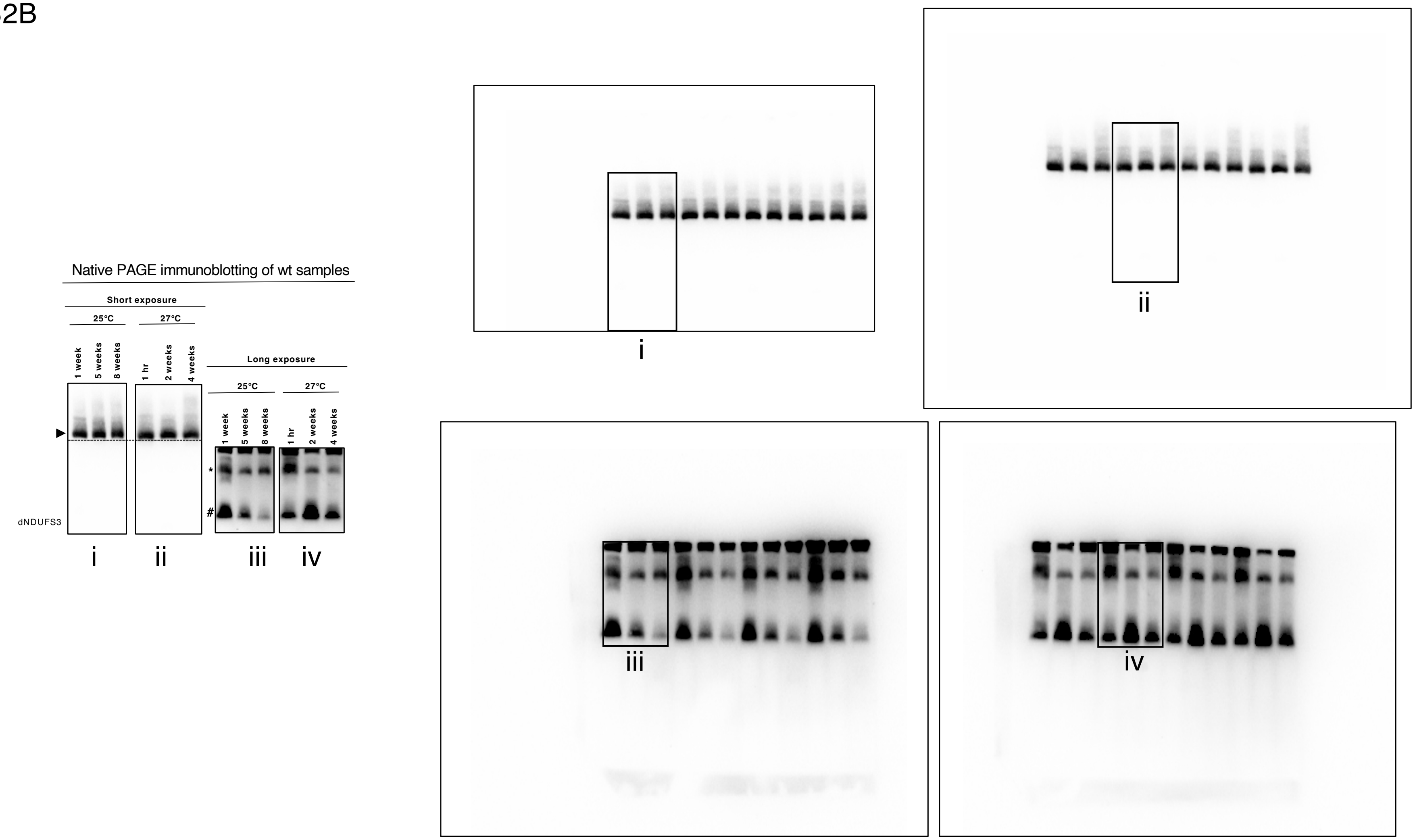

Native PAGE immunoblotting of wt samples
Short exposure
25°C
27°C
1 week
5 weeks
8 weeks
1 hr
2 weeks
4 weeks
Long exposure
25°C
27°C
1 week
5 weeks
8 weeks
1 hr
2 weeks
4 weeks
*
#
dNDUFS3
i
ii
iii
iv
i
ii
iii
iv

S2B additional exposures

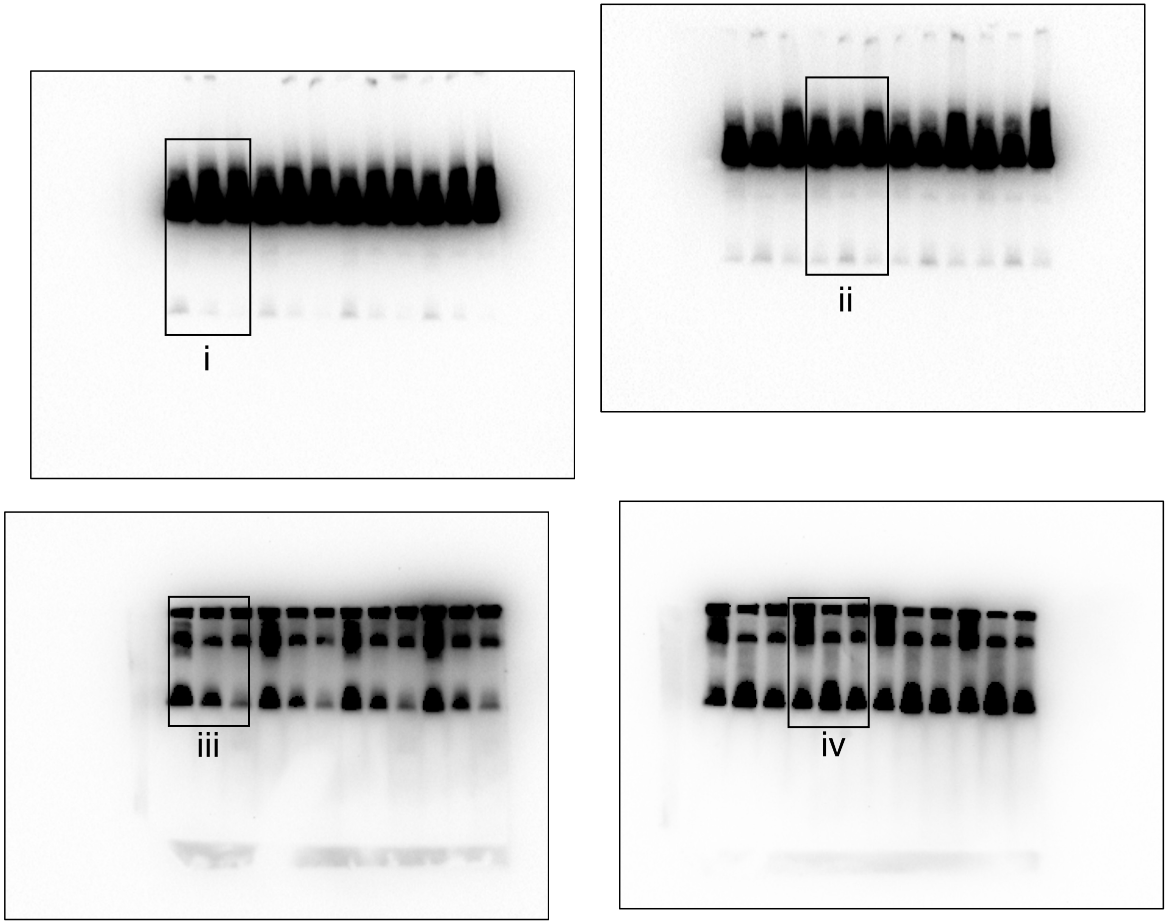

Figure S2C

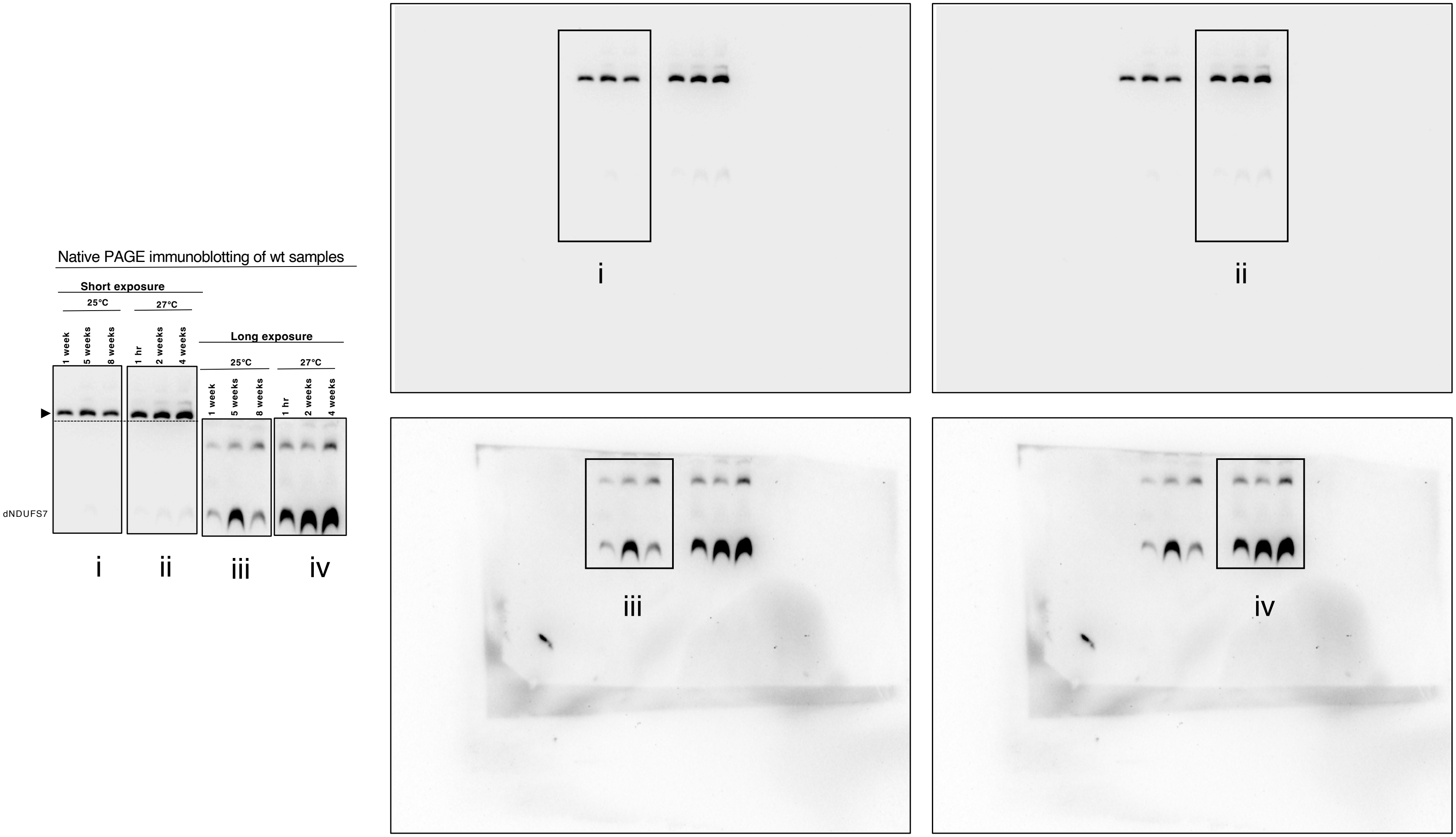

Native PAGE immunoblotting of wt samples
Short exposure
25°C
27°C
1 week
5 weeks
8 weeks
1 hr
2 weeks
4 weeks
Long exposure
25°C
27°C
1 week
5 weeks
8 weeks
1 hr
2 weeks
4 weeks
dNDUFS7
i
ii
iii
iv
i
ii
iii
iv

Figure S2D

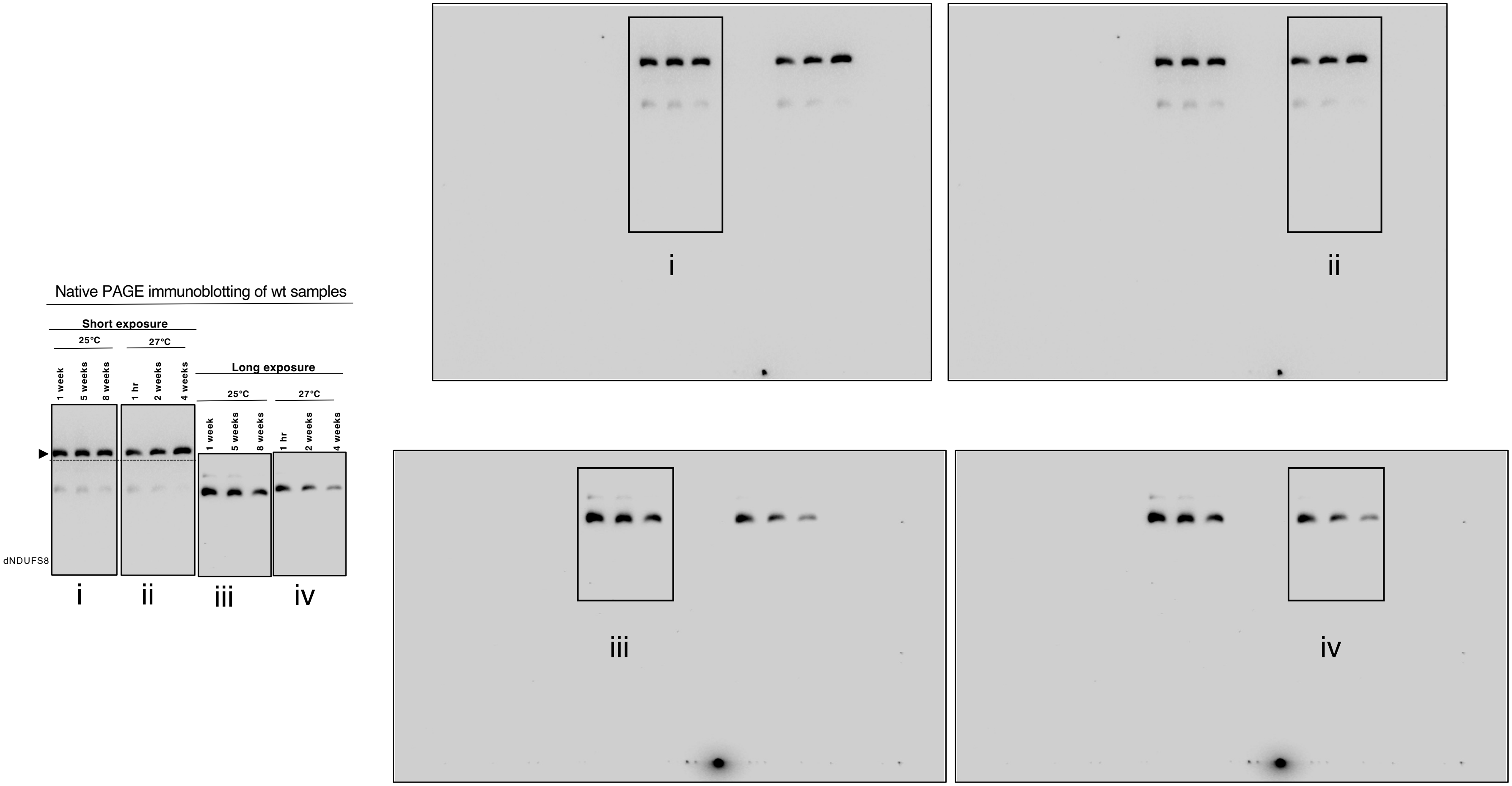

Native PAGE immunoblotting of wt samples
Short exposure
25°C
27°C
1 week
5 weeks
8 weeks
1 hr
2 weeks
4 weeks
Long exposure
25°C
27°C
1 week
5 weeks
8 weeks
1 hr
2 weeks
4 weeks
dNDUFS8
i
ii
iii
iv
i
ii
iii
iv

Figure S2E

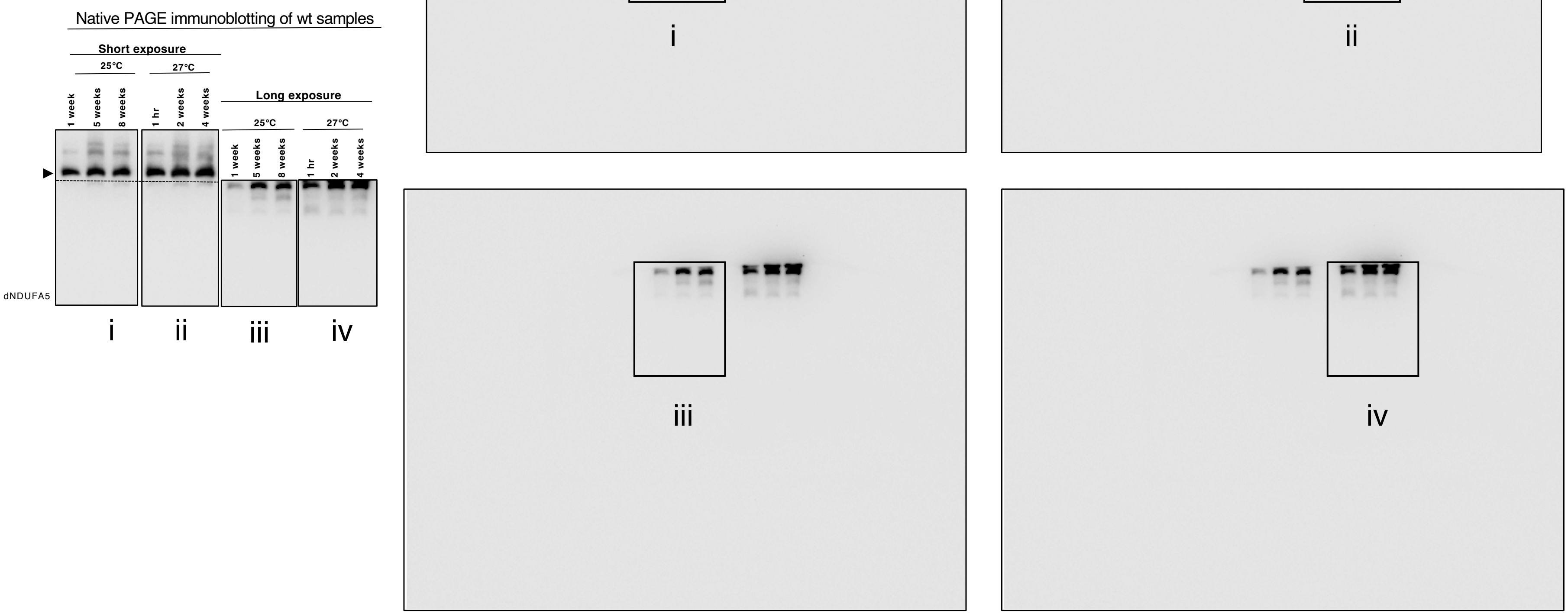

Native PAGE immunoblotting of wt samples
Short exposure
25°C
27°C
1 week
5 weeks
8 weeks
1 hr
2 weeks
4 weeks
Long exposure
25°C
27°C
1 week
5 weeks
8 weeks
1 hr
2 weeks
4 weeks
dNDUFA5
i
ii
iii
iv
i
ii
iii
iv

Figure S2F

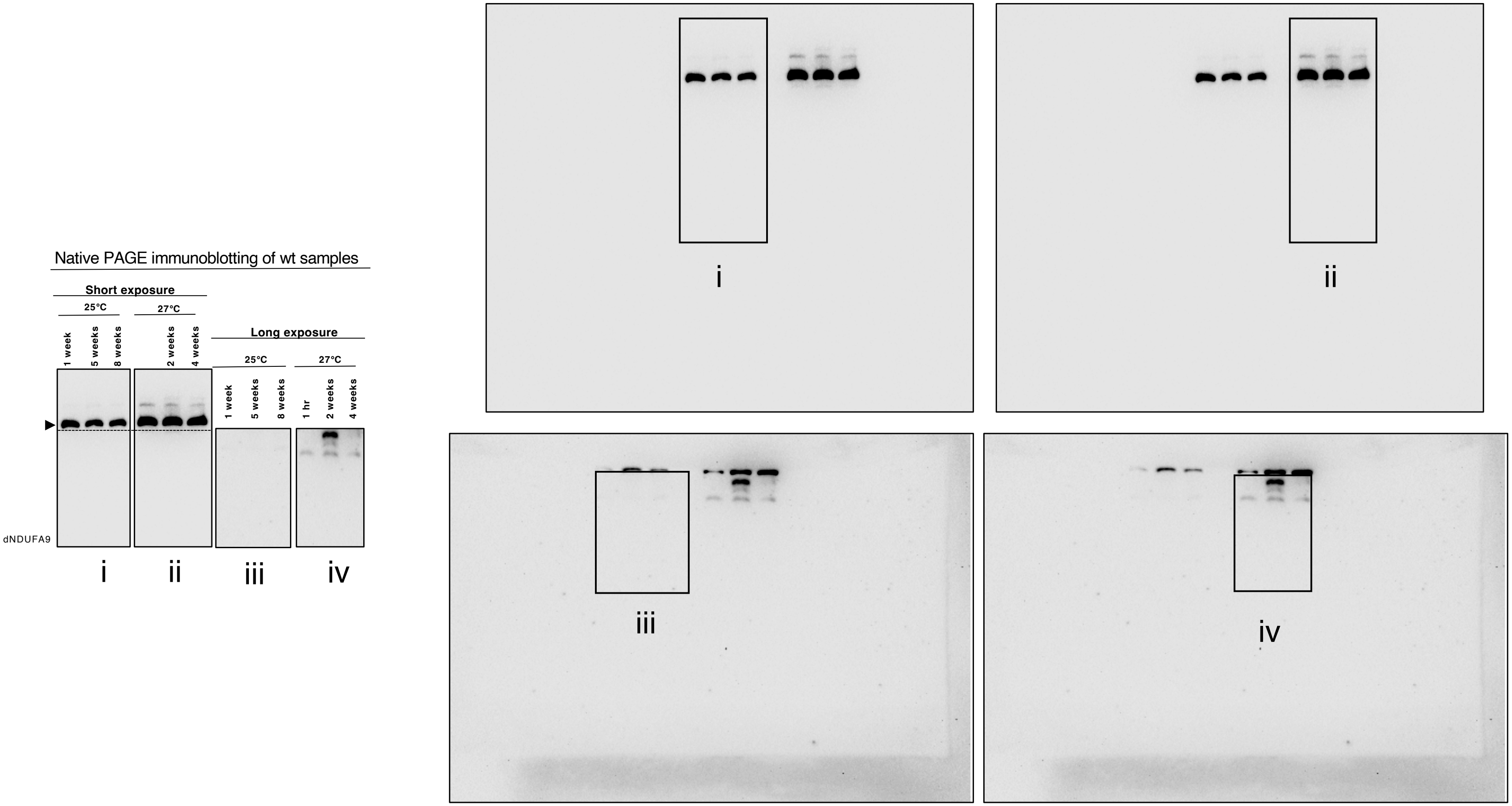
Native PAGE immunoblotting of wt samples
Short exposure
25°C
27°C
1 week
5 weeks
8 weeks
2 weeks
4 weeks
Long exposure
25°C
27°C
1 week
5 weeks
8 weeks
1 hr
2 weeks
4 weeks
dNDUFA9
i
ii
iii
iv
i
ii
iii
iv

Figure S3A

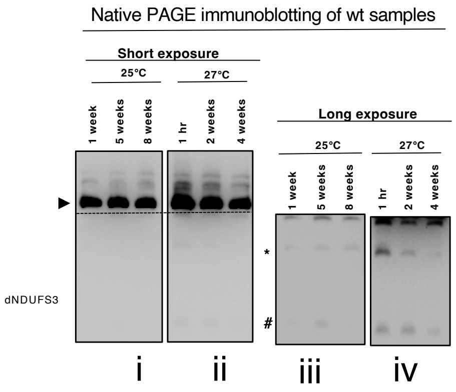
Native PAGE immunoblotting of wt samples
Short exposure
25°C
27°C
1 week
5 weeks
8 weeks
1 hr
2 weeks
4 weeks
Long exposure
25°C
27°C
1 week
5 weeks
8 weeks
1 hr
2 weeks
4 weeks
*
#
dNDUFS3
i
ii
iii
iv

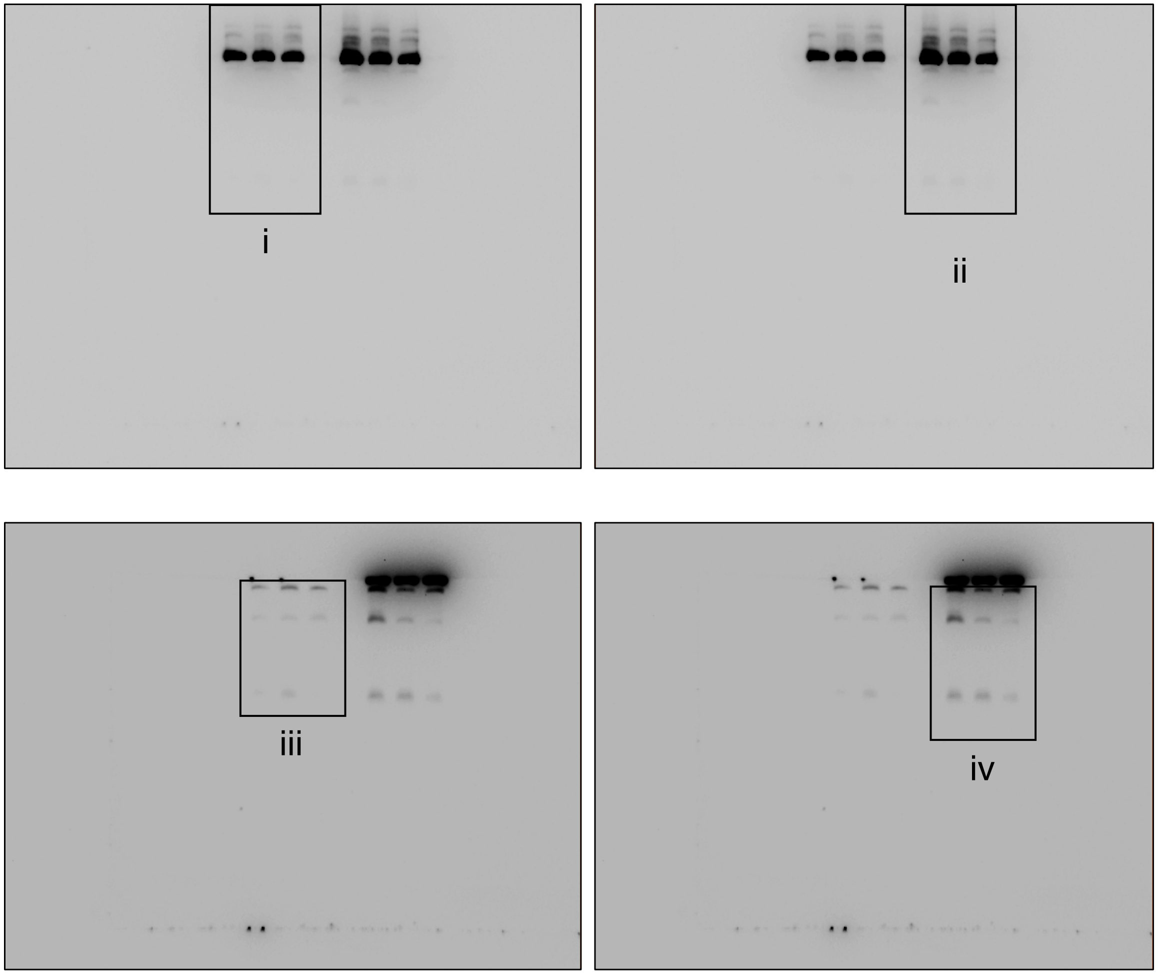
i
ii
iii
iv

Figure S3B

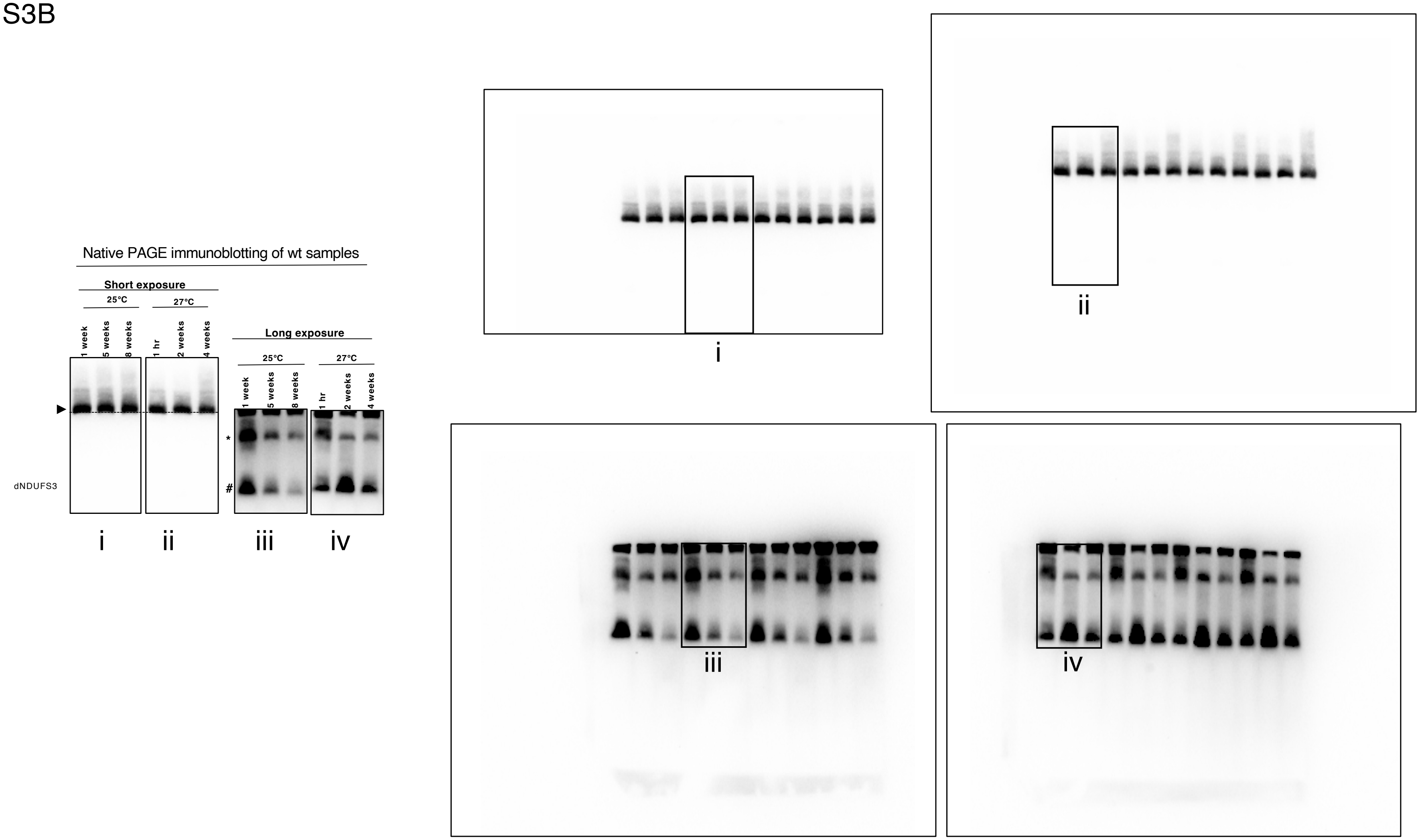

Native PAGE immunoblotting of wt samples
Short exposure
25°C
27°C
1 week
5 weeks
8 weeks
1 hr
2 weeks
4 weeks
Long exposure
25°C
27°C
1 week
5 weeks
8 weeks
1 hr
2 weeks
4 weeks
*
#
dNDUFS3
i
ii
iii
iv
i
ii
iii
iv

S3B additional exposures

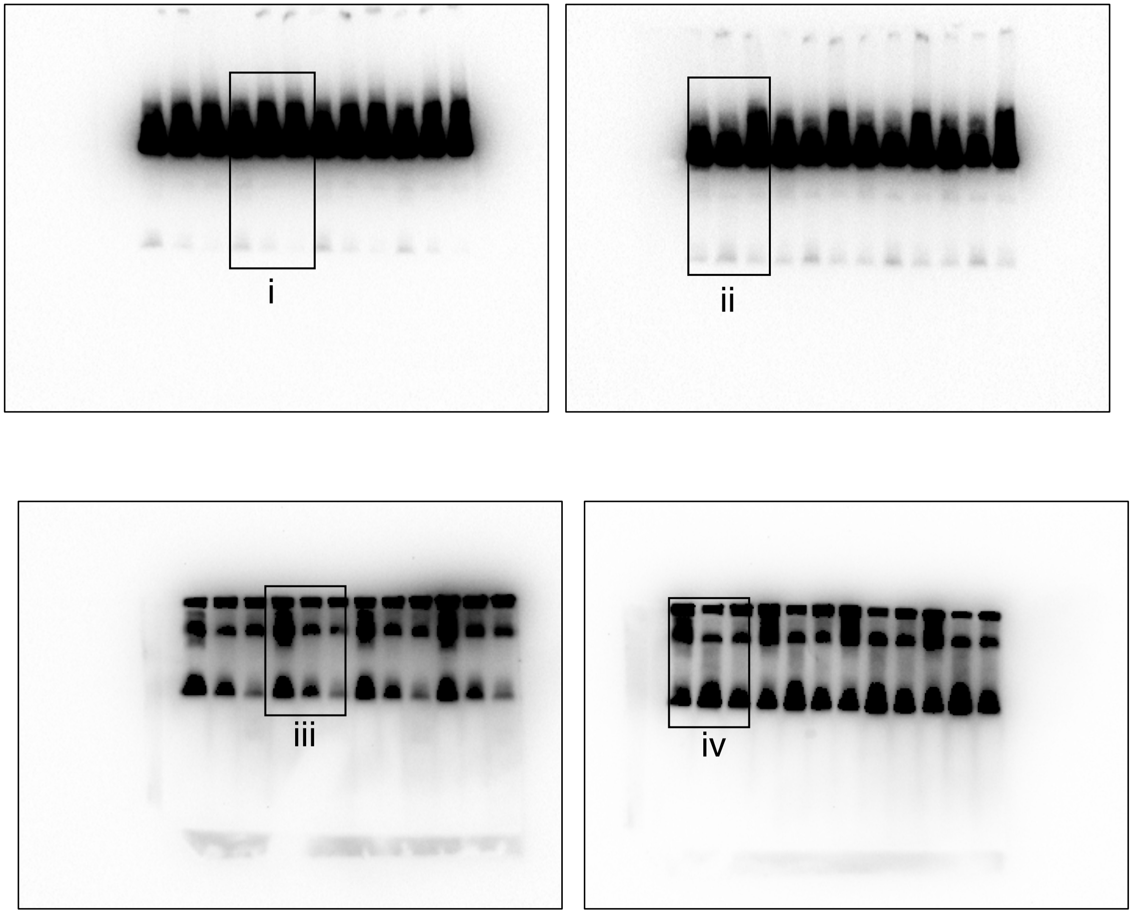

Figure S3C

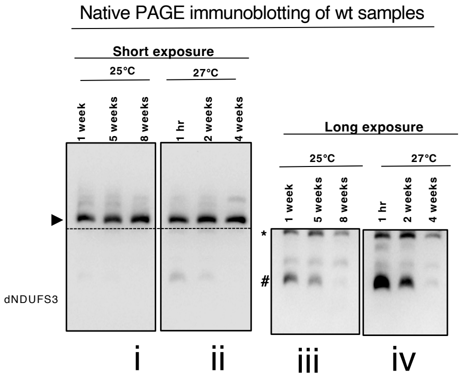

Native PAGE immunoblotting of wt samples
Short exposure
25°C
27°C
1 week
5 weeks
8 weeks
1 hr
2 weeks
4 weeks
Long exposure
25°C
27°C
1 week
5 weeks
8 weeks
1 hr
2 weeks
4 weeks
*
#
dNDUFS3
i
ii
iii
iv

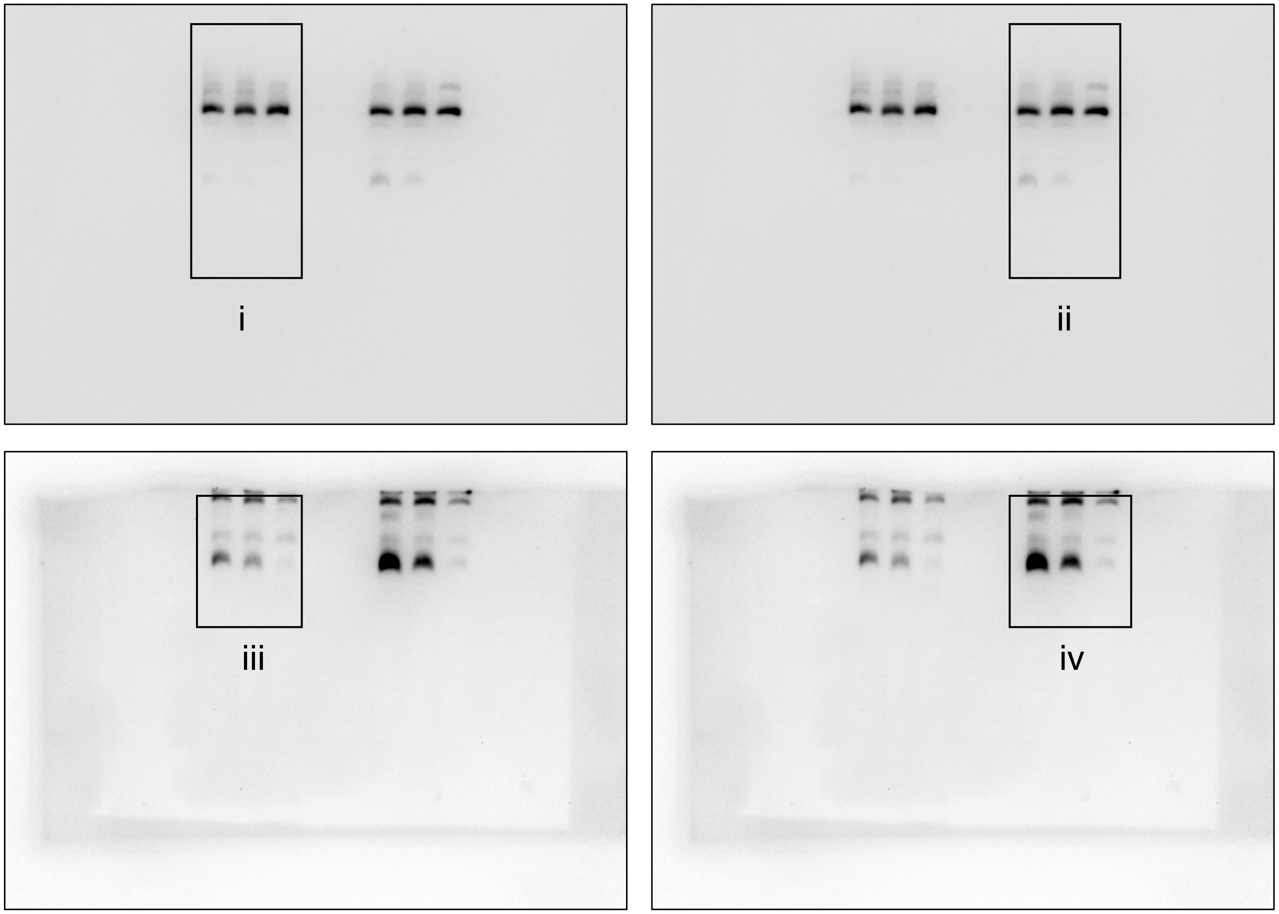

i
ii
iii
iv

Figure S3D

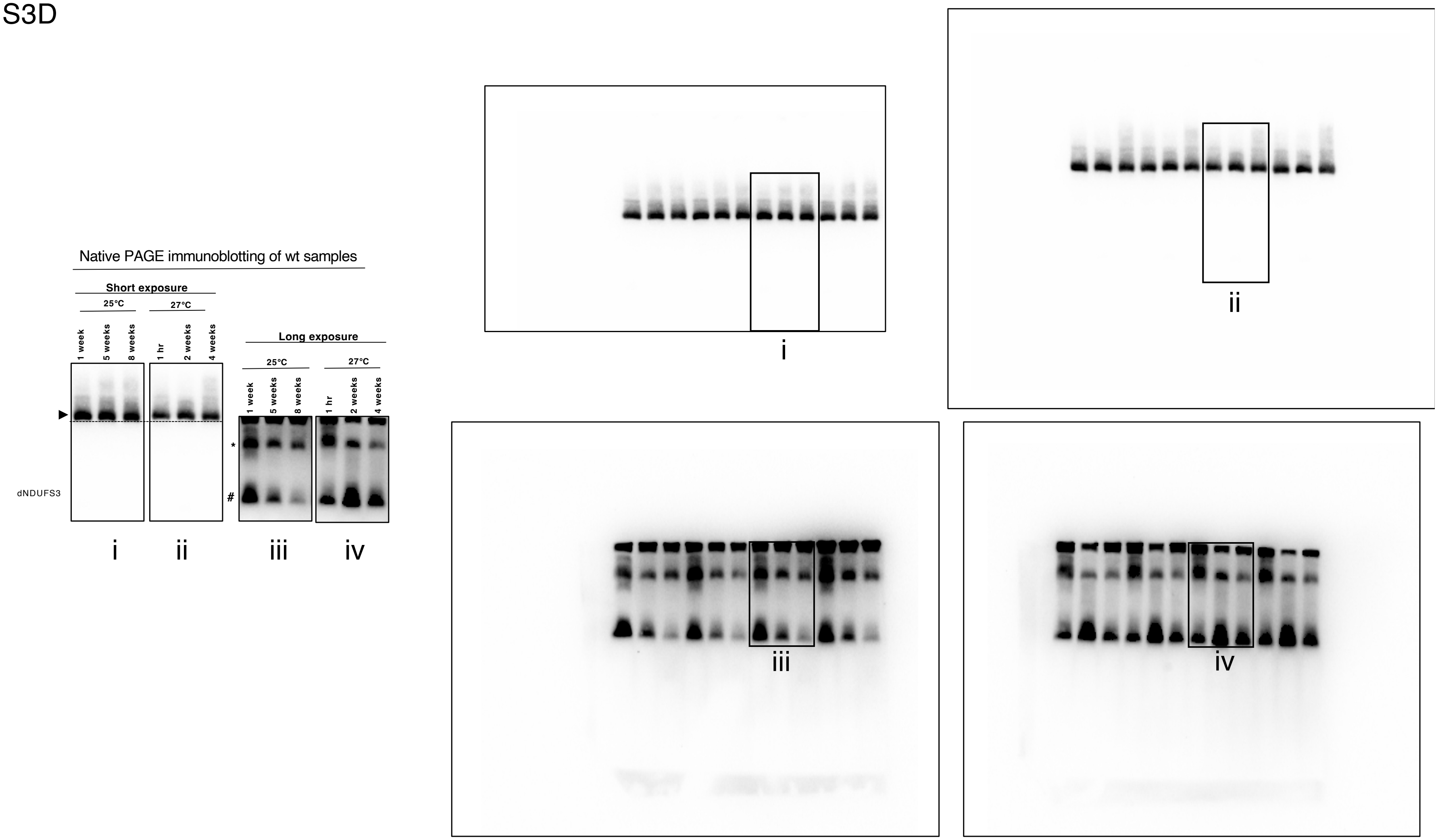

Native PAGE immunoblotting of wt samples
Short exposure
25°C
27°C
1 week
5 weeks
8 weeks
1 hr
2 weeks
4 weeks
Long exposure
25°C
27°C
1 week
5 weeks
8 weeks
1 hr
2 weeks
4 weeks
*
#
dNDUFS3
i
ii
iii
iv
i
ii
iii
iv

S3D additional exposures

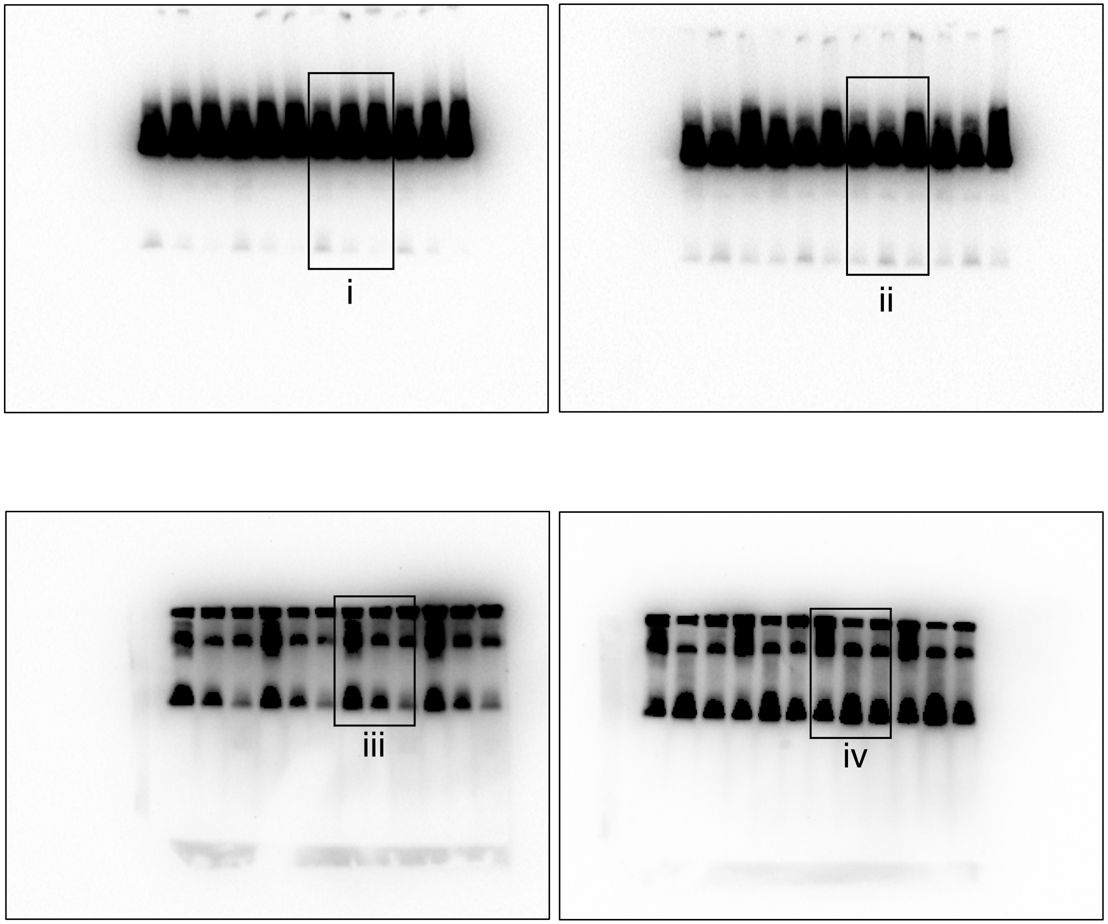

Figure S4B

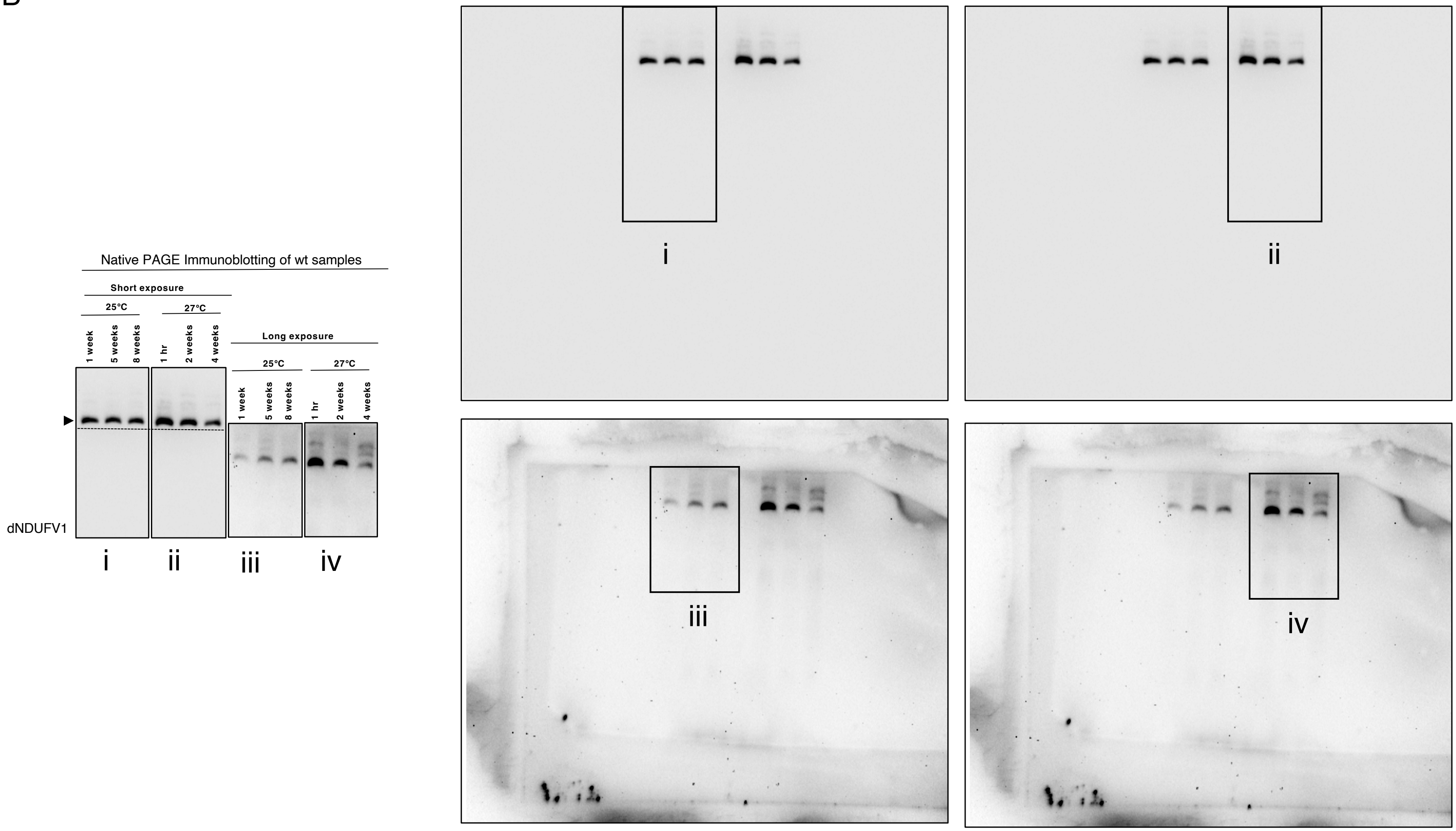

Native PAGE Immunoblotting of wt samples
Short exposure
25°C
27°C
1 week
5 weeks
8 weeks
1 hr
2 weeks
4 weeks
Long exposure
25°C
27°C
1 week
5 weeks
8 weeks
1 hr
2 weeks
4 weeks
dNDUFV1
i
ii
iii
iv
i
ii
iii
iv

Figure S4C

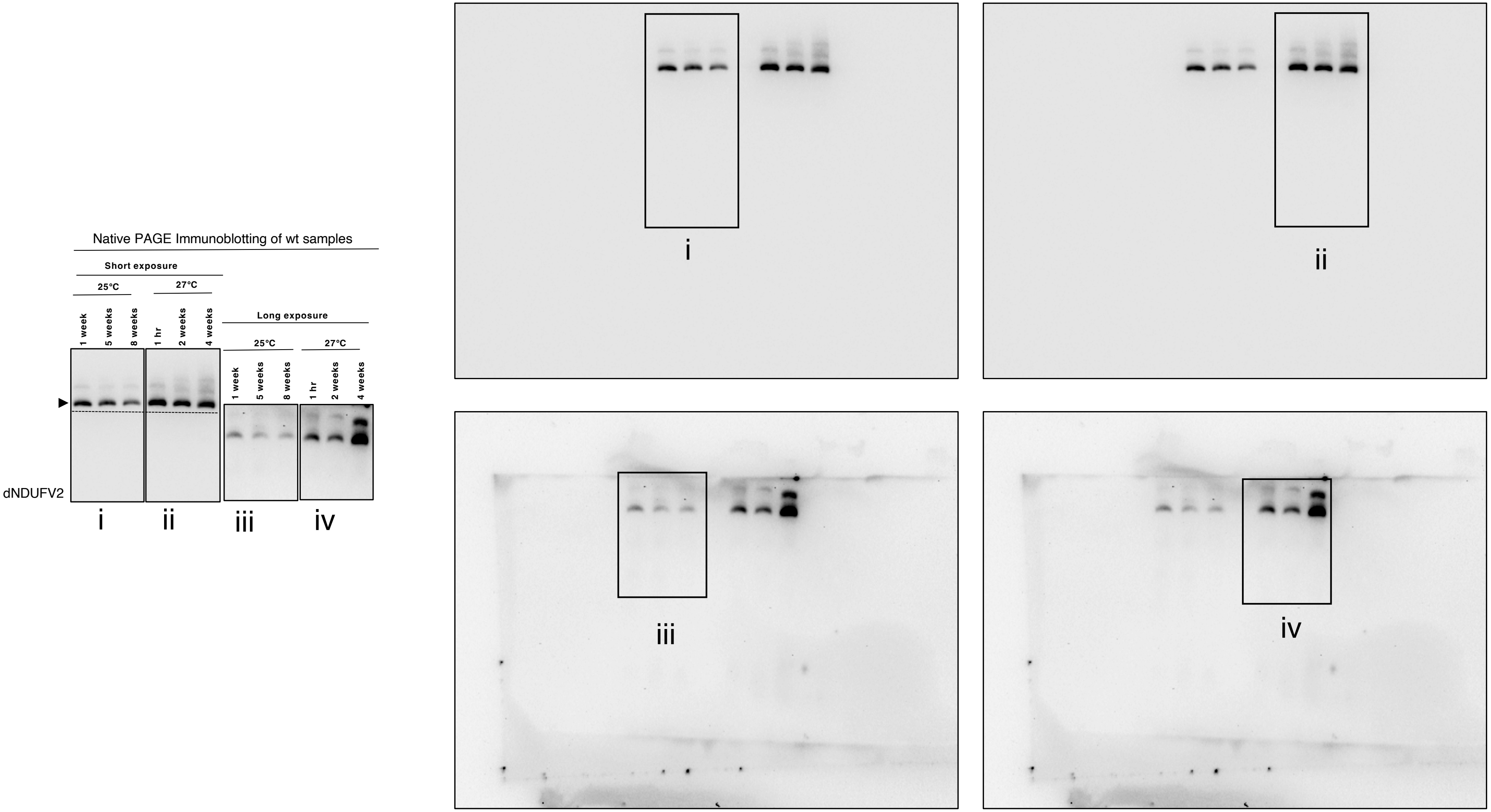

Native PAGE Immunoblotting of wt samples
Short exposure
25°C
27°C
1 week
5 weeks
8 weeks
1 hr
2 weeks
4 weeks
Long exposure
25°C
27°C
1 week
5 weeks
8 weeks
1 hr
2 weeks
4 weeks
dNDUFV2
i
ii
iii
iv
i
ii
iii
iv

Figure S4D

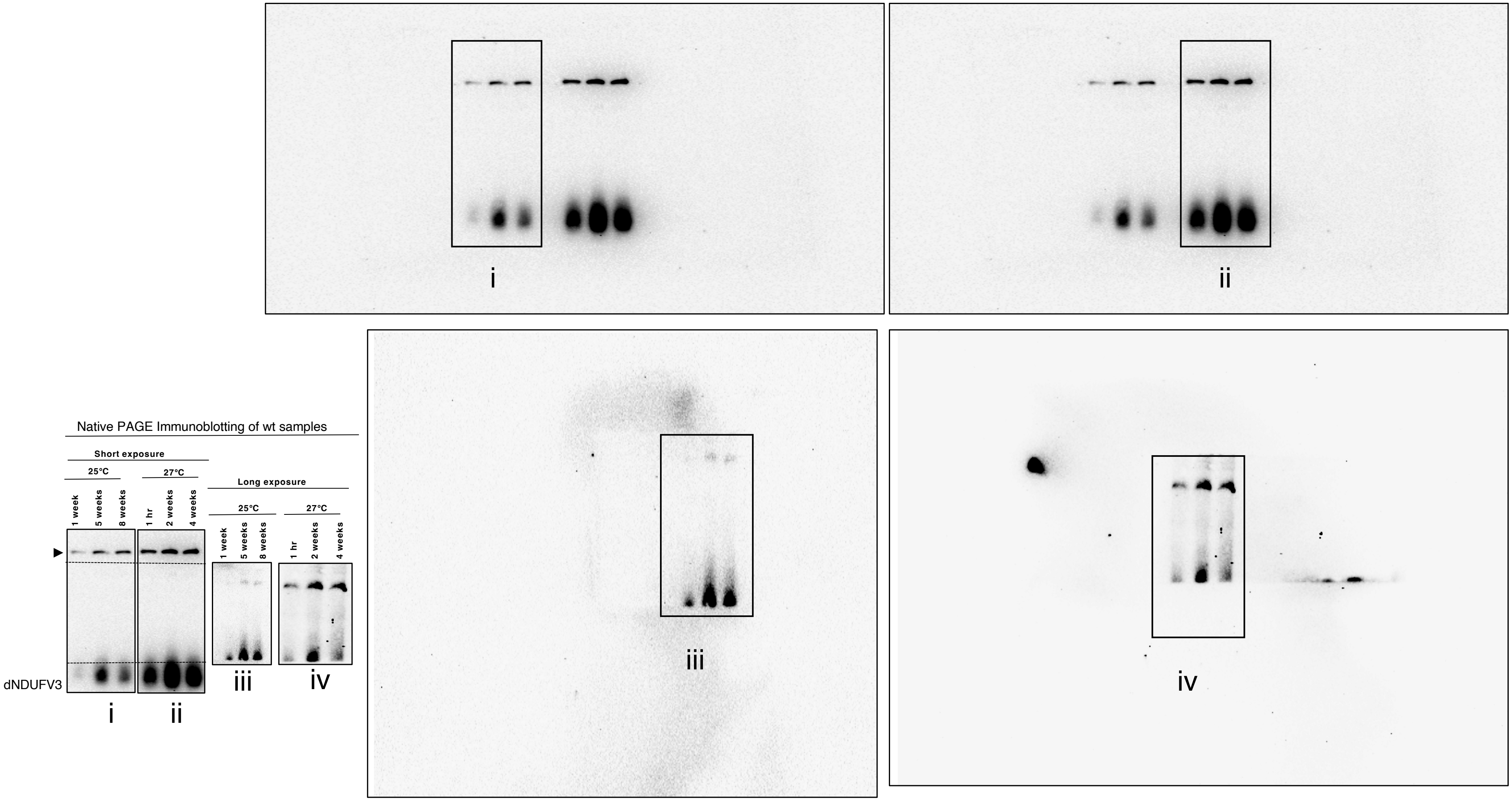

i
ii
Native PAGE Immunoblotting of wt samples
Short exposure
25°C
27°C
1 week
5 weeks
8 weeks
1 hr
2 weeks
4 weeks
Long exposure
25°C
27°C
1 week
5 weeks
8 weeks
1 hr
2 weeks
4 weeks
dNDUFV3
i
ii
iii
iv
iii
iv

Figure S4E

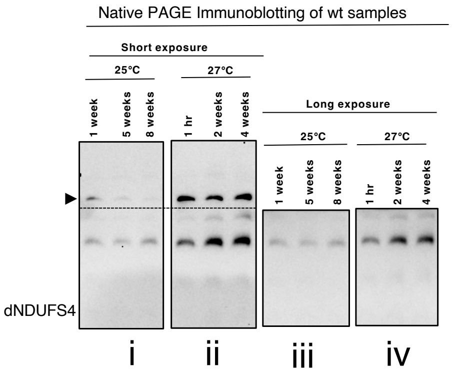

Native PAGE Immunoblotting of wt samples
Short exposure
25°C
27°C
1 week
5 weeks
8 weeks
1 hr
2 weeks
4 weeks
Long exposure
25°C
27°C
1 week
5 weeks
8 weeks
1 hr
2 weeks
4 weeks
dNDUFS4
i
ii
iii
iv

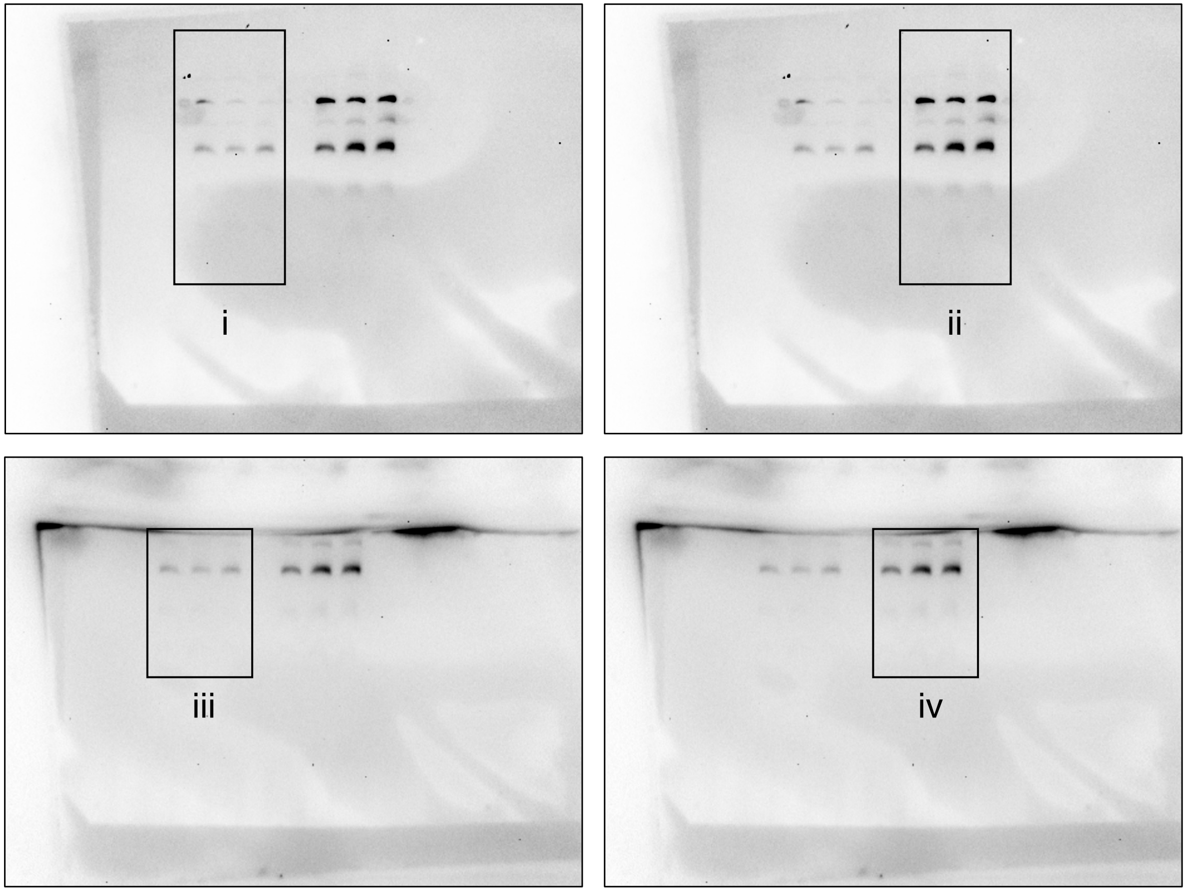

i
ii
iii
iv

Figure S4F

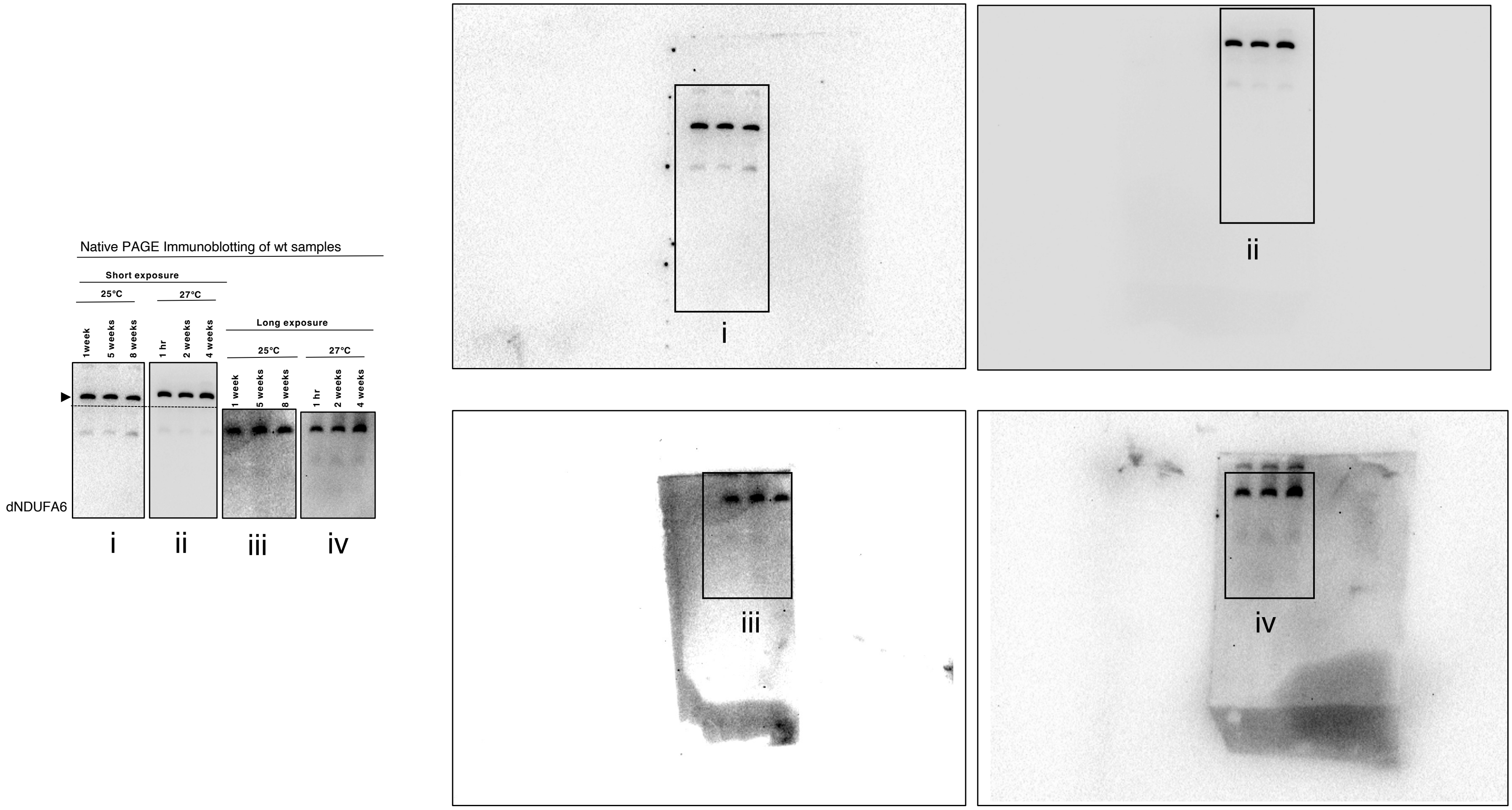

Native PAGE Immunoblotting of wt samples
Short exposure
25°C
27°C
1week
5 weeks
8 weeks
1 hr
2 weeks
4 weeks
Long exposure
25°C
27°C
1 week
5 weeks
8 weeks
1 hr
2 weeks
4 weeks
dNDUFA6
i
ii
iii
iv
i
ii
iii
iv

Figure S4G

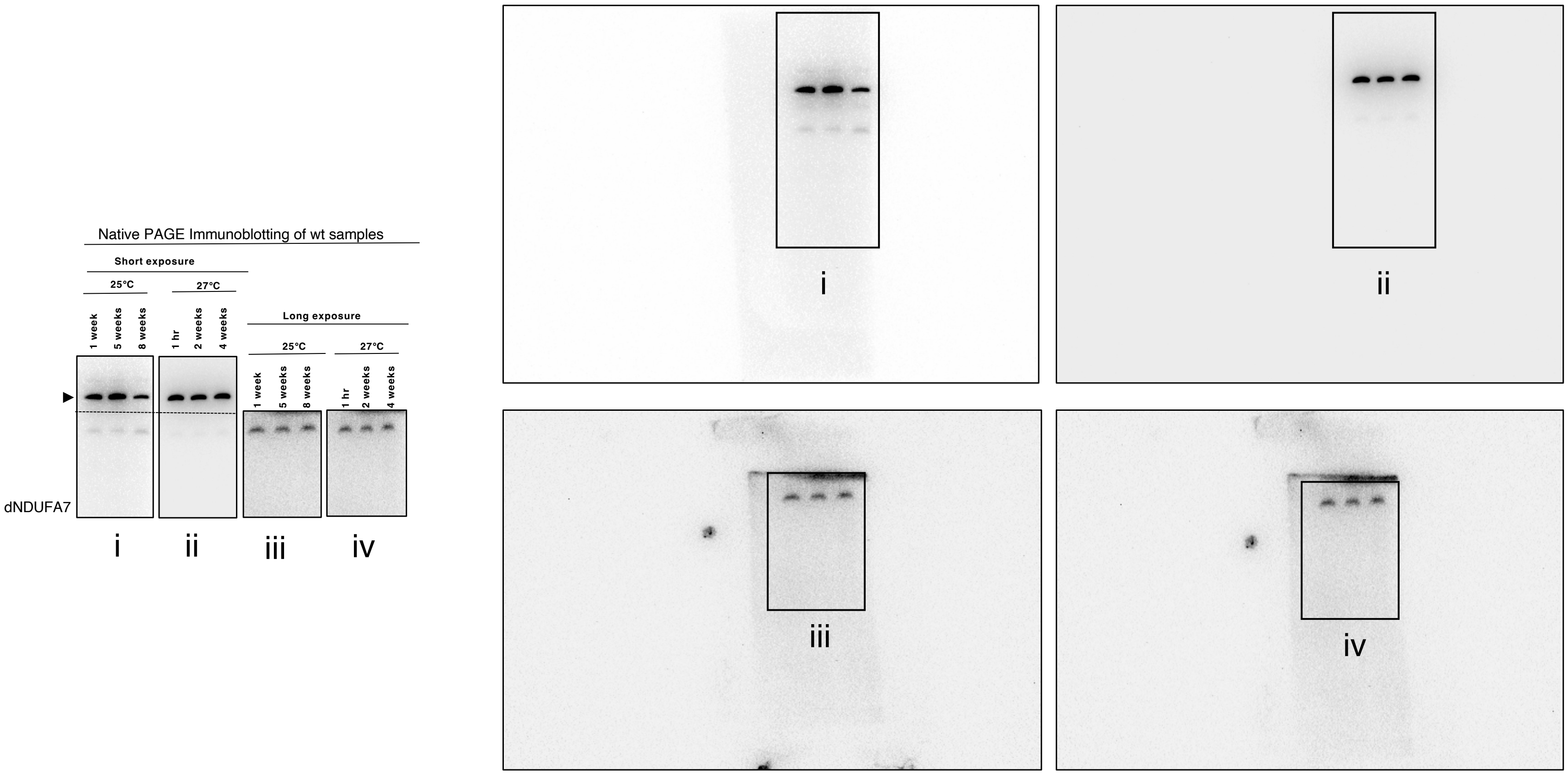
Native PAGE Immunoblotting of wt samples
Short exposure
25°C
27°C
1 week
5 weeks
8 weeks
1 hr
2 weeks
4 weeks
Long exposure
25°C
27°C
1 week
5 weeks
8 weeks
1 hr
2 weeks
4 weeks
dNDUFA7
i
ii
iii
iv
i
ii
iii
iv

Figure S4H

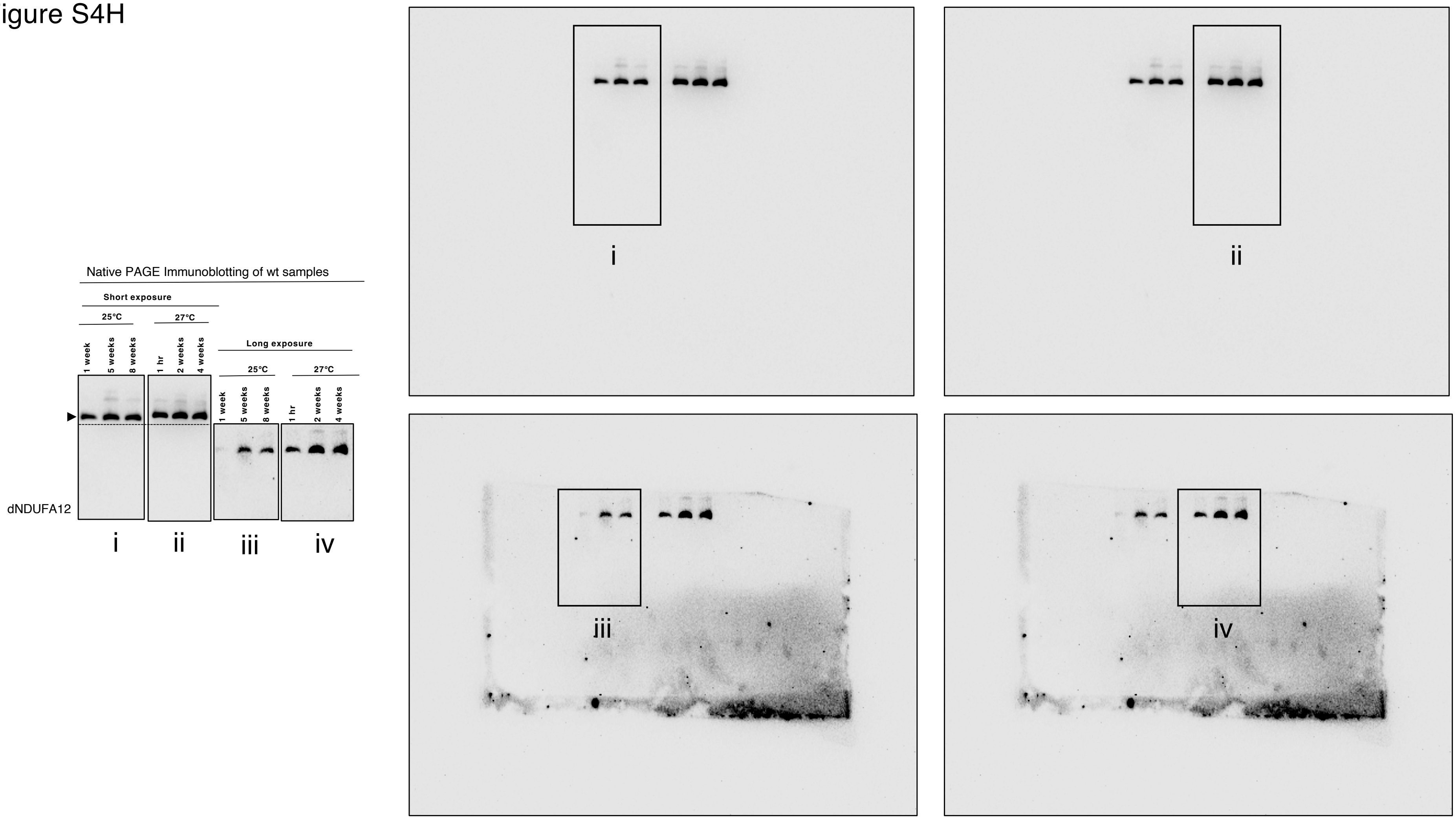
Native PAGE Immunoblotting of wt samples
Short exposure
25°C
27°C
1 week
5 weeks
8 weeks
1 hr
2 weeks
4 weeks
Long exposure
25°C
27°C
1 week
5 weeks
8 weeks
1 hr
2 weeks
4 weeks
dNDUFA12
i
ii
iii
iv
i
ii
iii
iv

Figure S5B

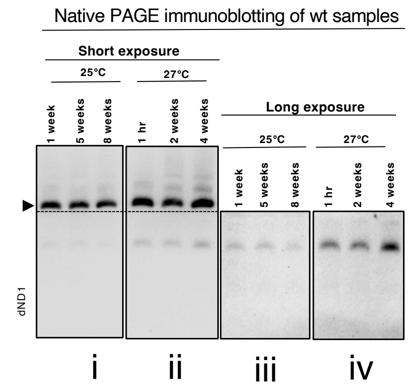

Native PAGE immunoblotting of wt samples
Short exposure
25°C
27°C
1 week
5 weeks
8 weeks
1 hr
2 weeks
4 weeks
Long exposure
25°C
27°C
1 week
5 weeks
8 weeks
1 hr
2 weeks
4 weeks
dND1
i
ii
iii
iv

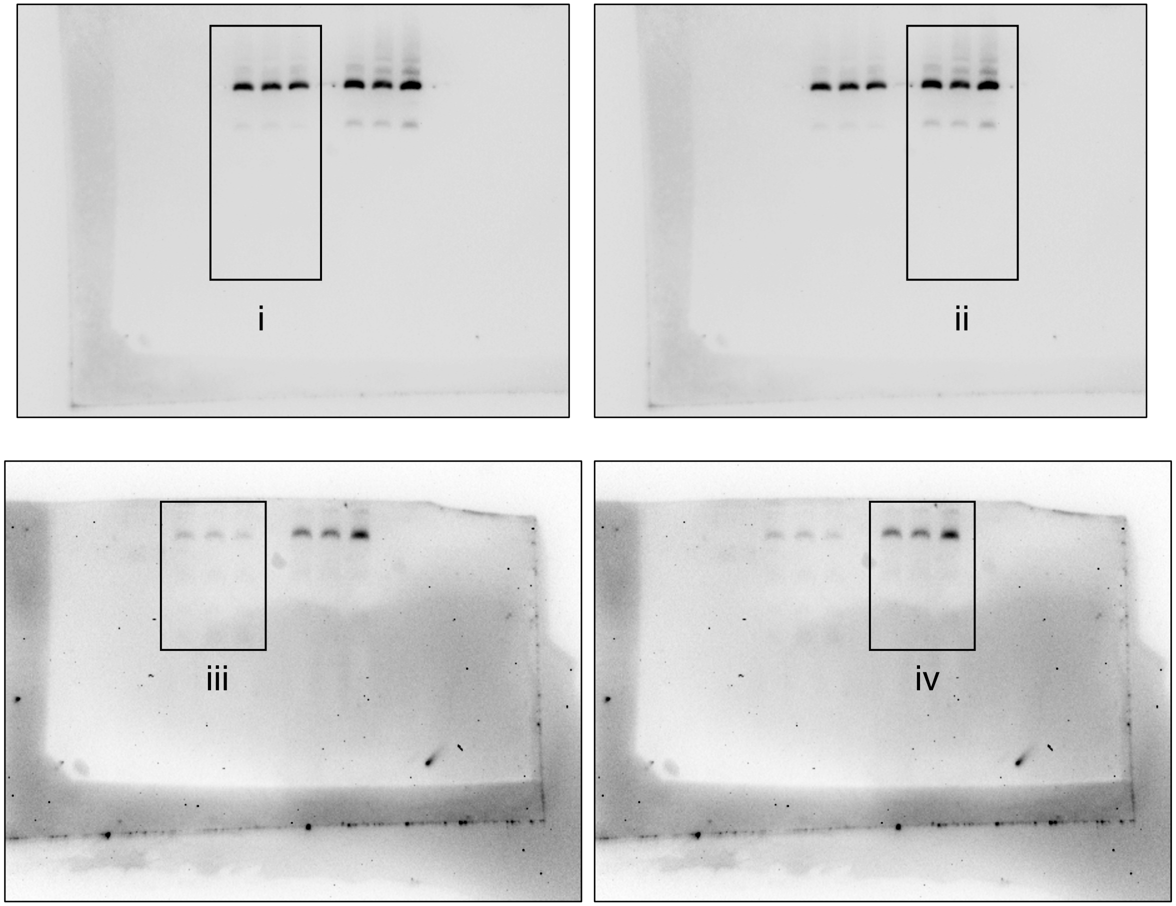

i
ii
iii
iv

Figure S5C

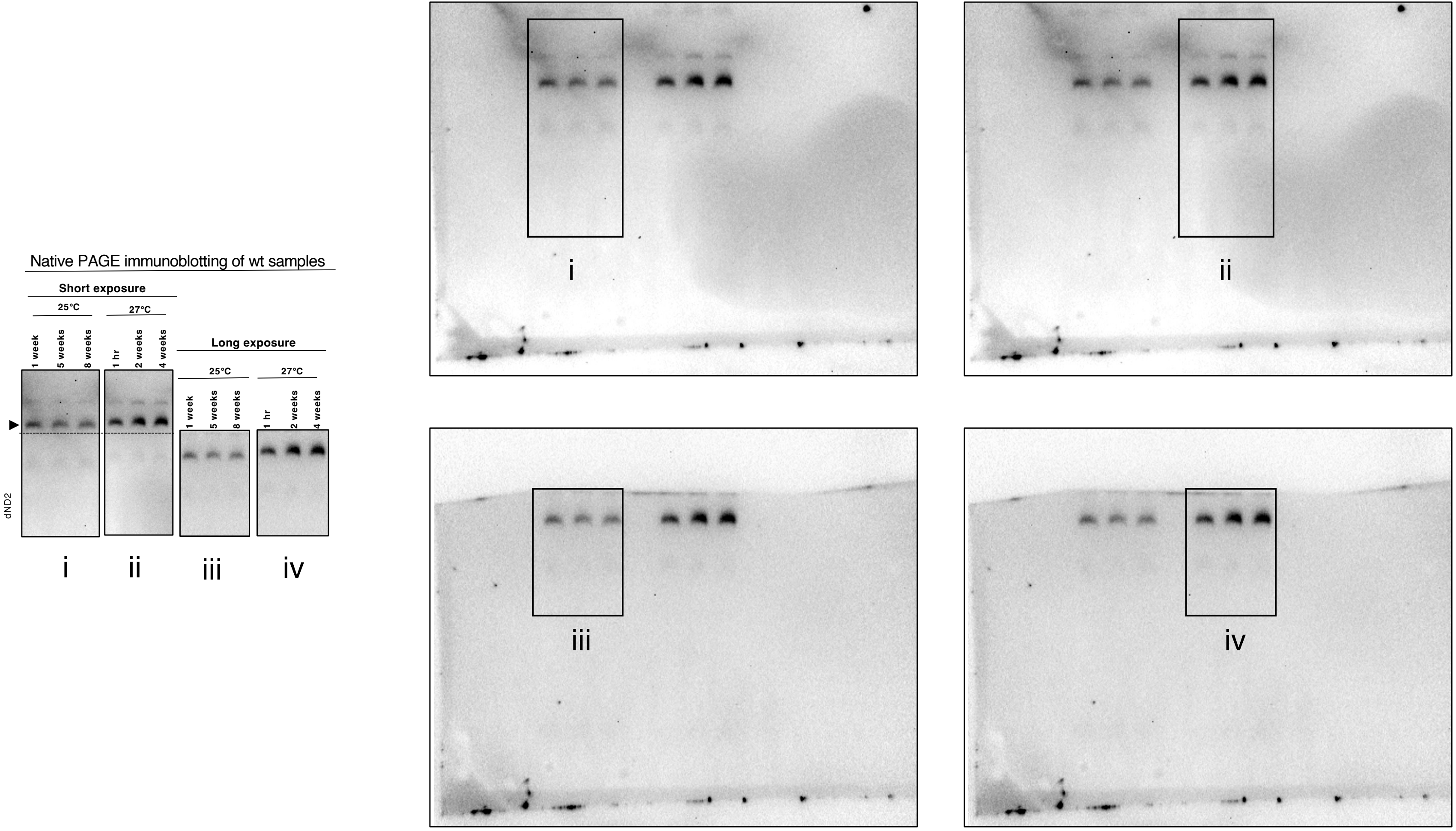

Native PAGE immunoblotting of wt samples
Short exposure
25°C
27°C
1 week
5 weeks
8 weeks
1 hr
2 weeks
4 weeks
Long exposure
25°C
27°C
1 week
5 weeks
8 weeks
1 hr
2 weeks
4 weeks
dND2
i
ii
iii
iv
i
ii
iii
iv

Figure S5D

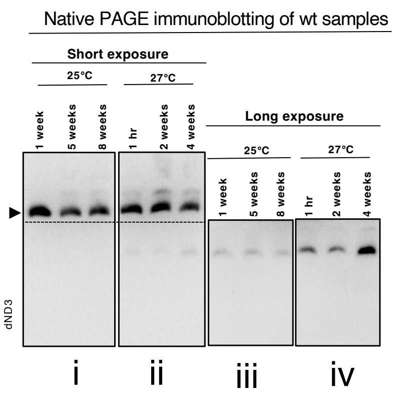
Native PAGE immunoblotting of wt samples
Short exposure
25°C
27°C
1 week
5 weeks
8 weeks
1 hr
2 weeks
4 weeks
Long exposure
25°C
27°C
1 week
5 weeks
8 weeks
1 hr
2 weeks
4 weeks
dND3
i
ii
iii
iv

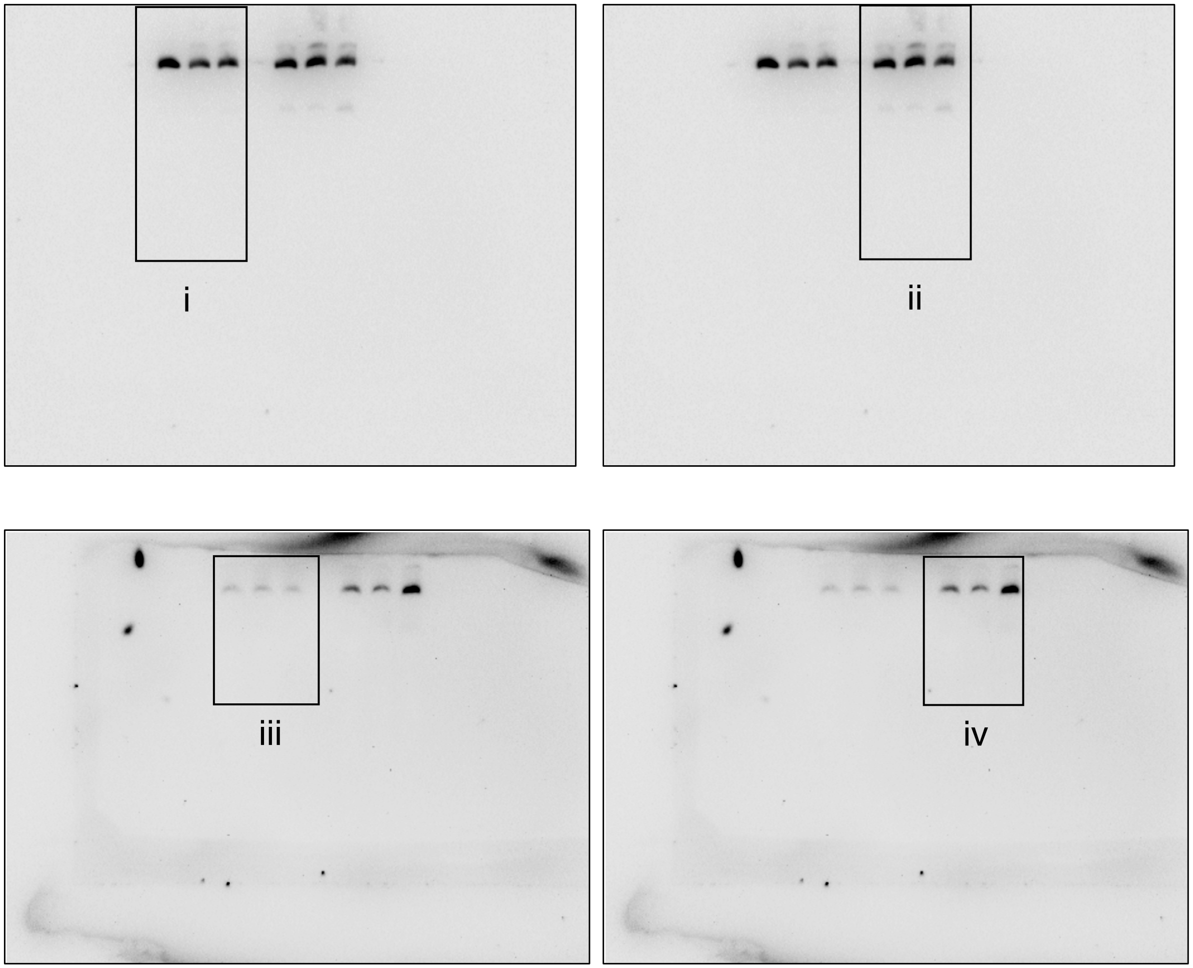
i
ii
iii
iv

Figure S5E

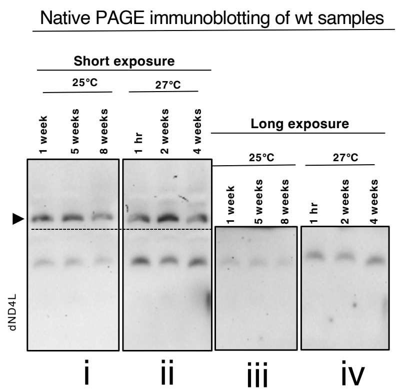
Native PAGE immunoblotting of wt samples
Short exposure
25°C
27°C
1 week
5 weeks
8 weeks
1 hr
2 weeks
4 weeks
Long exposure
25°C
27°C
1 week
5 weeks
8 weeks
1 hr
2 weeks
4 weeks
dND4L
i
ii
iii
iv

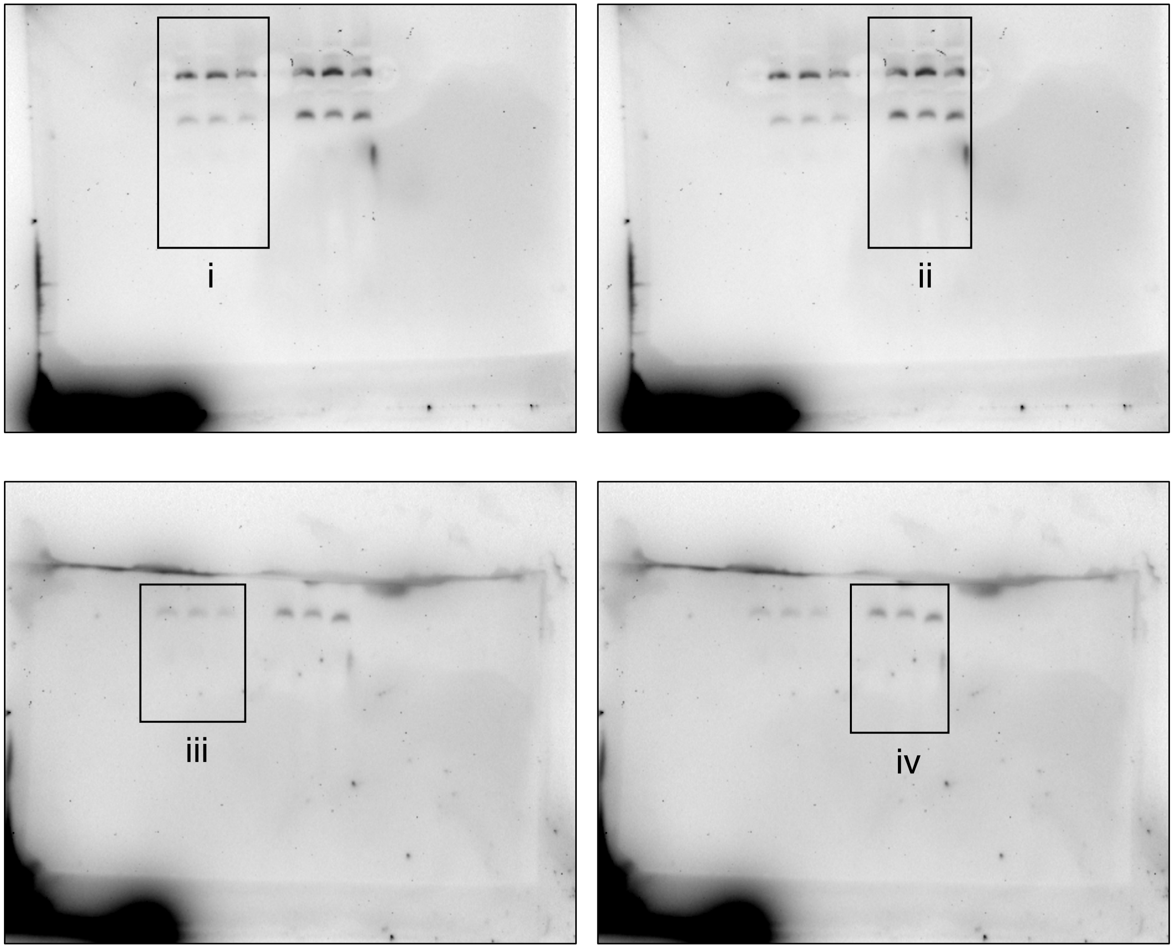
i
ii
iii
iv

Figure S5F

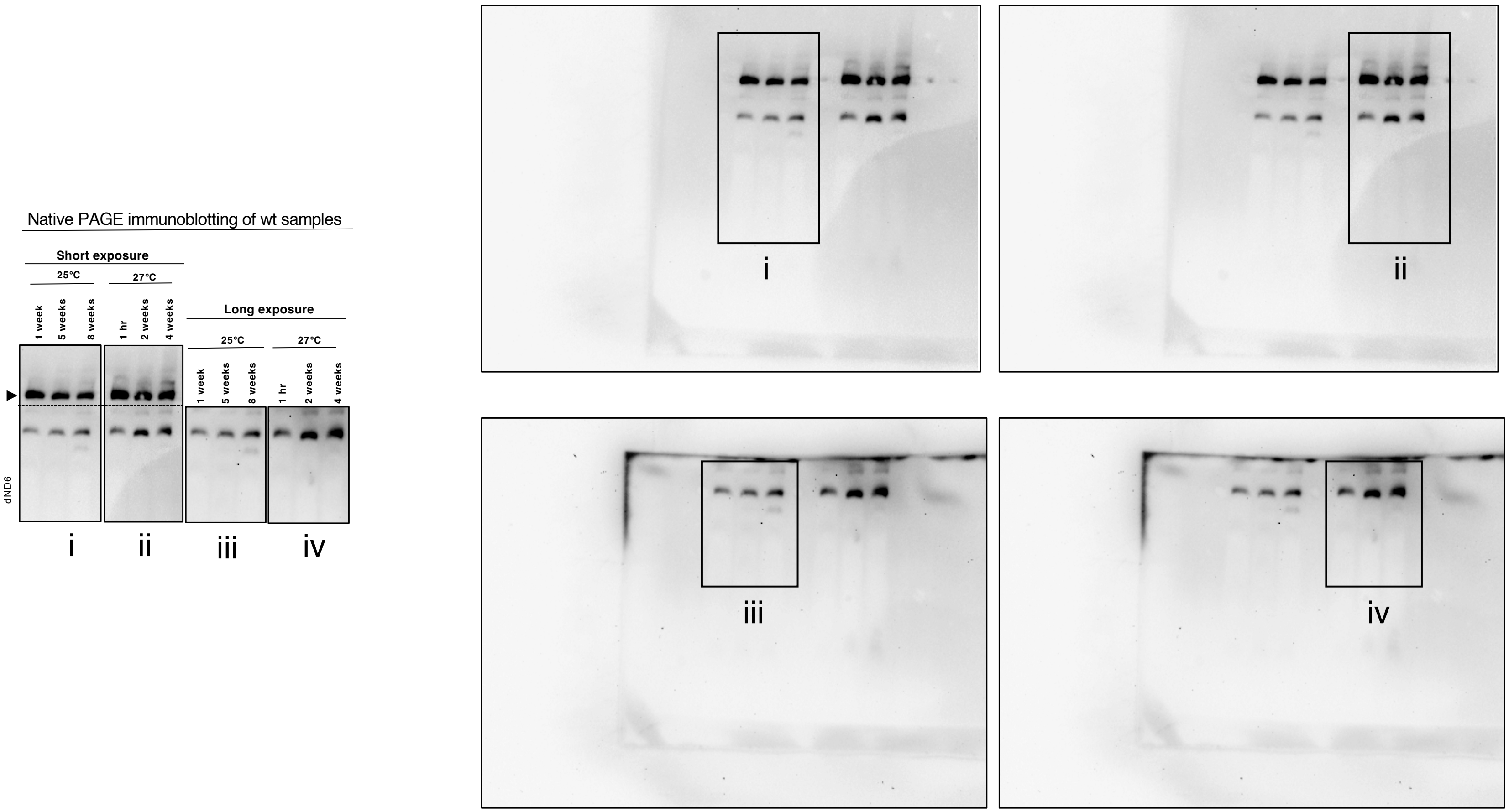
Native PAGE immunoblotting of wt samples
Short exposure
25°C
27°C
1 week
5 weeks
8 weeks
1 hr
2 weeks
4 weeks
Long exposure
25°C
27°C
1 week
5 weeks
8 weeks
1 hr
2 weeks
4 weeks
dND6
i
ii
iii
iv
i
ii
iii
iv

Figure S5G

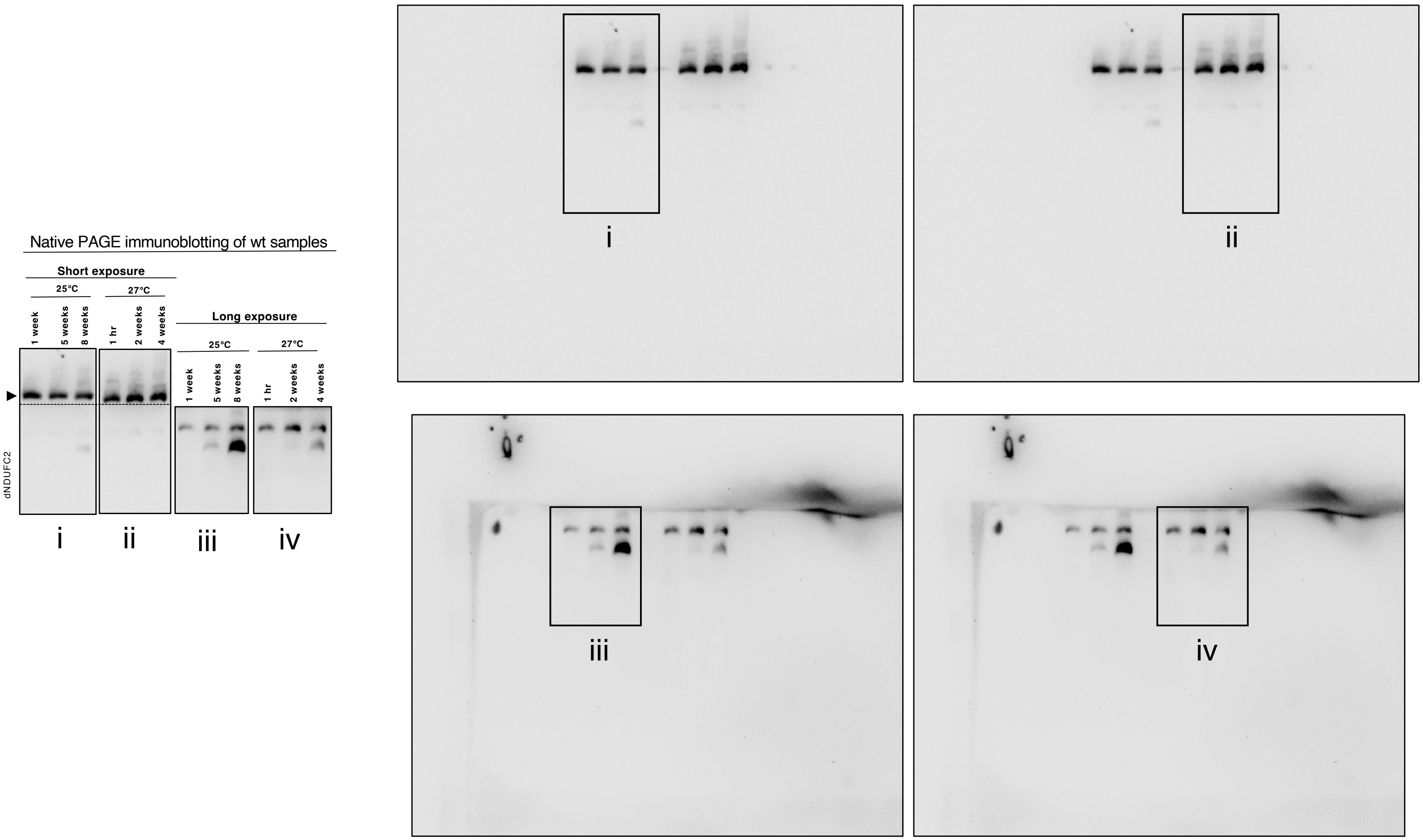

Native PAGE immunoblotting of wt samples
Short exposure
25°C
27°C
1 week
5 weeks
8 weeks
1 hr
2 weeks
4 weeks
Long exposure
25°C
27°C
1 week
5 weeks
8 weeks
1 hr
2 weeks
4 weeks
dNDUFC2
i
ii
iii
iv
i
ii
iii
iv

Figure S5H

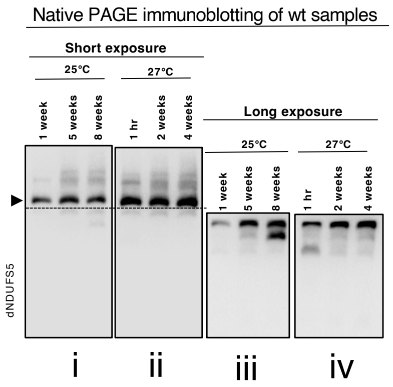
Native PAGE immunoblotting of wt samples
Short exposure
25°C
27°C
1 week
5 weeks
8 weeks
1 hr
2 weeks
4 weeks
Long exposure
25°C
27°C
1 week
5 weeks
8 weeks
1 hr
2 weeks
4 weeks
dNDUFS5
i
ii
iii
iv

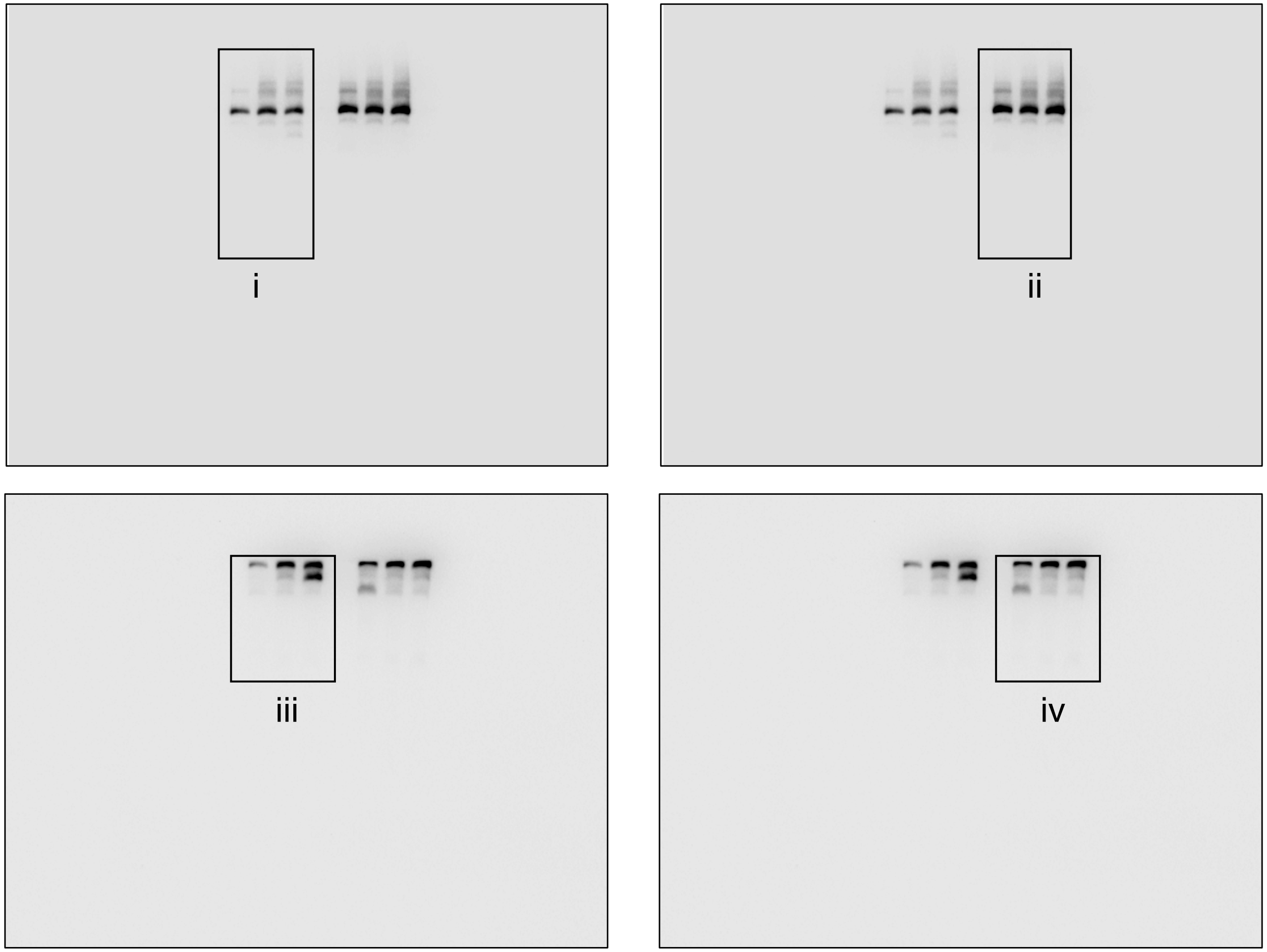
i
ii
iii
iv

Figure S5I

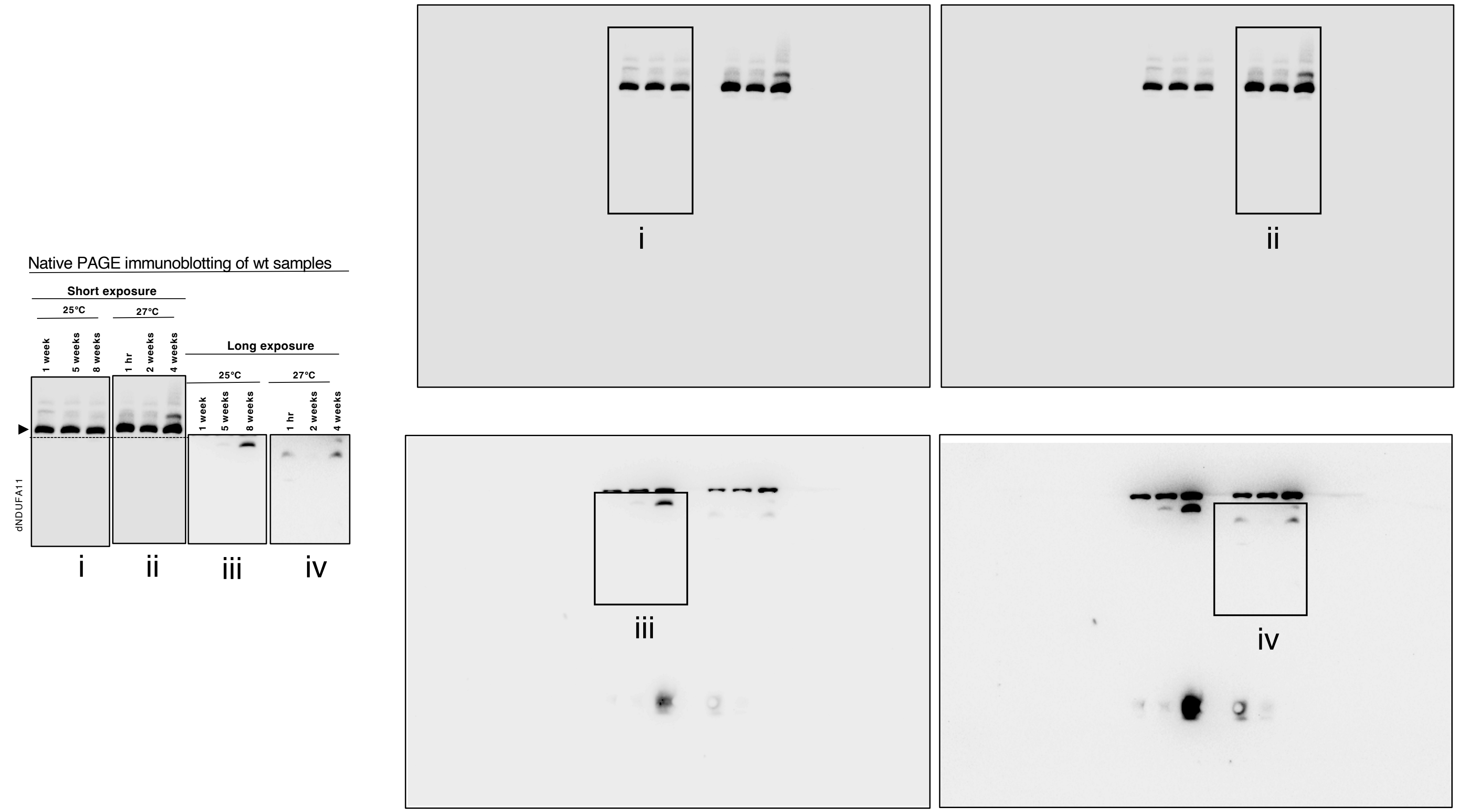

Native PAGE immunoblotting of wt samples
Short exposure
25°C
27°C
1 week
5 weeks
8 weeks
1 hr
2 weeks
4 weeks
Long exposure
25°C
27°C
1 week
5 weeks
8 weeks
1 hr
2 weeks
4 weeks
dNDUFA11
i
ii
iii
iv
i
ii
iii
iv

Figure S5J

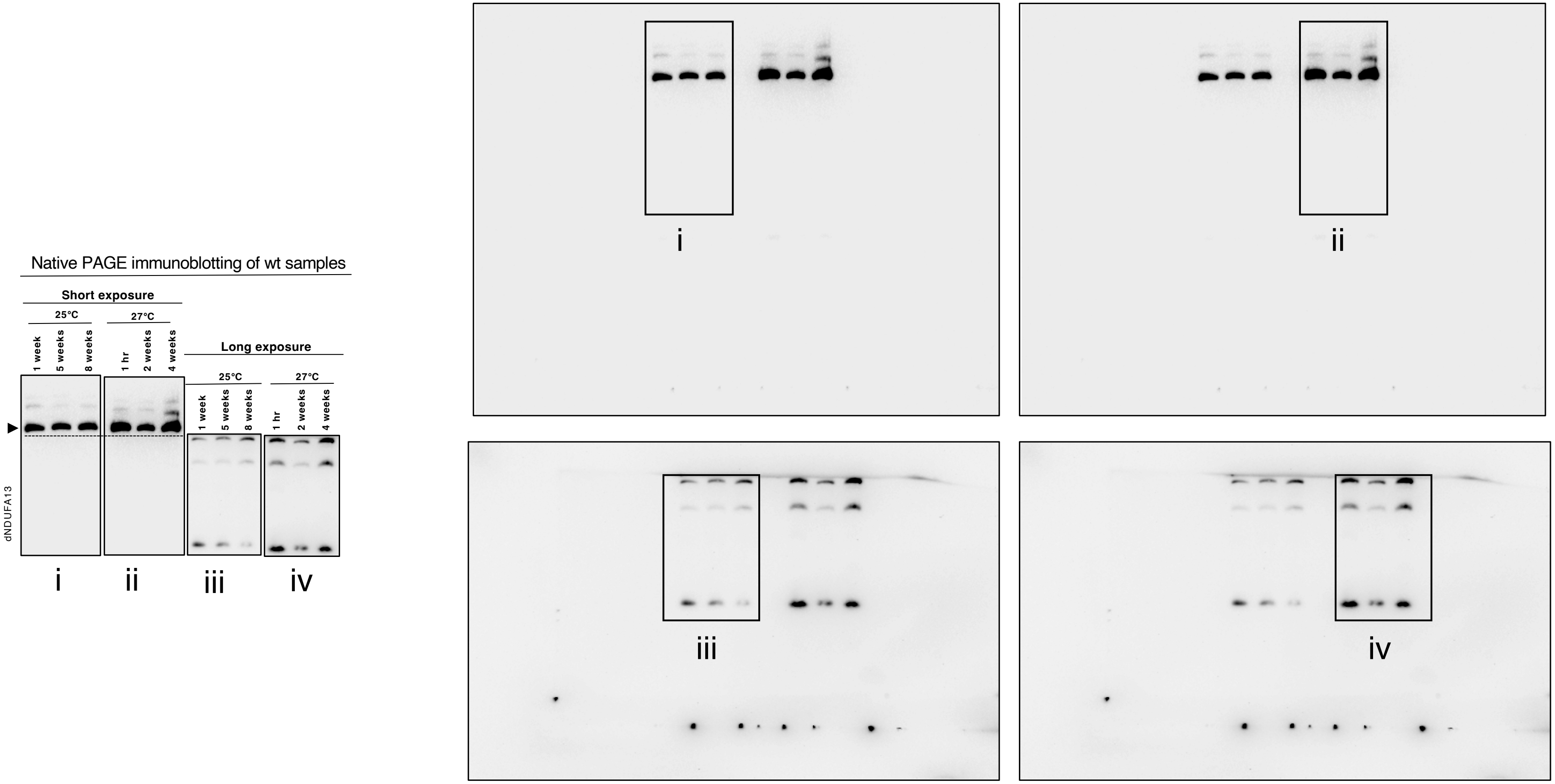

Native PAGE immunoblotting of wt samples
Short exposure
25°C
27°C
1 week
5 weeks
8 weeks
1 hr
2 weeks
4 weeks
Long exposure
25°C
27°C
1 week
5 weeks
8 weeks
1 hr
2 weeks
4 weeks
dNDUFA13
i
ii
iii
iv
i
ii
iii
iv

Figure S5K

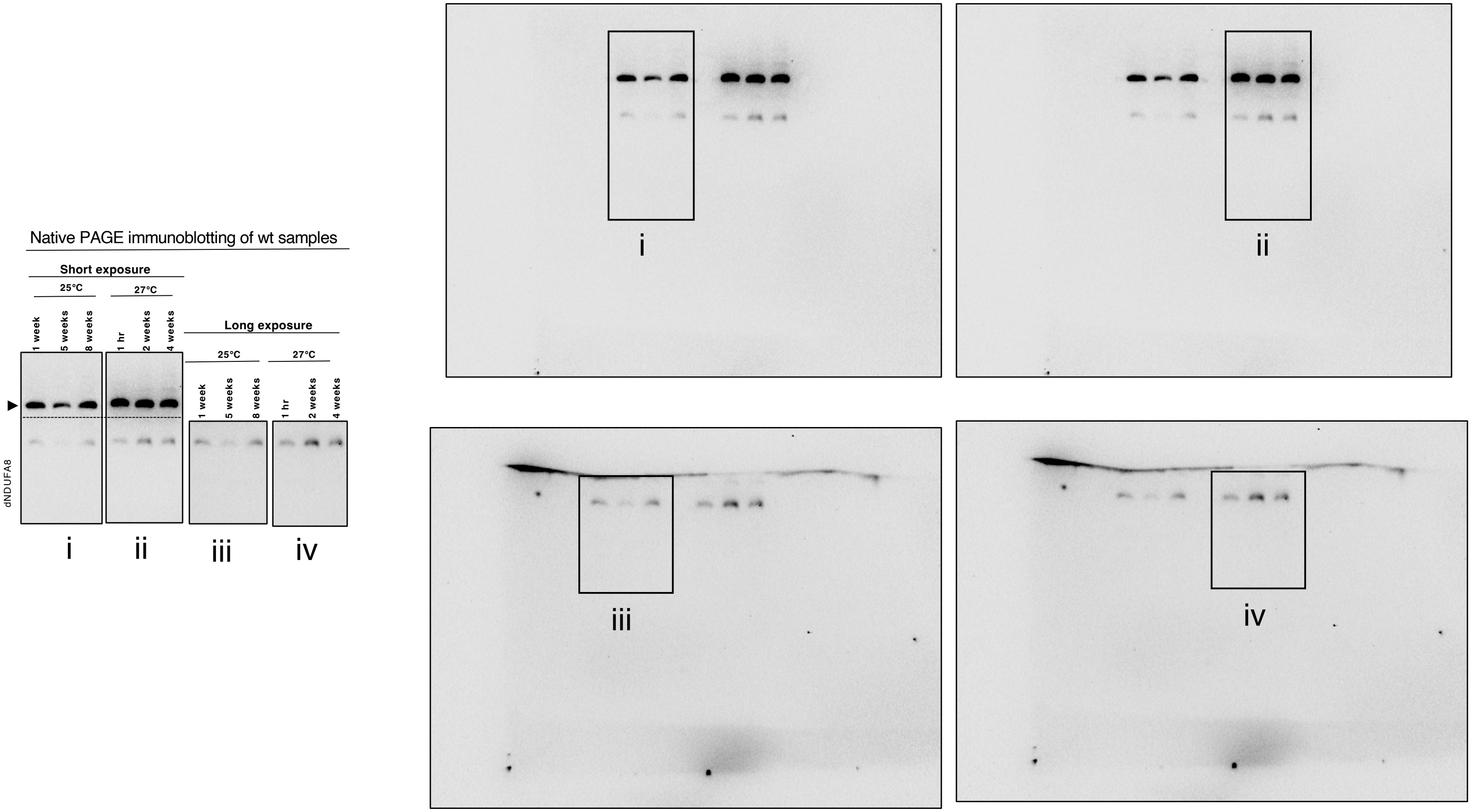
Native PAGE immunoblotting of wt samples
Short exposure
25°C
27°C
1 week
5 weeks
8 weeks
1 hr
2 weeks
4 weeks
Long exposure
25°C
27°C
1 week
5 weeks
8 weeks
1 hr
2 weeks
4 weeks
dNDUFA8
i
ii
iii
iv
i
ii
iii
iv

Figure S5L

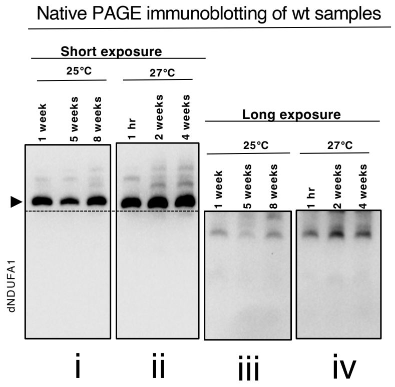

Native PAGE immunoblotting of wt samples
Short exposure
25°C
27°C
1 week
5 weeks
8 weeks
1 hr
2 weeks
4 weeks
Long exposure
25°C
27°C
1 week
5 weeks
8 weeks
1 hr
2 weeks
4 weeks
dNDUFA1
i
ii
iii
iv

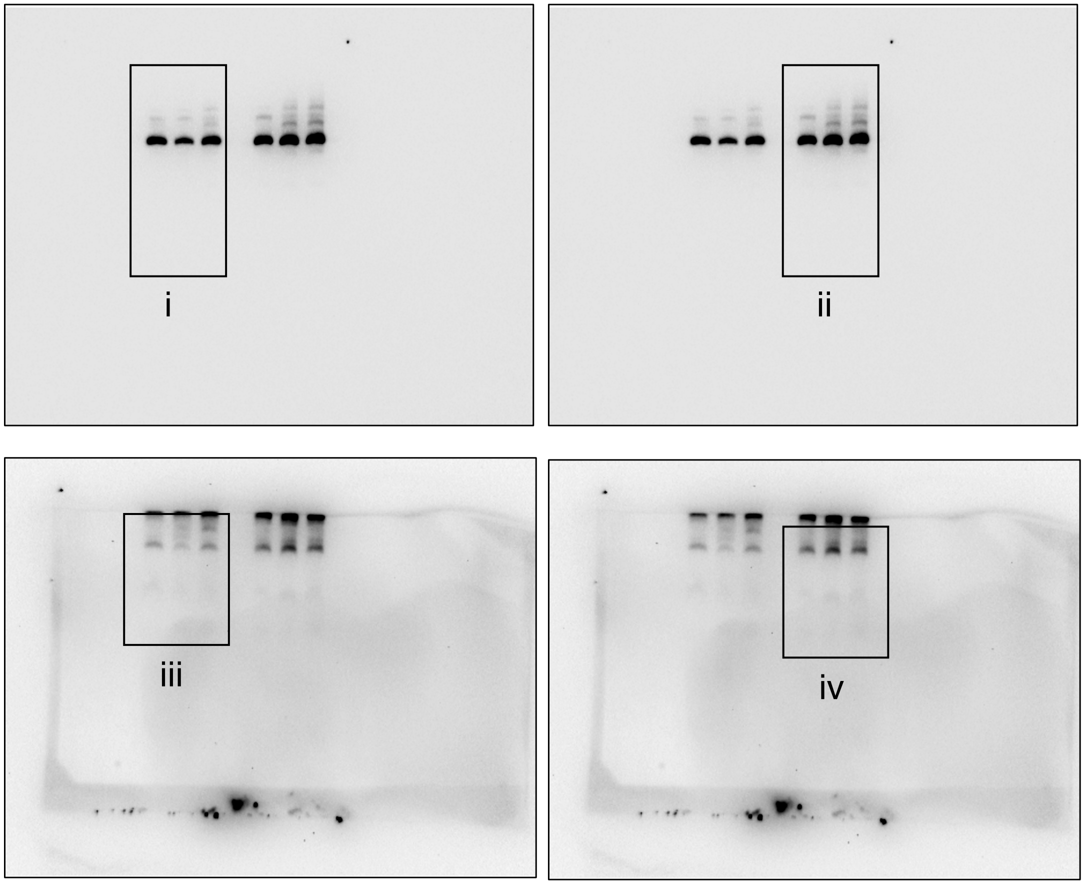

i
ii
iii
iv

Figure S6A

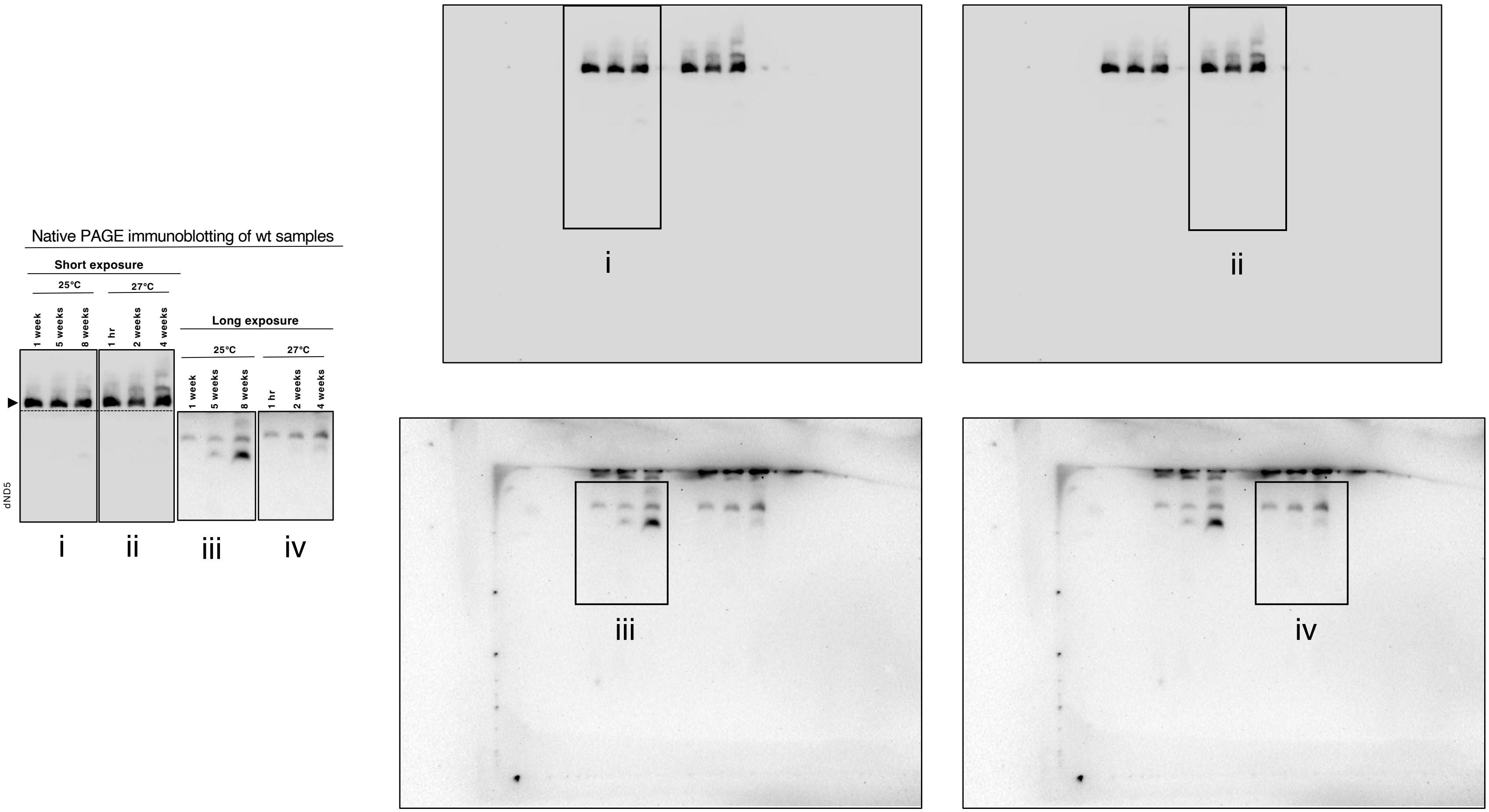

Native PAGE immunoblotting of wt samples
Short exposure
25°C
27°C
1 week
5 weeks
8 weeks
1 hr
2 weeks
4 weeks
Long exposure
25°C
27°C
1 week
5 weeks
8 weeks
1 hr
2 weeks
4 weeks
dND5
i
ii
iii
iv
i
ii
iii
iv

Figure S6B

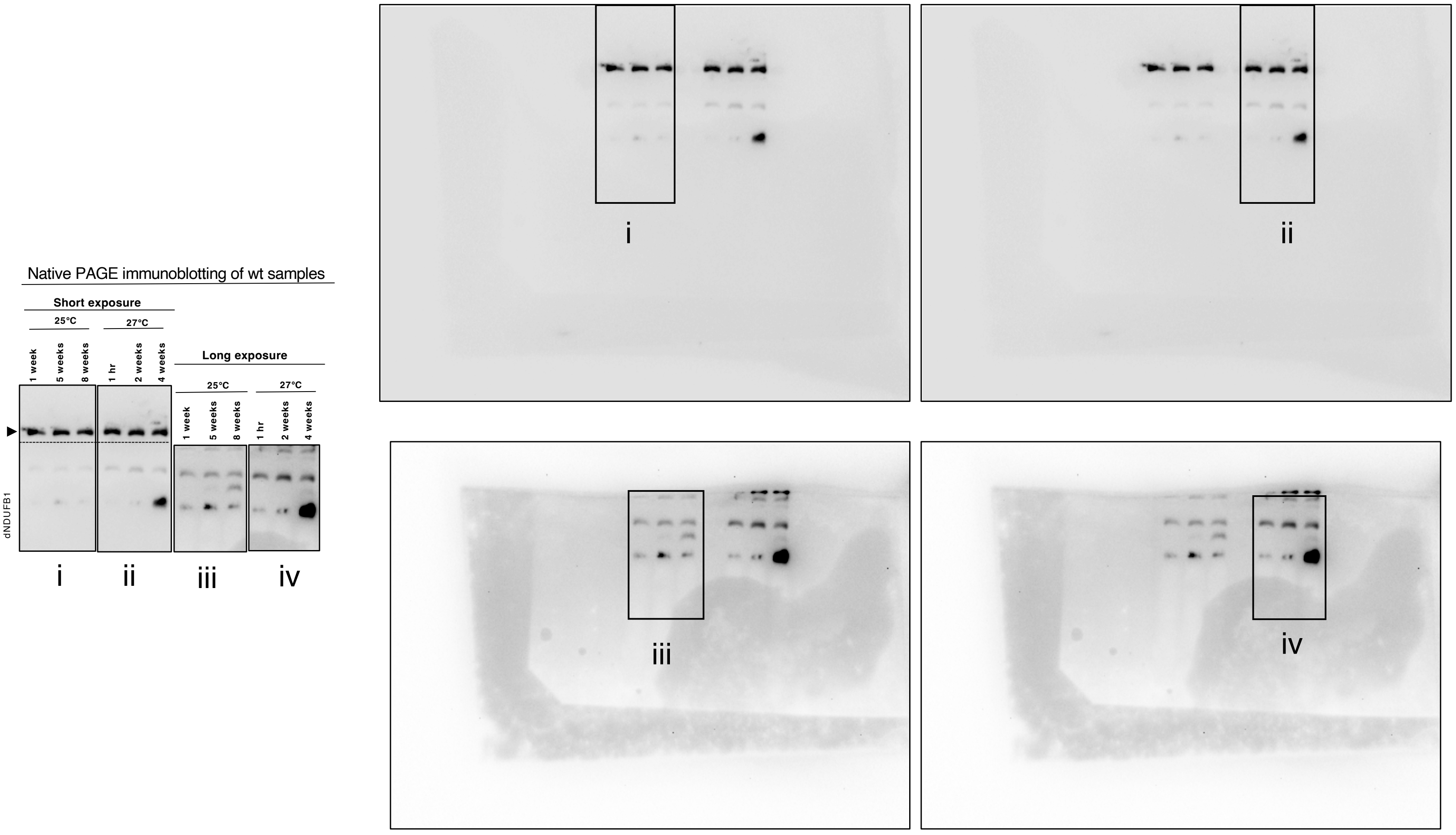
Native PAGE immunoblotting of wt samples
Short exposure
25°C
27°C
1 week
5 weeks
8 weeks
1 hr
2 weeks
4 weeks
Long exposure
25°C
27°C
1 week
5 weeks
8 weeks
1 hr
2 weeks
4 weeks
dNDUFB1
i
ii
iii
iv
i
ii
iii
iv

Figure S6C

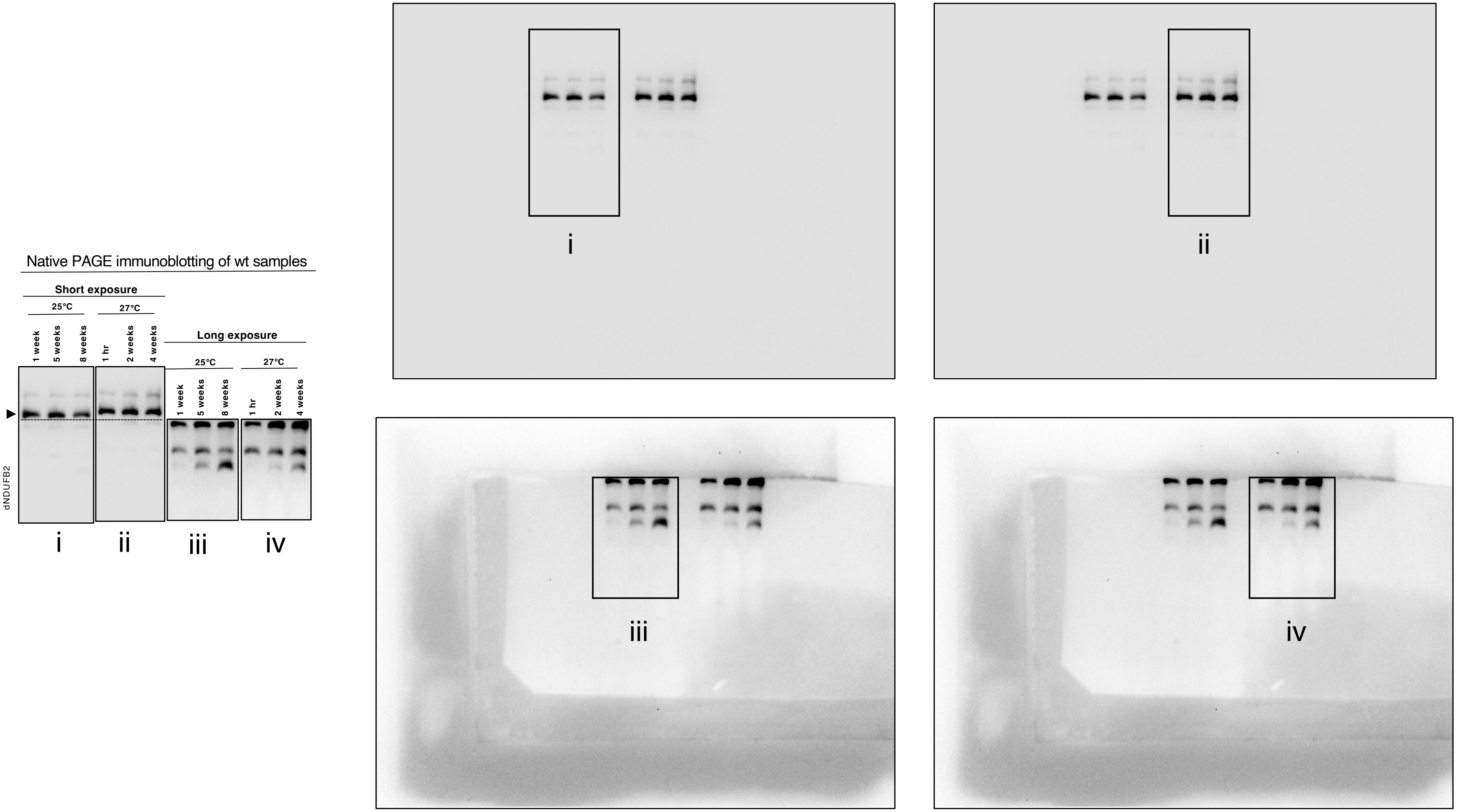

Native PAGE immunoblotting of wt samples
Short exposure
25°C
27°C
1 week
5 weeks
8 weeks
1 hr
2 weeks
4 weeks
Long exposure
25°C
27°C
1 week
5 weeks
8 weeks
1 hr
2 weeks
4 weeks
dNDUFB2
i
ii
iii
iv
i
ii
iii
iv

Figure S6D

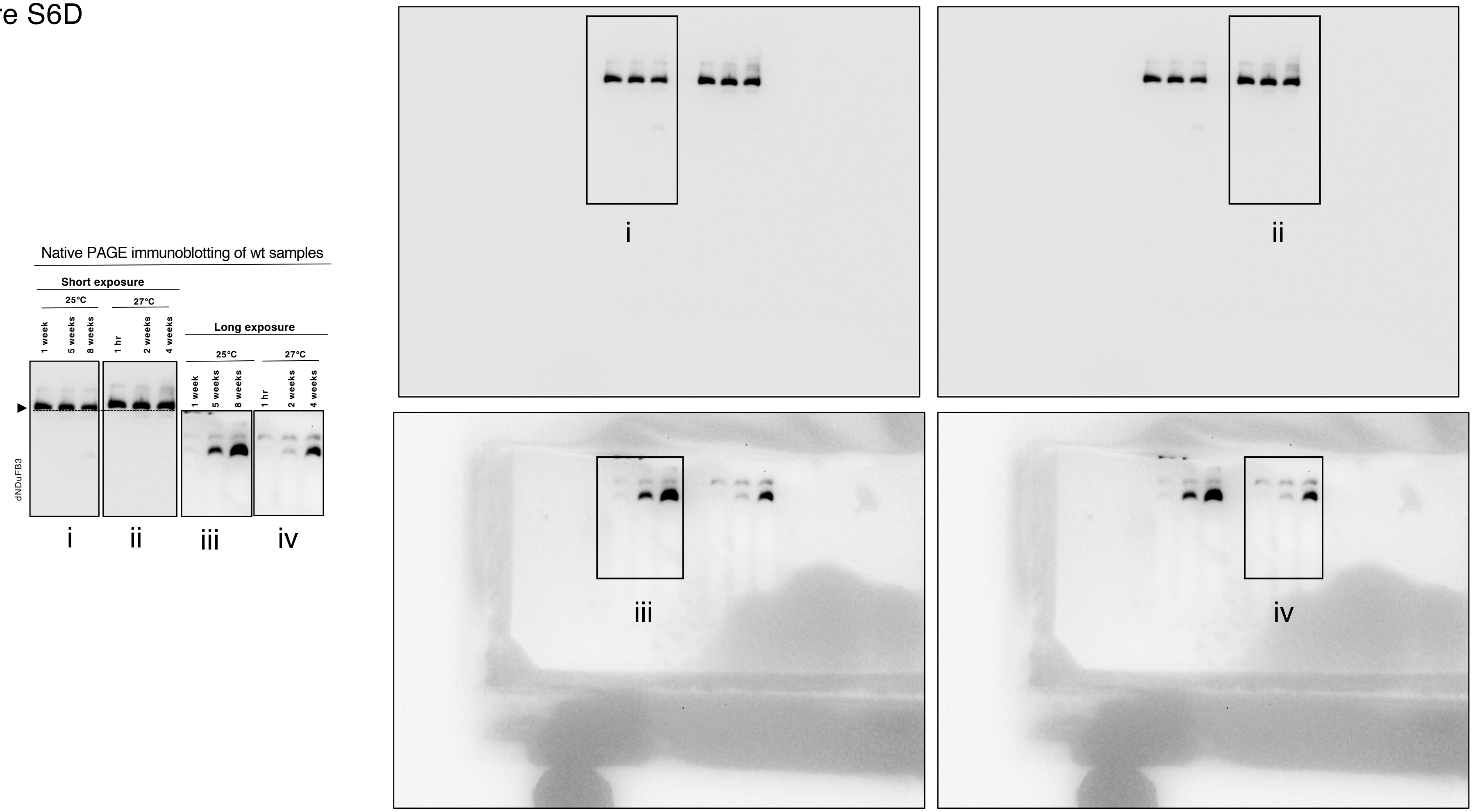

Native PAGE immunoblotting of wt samples
Short exposure
25°C
27°C
1 week
5 weeks
8 weeks
1 hr
2 weeks
4 weeks
Long exposure
25°C
27°C
1 week
5 weeks
8 weeks
1 hr
2 weeks
4 weeks
dNDuFB3
i
ii
iii
iv
i
ii
iii
iv

Figure S6E

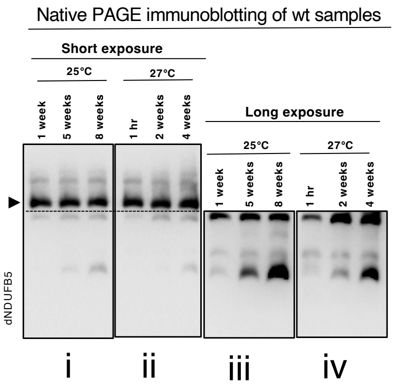

Native PAGE immunoblotting of wt samples
Short exposure
25°C
27°C
1 week
5 weeks
8 weeks
1 hr
2 weeks
4 weeks
Long exposure
25°C
27°C
1 week
5 weeks
8 weeks
1 hr
2 weeks
4 weeks
dNDUFB5
i
ii
iii
iv

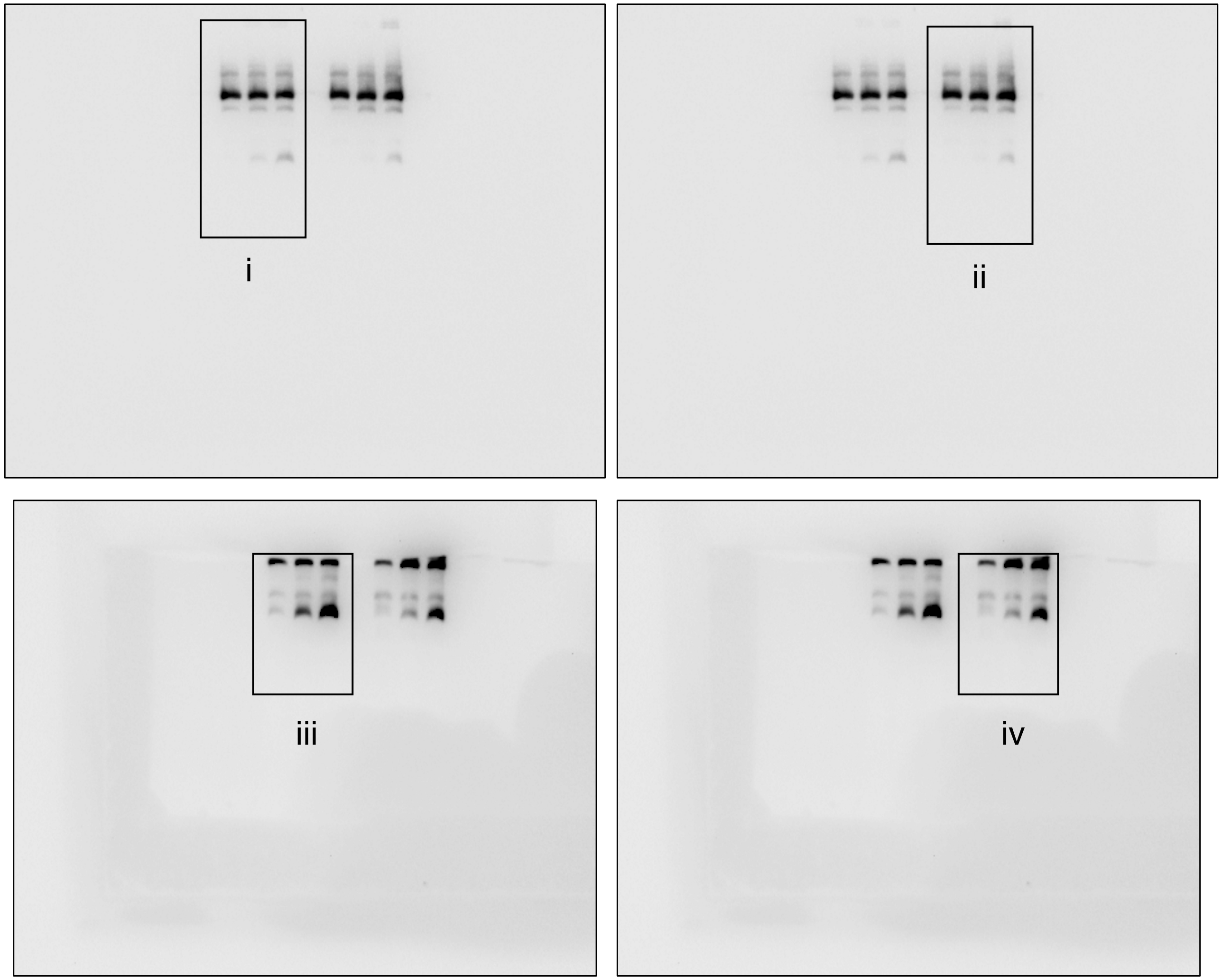

i
ii
iii
iv

Figure S6F

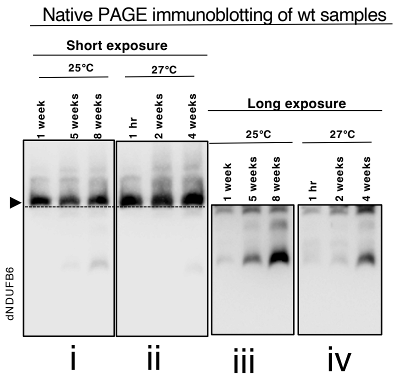
Native PAGE immunoblotting of wt samples
Short exposure
25°C
27°C
1 week
5 weeks
8 weeks
1 hr
2 weeks
4 weeks
Long exposure
25°C
27°C
1 week
5 weeks
8 weeks
1 hr
2 weeks
4 weeks
dNDUFB6
i
ii
iii
iv

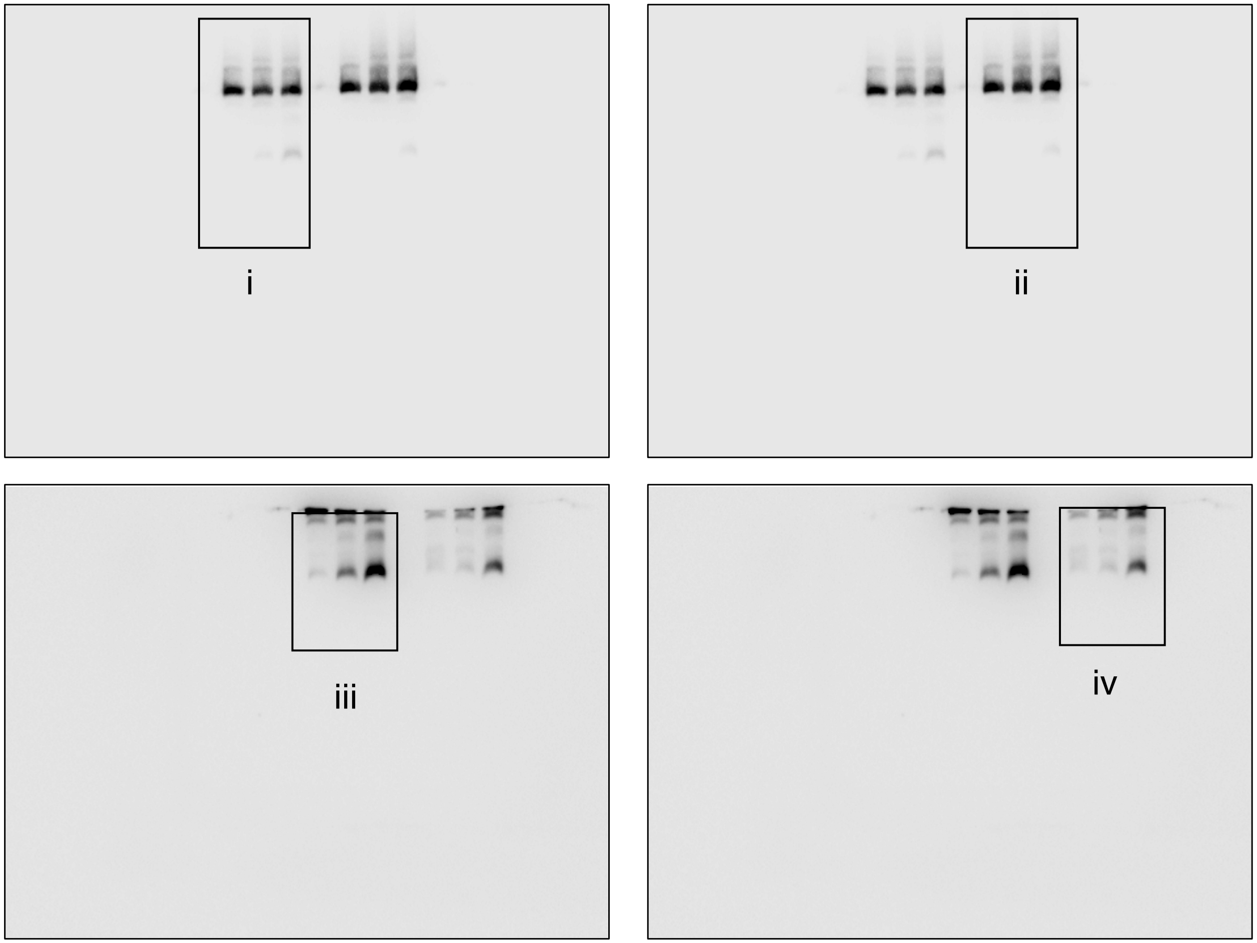
i
ii
iii
iv

Figure S6G

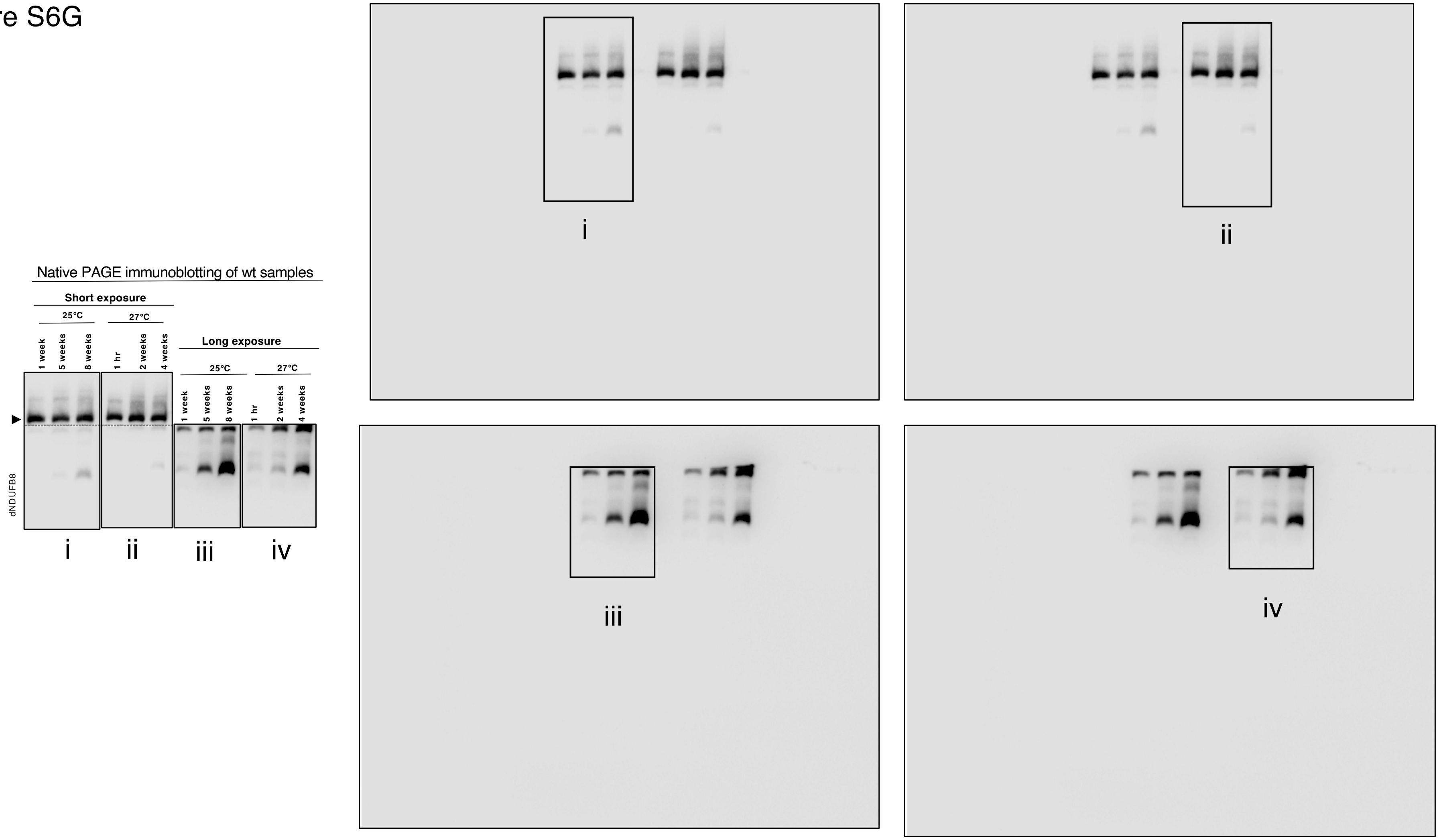
Native PAGE immunoblotting of wt samples
Short exposure
25°C
27°C
1 week
5 weeks
8 weeks
1 hr
2 weeks
4 weeks
Long exposure
25°C
27°C
1 week
5 weeks
8 weeks
1 hr
2 weeks
4 weeks
dNDUFB8
i
ii
iii
iv
i
ii
iii
iv

Figure S6H

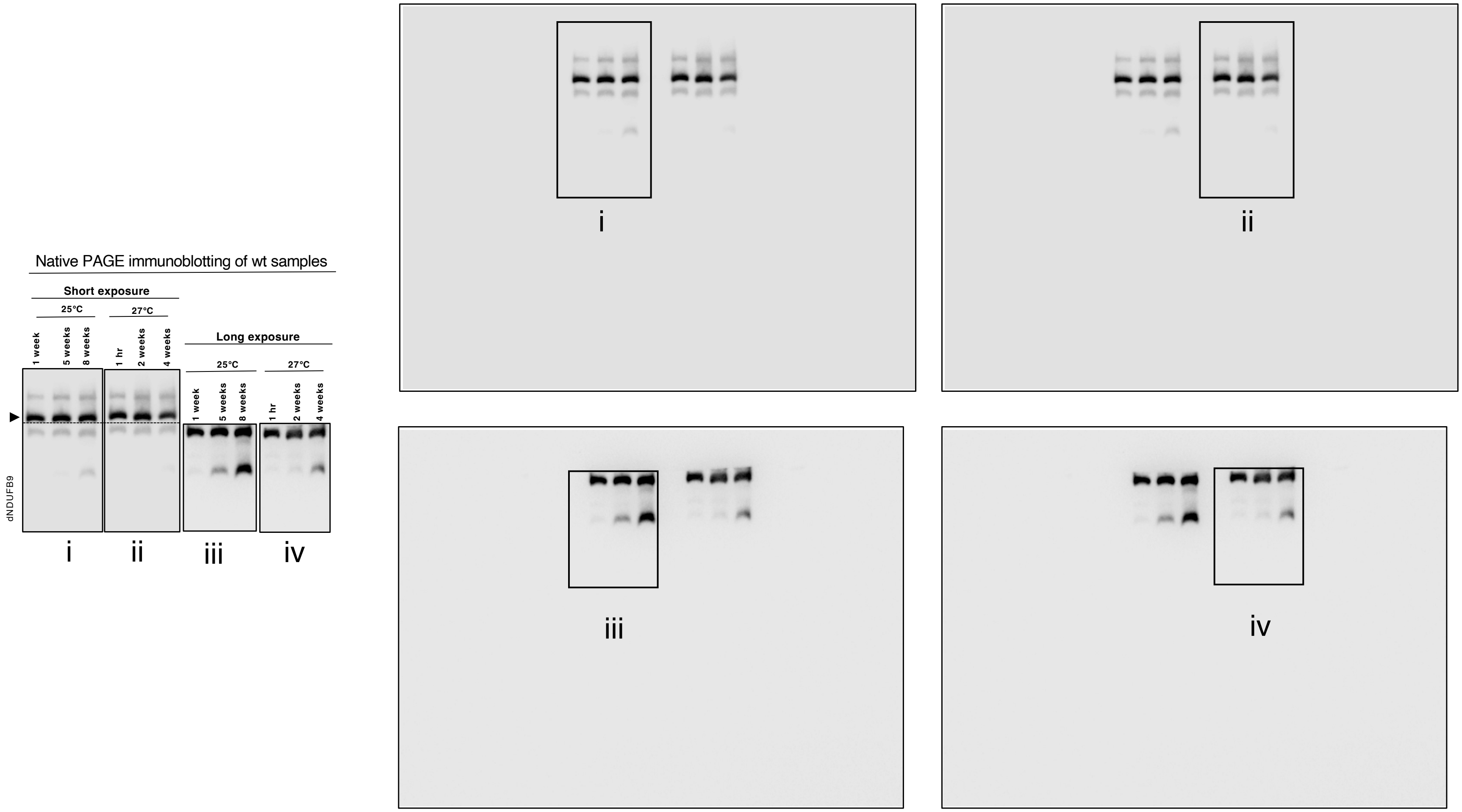

Native PAGE immunoblotting of wt samples
Short exposure
25°C
27°C
1 week
5 weeks
8 weeks
1 hr
2 weeks
4 weeks
Long exposure
25°C
27°C
1 week
5 weeks
8 weeks
1 hr
2 weeks
4 weeks
dNDUFB9
i
ii
iii
iv
i
ii
iii
iv

Figure S6I

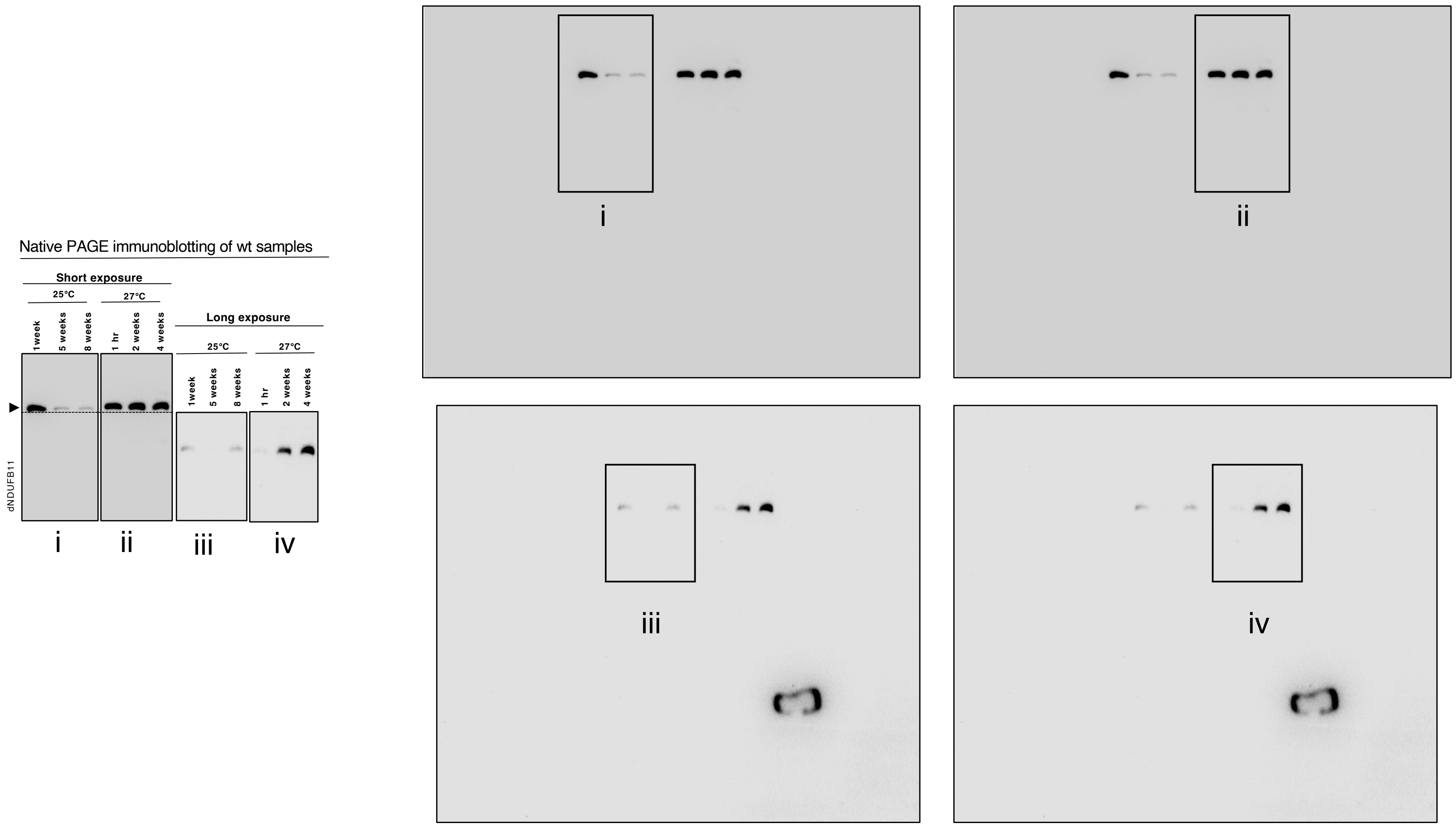

Native PAGE immunoblotting of wt samples
Short exposure
25°C
27°C
1 week
5 weeks
8 weeks
1 hr
2 weeks
4 weeks
Long exposure
25°C
27°C
1week
5 weeks
8 weeks
1 hr
2 weeks
4 weeks
dNDUFB11
i
ii
iii
iv
i
ii
iii
iv

Figure S6J

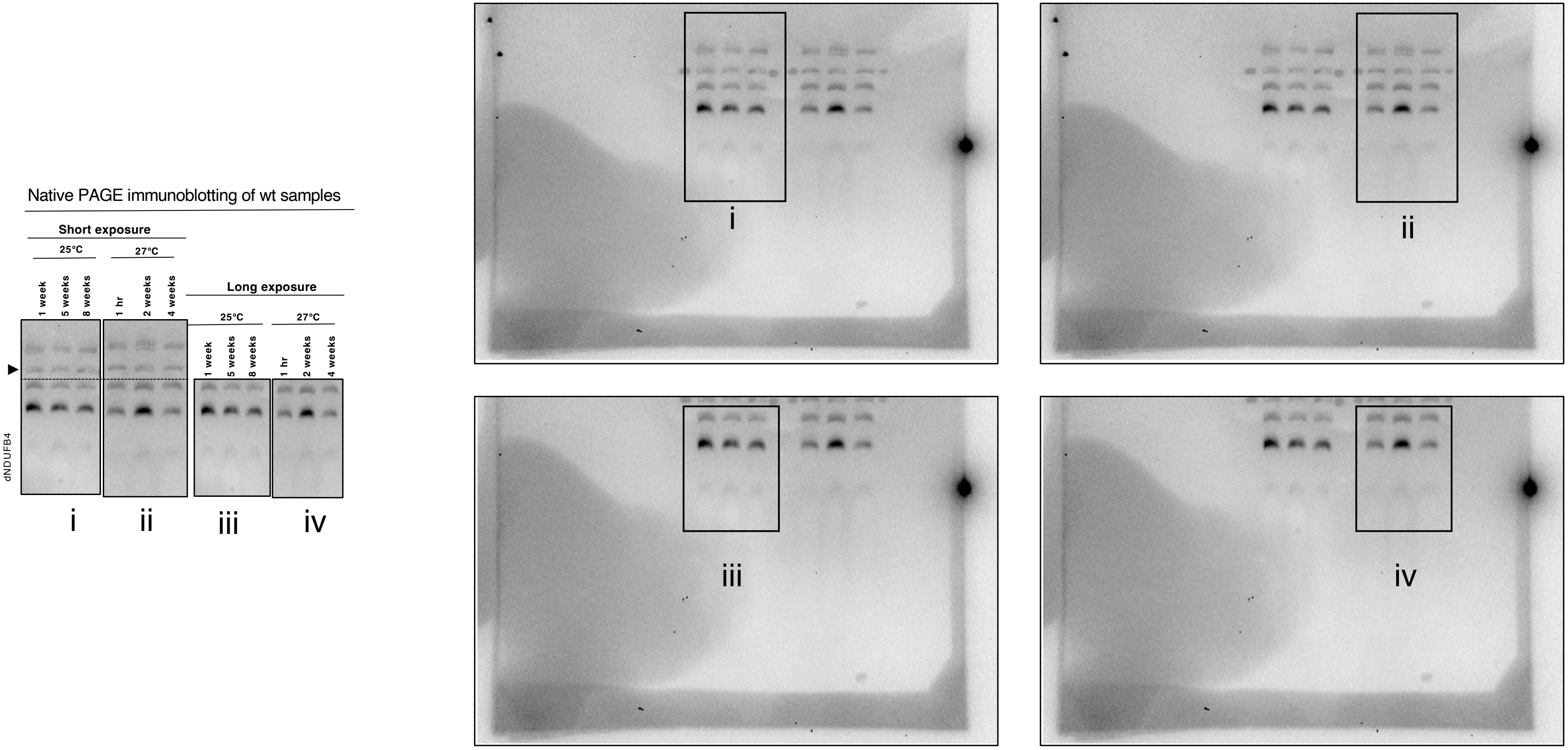

Native PAGE immunoblotting of wt samples
Short exposure
25°C
27°C
1 week
5 weeks
8 weeks
1 hr
2 weeks
4 weeks
Long exposure
25°C
27°C
1 week
5 weeks
8 weeks
1 hr
2 weeks
4 weeks
dNDUFB4
i
ii
iii
iv
i
ii
iii
iv

Figure S6K

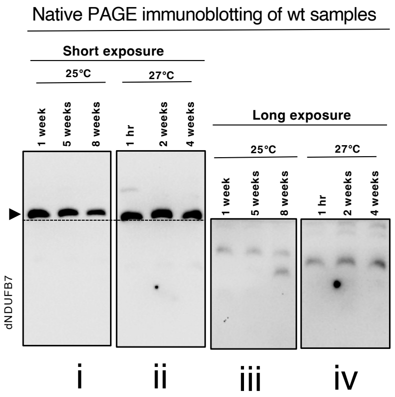
Native PAGE immunoblotting of wt samples
Short exposure
25°C
27°C
1 week
5 weeks
8 weeks
1 hr
2 weeks
4 weeks
Long exposure
25°C
27°C
1 week
5 weeks
8 weeks
1 hr
2 weeks
4 weeks
dNDUFB7
i
ii
iii
iv

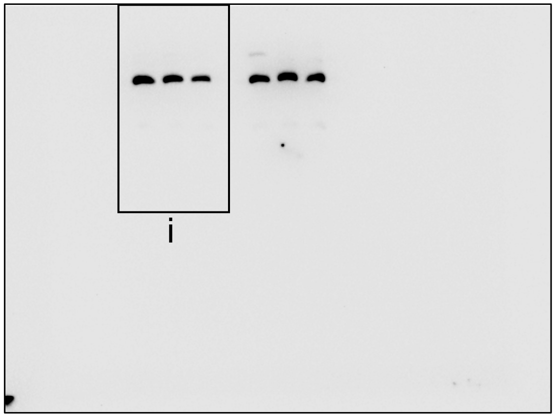
i

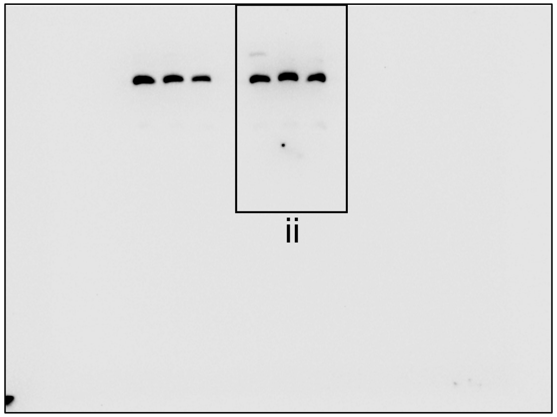
ii

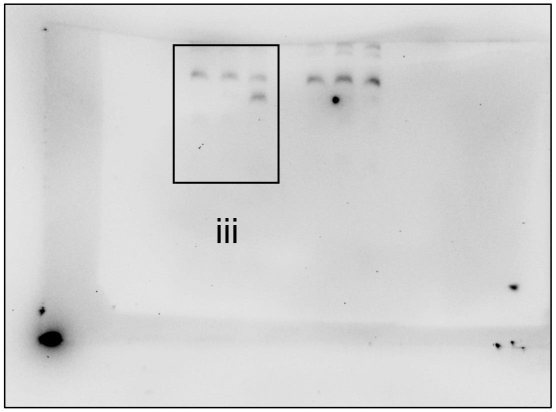
iii

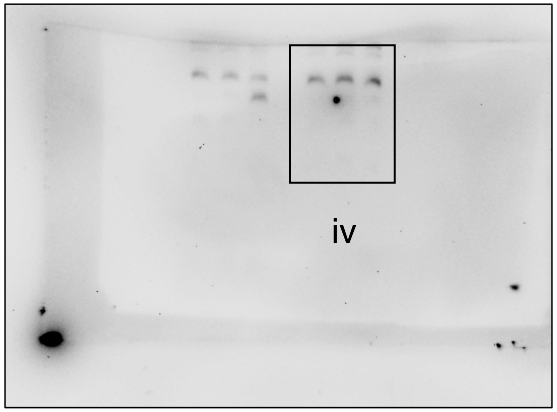
iv

Figure S6L

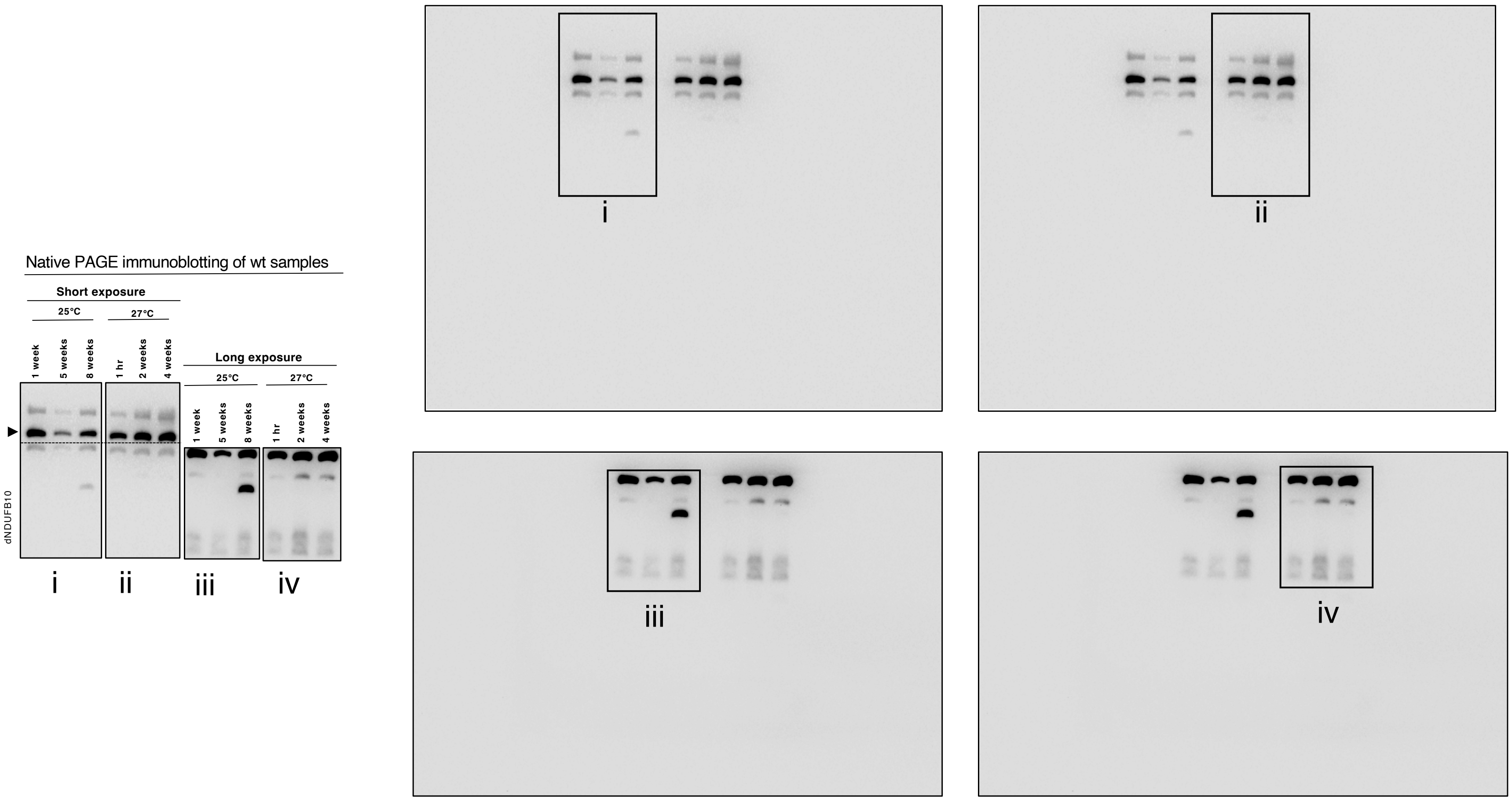
Native PAGE immunoblotting of wt samples
Short exposure
25°C
27°C
1 week
5 weeks
8 weeks
1 hr
2 weeks
4 weeks
Long exposure
25°C
27°C
1 week
5 weeks
8 weeks
1 hr
2 weeks
4 weeks
dNDUFB10
i
ii
iii
iv
i
ii
iii
iv
